# Empirical analysis of pig welfare levels and their impact on pig breeding efficiency—Based on 773 pig farmers’ survey data

**DOI:** 10.1371/journal.pone.0190108

**Published:** 2017-12-27

**Authors:** Yanling Li, Nanjun Wu, Rong Xu, Liqing Li, Wei Zhou, Xianjun Zhou

**Affiliations:** 1 College of Public Management and Law, Hunan Agricultural University, Changsha, China; 2 College of Economics and Management, China Agricultural University, Beijing, China; 3 College of Economics, Hunan Agricultural University, Changsha, China; Universidad Veracruzana, MEXICO

## Abstract

Few studies of the pig production efficiency are from the perspective of animal welfare. Therefore, this study conducted a comprehensive evaluation of pig welfare levels based on survey data from 773 pig farmers from 23 counties in the Chinese provinces of Hunan, Zhejiang, Guangdong, Guizhou, and Shanxi. This study used the Delphi method, Analytic Hierarchy Process (AHP), and Data Envelopment Analysis (DEA)-Tobit regression model to analyze farmers’ pig production efficiency and its influencing factors. This paper found that most farmers’ pig production efficiency is low, and the DEA is invalid. Only 2.9% of pig farmers’ who breed pigs are at the optimal level in terms of welfare, and their production efficiency is relatively high. In contrast, 49.34% of the farmers are at the medium welfare level, and compared with the farmers at the optimal welfare level, these farmers’ pig production efficiency is low. Additionally, the farmers’ age, gender, and number of years of experience with pig breeding have a significant effect. Furthermore, the scale of pig breeding and feeding type, the agriculture facilities for the central treatment of waste in local areas, and the availability of local agricultural science and technology personnel have a considerable influence on pig production efficiency.

## Introduction

In recent years, the improvement of animal welfare has become an important issue in the international community, especially in developed western countries. The concept of guaranteeing animal welfare is deeply embedded in all aspects of daily life, including politics and economics [1]. Many countries and regions have spent considerable resources and have introduced numerous laws on this subject. For example, in the European Union from 2000 to 2008, approximately 70 million euros was spent annually on average to support animal welfare improvement. However, in China, which is affected by the level of economic development and social cultural norms, people do not understand the importance of animal welfare. As a result, the country lacks corresponding policies. Yan, Li, You, Zhang, Liu and Ge [2] conducted an investigation of China’s public attitude on animal welfare and found that only one-third of the Chinese people had even heard of animal welfare. Moreover, different social groups have different attitudes towards animal welfare.

The OIE definition of animal welfare refers to how animals adapt to their environment to meet their basic natural needs. Furthermore, this definition usually covers five aspects, including physical, environmental, health, psychological, and behavioral characteristics. Scientific experiments demonstrate that if an individual animal is in good health, has a comfortable life, adequate nutrition, is safe, is free to express its nature, and does not live in pain, fear, stress, or under threat, the growth potential of the animal will be maximized, and maximum meat output will be achieved [3]. In addition, animal welfare requires more attention regarding animal breeding and slaughter, which increases a producer’s costs. However, this increased cost results in a corresponding increase in the quantity and quality of meat that is produced, a reduction in food safety problems, and an increase in consumer demand, which provides more benefits to producers[4]. Therefore, the measure of the costs and benefits of animal products will determine a producer’s attitude concerning animal welfare [5].

In 1867, Henry drafted and passed a law against animal cruelty. The law broadened the definition of animals and applied to all animals[6]. Since then, various countries have introduced laws and regulations that relate to the protection of animals and have established many animal protection organizations. These have helped people of China understand animal welfare. From a producer’s point of view, improving animal welfare affects the cost of production. For example, Bornett, Guy and Cain [7] showed that changing from slat-flooring to free-space pig farming increases the cost of breeding pigs but improves pig breeding by approximately 4%. Helgesson's [8] study in Sweden showed that the adoption of a mobile slaughtering system resulted in an increase of approximately $0.064 per pound of pork in the northern region and an increase of approximately $0.018 per pound of pork in the south. In addition, some scholars have studied the role of producers in improving animal welfare from the perspective of consumer behavior. A study by Tonsor and Wolf [9] showed that US consumers are willing to pay a premium of 20% for meat products, such as chicken and pork, when producers enhance animal welfare. However, China's animal welfare research began later and lacked useful study results. Wang [10] examined the factors that affect national agriculture development mainly from two aspects, namely, current international animal welfare legislation and some of the obstacles to animal breeding in China; he also analyzed how animal welfare influences the country's international trade through imports and exports. Wang and Gu [4] defined the economic characteristics of the welfare of farm animals based on the consumer’s willingness to pay more by using sample survey data from consumers in Jiangsu Province. The results showed that although the majority of consumers do not have a sufficient understanding of animal welfare, they have common sense and understand that producers have the responsibility to strengthen animal welfare and ensure food safety.

Thus, there has been a lack of studies by scholars on the pig production efficiency of farming from the perspective of animal welfare. Accordingly, this article studies the effects of the levels of pig welfare on breeding efficiency from the perspective of the producer’s behavior and analyzes large-scale farmers’ pig production efficiency from the perspective of pig welfare, and then provides a theoretical basis to make policies and presents some suggestions for the future development of agriculture.

## Evaluation of pig welfare

### Data

The data that are used in this study come from the 2012–2014 summer surveys in 23 counties in Hunan, Zhejiang, Guangdong, Guizhou and Shanxi Provinces. The survey’s objective is to investigate the relevant breeding circumstances of farmers who raise pigs on a scale of more than 30 pigs (i.e., the scale of breeding is based on “The compilation of national agricultural products cost and benefit data,” such as 30 to 100 pigs for small-scale famers, 100 to 1,000 pigs for medium-scale farmers, and more than 1,000 pigs for large-scale farmers). Before the formal investigation, a research group in Changsha County, Hunan Province conducted a pre-survey. The pre-survey results were used to revise and determine the final questionnaire. Additionally, we used one-on-one interviews and the household surveys method. Two townships were selected in each county, one village was selected in each township, and twenty households were selected in each village (In few rare cases, one township for a county, or three village for a township). All the questionnaires were reviewed and recorded by the interviewer to avoid misinterpretation. Finally, we received a total of 773 effective and useful questionnaires to use in this study. All data were collected through one-on-one interviews by a trained interviewer after interviewees were informed and consented to the research process. All interviewers in this study read a consent form on paper and gave verbal confirmation before the interview. This study has been reviewed and approved by the ethics committee of Hunan Agricultural University.

### Methods

To initially analyze the farmers’ pig production efficiency and influencing factors, the following information was used: the construction principle to evaluate pig welfare; domestic and foreign literature; expert advice; in-depth communication with the farmers; and the views and recommendations from managers and veterinarians. These evaluation indicators were adjusted to determine six principles to examine the overall situation of the 773 pig farmers and their animals’ welfare, namely, feeding density, the safe use of feed, epidemic prevention, the frequency of waste disposal, the purchase of agriculture insurance, and the application of farming techniques. As can be seen in Table 1.

**Table 1:** Pig welfare evaluation index system framework.

Objective	Criterion	Index
Evaluation of Pig Welfare (A)	Feeding Condition (B1)	Safety of Feed (C1)
		Drinking Water (C2)
	Housing Environment (B2)	Density of Breeding (C3)
		Waste Disposal (C4)
	Disease Prevention and Control (B3)	Immune Condition (C5)
		Veterinary Staff (C6)
		Drug Storage (C7)
	Risk Control (B4)	Purchase of Agriculture Insurance (C8)
	Management and Technology (B5)	Application of Breeding Technology (C9)

According to the evaluation index system of pig welfare, a questionnaire that included all the evaluation indexes was designed. A total of 55 experts in the veterinary field were surveyed and consulted, and each person completed a questionnaire. In total, 53 questionnaires were collected and used in the statistical analyses. Simultaneously, to improve the accuracy of the responses, pig welfare materials were provided to the experts and were used as the objective basis for expert scoring.

The Delphi method and Analytic Hierarchy Process (AHP) were used to determine the weight of each index to estimate pig welfare. According to the average of the 53 experts’ questionnaire results, a comparison judgment matrix was established. This comparison judgment matrix was written into the MATLAB software, and the maximum eigenvalue, eigenvector, and normalized eigenvector (i.e., weight) were calculated and verified. If CR<0.1, this indicates consistency, and the data are accurate. If CR> 0.1, the matrix must be revised.

### Results

The weight analysis results are shown in Table 2 (S1 File).

**Table 2:** Weight analysis results.

A	B1	B2	B3	B4	B5	W
B1	1	1	1/3	2	3	0.21
B2	1	1	1	2	2	0.23
B3	3	1	1	3	3	0.34
B4	1/2	1/2	1/3	1	1/3	0.09
B5	1/3	1/2	1/3	3	1	0.13

λmax = 5.34 CI = 0.08 RI = 1.12 CR = 0.08<0.1 consistency

Based on the five principles of the animal welfare evaluation, the largest influence involves disease prevention and control, which is 33.54%, and this result indicates that a healthy body is the foundation of pig welfare. Second, the influence of the housing environment on live pigs is also high. Although the purchase of insurance and the application of breeding technology have little impact on the welfare of the animals, the five evaluation principles fully reflect the concerns of the veterinarians regarding farm pig welfare, which reflect well the basic situation of the welfare of live pigs.

According to the results of the pig welfare evaluation index, the feeding principle scores 21, the animal housing environment principle scores 23, the disease prevention and control principle scores 34, the risk control principle scores 9, the principle of technical application scores 13, and the entire evaluation table scores 100 points. The results of the survey of 773 farmers’ pig welfare levels are shown in Table 3 (S2 File).

**Table 3:** Survey results regarding 773 farmers’ pig welfare levels.

Type	Proportion of Farmers’ Pig Welfare Levels			
	Poor	Medium	Good	Optimal
Number of Farmers	292	381	78	22
Proportion of Farmers	37.77%	49.29%	10.09%	2.85%

Note: A total score < 60 is poor, a total score of 60 to 75 is medium, a total score of 75 to 85 is good, and a total score of 85 to 100 is optimal.

The results show that only 2.85% of the farmer's pig welfare level is optimal, and the majority of farmers had pig welfare levels of poor and medium. Overall, the pigs’ welfare level is not good.

## An empirical analysis of agriculture efficiency

### Method

In an economic model with multiple inputs to one output, if the economy can utilize its resources to maximize output, this result is considered to be efficient [11]. In this simple economic model, it can be assumed that the input is *X* (x_1_, *x*_2_, *x*_3_ …, *x*_*h*_), the output is y, and the relationship between inputs and outputs is described by the production function.

$$\text{y}=f\left(x\right)=f({\text{x}}_{1},x_{2},x_{3}\ldots ,x_{h})$$ (1)

The DEA model is a typical method that is used to study the comprehensive efficiency of multiple outputs under multiple inputs. Assume that there are h inputs, m outputs, and n measured units. Then, the *C*^2^*R* model can be constructed as follows.

$$\text{min}\left[\theta -\varepsilon (e^{T}s^{-}+e^{T}s^{+})\right]$$

$$\text{s.t.}\sum_{\text{j}=\text{1}}^{\text{n}}\lambda_{\text{j}}x_{j}+s^{+}=\theta x_{0}$$

$$\sum_{\text{j}=\text{1}}^{\text{n}}\lambda_{\text{j}}{\text{y}}_{j}-s^{+}=y_{0}$$

$$\lambda_{\text{j}}\geq 0,j=1,\text{.....},n$$

$${\text{s}}^{-}\geq 0,s^{+}\geq 0$$

In the formula (2), x_0_ is an input indicator, and y_0_ is an output indicator for each unit’s combination coefficient. *ε* is non-Archimedes infinitesimal quantities, which in practical application, is often taken as a very small positive number, such as 10^−*ε*^. e^*T*^ is a unit row vector. In an empirical analysis, the main indicators that are used in an efficiency evaluation include *θ*, s^−^, *s*^+^; *θ* is the efficiency evaluation index, which is usually called the efficiency coefficient. s^−^, *s*^+^ are the relaxation variables. If *θ* < 1 but s^−^, *s*^+^ are not all 0, DEA is considered to be inefficient, that is, the current output can be achieved with less than the existing input. If *θ* = 1, s^−^, *s*^+^ are non-0 so that the unit DEA is weak. If *θ* = 0 and s^−^, *s*^+^ are 0, the unit DEA is efficient, that is, the input should not be increased or reduced under the existing output.

The Variable Returns to Scale (VRS) model is one form of a DEA model. Assume that the model has n units, and the basic form of the model can be constructed as follows.

$$Min\theta_{v}$$

$$s.t.\sum_{i=1}^{n}r_{i}X_{i}\leq \theta_{v}X_{0}$$

$$\sum_{i=1}^{n}r_{i}X_{i}\geq Y_{0}$$

$$\sum_{i=1}^{n}r_{i}=1$$

$$r_{i}\geq 0,i=1,2,\ldots ,n,\theta_{v}\geq 0$$

In the formula, *ϑ*_*v*_ represents the efficiency of the pig breeder and assumes that the evaluation unit is variable in scale; *X*_0_ is the input variable, *Y*_0_ is the output variable, and *r*_*i*_ is the weight that is assigned to each evaluation unit.

DEA is a "data-oriented" method that is used to measure the performance and relative efficiency of a set of decision-making units (DMUs) with multiple inputs and outputs [12]. In this paper, DEA is used to analyze the efficiency of pig production, and we will use the VRS model to analyze the pig production efficiency of the pig farmers.

The DEA method cannot find the factors that affect efficiency. To solve the problem of efficiency distribution, the Tobit model is very effective [13]. The Tobit model was presented by Tobin who is an economist and a Nobel Prize winner in economics. The main advantage of this model is that the limited dependent variable can be defined. The general formula is the following.

$$y_{i}^{*}=\beta^{T}X_{i}+\varepsilon_{i}$$

$$y_{i}=y_{i}^{*}\begin{array}{ccc} & if & y_{i}^{*} \end{array}>0$$

$$y_{i}=0\begin{array}{ccc} & if & y_{i}^{*} \end{array}\leq 0$$

In this formula,$y_{i}^{*}$ is the explanatory variable, *y*_*i*_ is the dependent variable that is obtained for the survey, *X*_*i*_ is the explanatory variable, *β*^*T*^ is the correlation coefficient vector, and *ε*_*i*_ is the independent and Standard Normal Distribution. When $y_{i}^{*}>0$ and $y_{i}=y_{i}^{*}>0$, *y*_*i*_ is considered the "unrestricted" observation value. When $y_{i}^{*}\leq 0$ and *y*_*i*_ = 0, *y*_*i*_ is a "restricted" observation value.

Therefore, this paper uses 773 pig farmers as the DMU and the DEA method to measure the efficiency of the pig farmers. Then, according to the statistical results of pig production efficiency, the Tobit model is used to analyze the factors that affect the production efficiency of pig farmers.

### Variables and samples

According to the characteristics of pig production and DEA calculation requirements, the controllable variables that affect pig production efficiency are pig breeds, pig nutrition, epidemic prevention measures and management, etc. Therefore, this study uses the sum of the output value of the main product and the by-product of each pig as the output variable. The input index is mainly based on the input cost of each young piglet, input costs of feed (i.e., fine feed costs, green roughage costs, and feed processing costs), labor costs (i.e., family labor costs according to the market price discount and employment expenses), veterinary epidemic prevention costs (i.e., medical and epidemic prevention, technical service fees, etc.), death damages, expenditures on power, and other expenses (including the depreciation of fixed assets and equipment maintenance costs, etc.). The specific indicators are shown in Table 4 (S3 File).

**Table 4:** Average input and output of the 773 pig farmers every year for each pig.

Index Type	Index Name	Meaning and Unit	SD	Mean Value (rmb)	Max	Min
Output	output value of pig	net output for main product and by-product of each pig (rmb/head)	1380.56	1,721.02	1803.72	1584.74
Input	young piglet cost	average cost per young piglet (rmb/head)	359.29	500.00	568.70	468.40
	feed costs	feed costs per pig from young piglet to market (rmb/head)	1233.42	866.17	989.37	614.13
	labor costs	family labor costs and employment expense (rmb/head)	546.19	167.11	187.76	101.84
	epidemic prevention cost	costs of veterinary drugs and epidemic prevention (rmb/head)	124.31	15.69	21.40	2.31
	death penalty	death damages (rmb/head)	35.04	11.73	14.05	8.51
	power costs	electricity, coal and other power costs (rmb/head)	21.06	6.93	8.37	2.56
	other costs	depreciation of fixed assets and equipment maintenance costs (rmb/head)	112.83	17.81	44.68	8.03

Source: Survey on pig breeding in 5 Provinces (23 counties) from 2012 to 2014.

For the status of the pig breeding situation of the 773 scale farmers, the average weight per pig is 114.24 kg, and the total output value is 1,721.02 rmb. For the input variables, the average price is 500.00 rmb per young piglet. The average cost per pig for fine feed is approximately 856.83 rmb, green roughage is 6.13 rmb, and feed processing costs are approximately 3.21 rmb. Therefore, a pig’s feed costs are approximately 866.17 rmb. The cost per pig for labor is 167.11 rmb, veterinary epidemic prevention is approximately 15.69 rmb, the death penalty is approximately 11.73 rmb, power is approximately 6.93 rmb, and other costs are approximately 17.81 rmb.

In this paper, we selected the integrated production efficiency values (Y, 0≤Y≤1) of the 773 pig farmers as the dependent variable. According to the related theory and the research literature, we selected the relevant indicators other than inputs and outputs to analyze the factors that affect the production of pigs, including the basic characteristics of the farmers, family characteristics, the pigs’ welfare level, and the comprehensive situation of the pigs’ welfare, as shown in Table 5 (S4 File). The characteristics of the farmers include age, gender, and educational level. The characteristics of the family mainly include the farmers’ pig breeding years and the proportion of pig breeding that accounted for total revenue. The features of the pigs' welfare level include the pig farming scale, distance from town, and feeding type. Additionally, this study examined whether there are agriculture facilities for the central treatment of waste in the village, as well as agricultural science and technology personnel. The comprehensive welfare state of the pigs determines the results of the comprehensive evaluation of their welfare.

**Table 5:** Explained variables and statistical description.

Variable types	Variable name	Variable definitions	Mean	SD.	Max	Min
basic characteristics of farmers	age	age (years)	47.83	7.23	64	27
	gender	women = 0; men = 1	0.67	0.47	1	0
	education level	elementary school level and below = 1; junior high school = 2; high school = 3; university or above = 4	2.46	0.79	4	1
family characteristics	breeding years	cumulative number of years from the beginning of breeding to the present (years)	12.85	3.75	24.83	2.17
	revenue	proportion of pig breeding that accounted for total revenue (%)	64.23	19.83	92.16	14.54
welfare variable characteristics of live pigs	farming scale	small = 1; medium-sized = 2; large = 3	1.83	0.71	3	1
	housed livestock	open = 0; enclosed = 1	0.34	0.47	1	0
	waste disposal equipment	do not have = 0; have = 1	0.21	0.41	1	0
	science and technology personnel	insufficient = 0; adequate = 1	0.43	0.5	1	0
comprehensive situation of pig welfare	comprehensive pig welfare level	poor = 1; medium = 2; good = 3; optimal = 4	1.78	0.74	4	1

Note: The scale of farming is based on the "National Compilation of Costs and Profits of Agricultural Products." The number of livestock pigs is 30 to 100 per year for small-scale farmers, 100 to 1,000 for medium-sized farmers, and more than 1,000 for large-scale farmers. The situation regarding local agricultural science and technology personnel is based on their own experience.

## Results

The results of the comprehensive technical efficiency of the 773 pig breeders in the five provinces, which were calculated using DEAP2.1 software, are shown in Tables 6 and 7 (S5 File).

**Table 6:** Production efficiency results of the 773 pig farmers in the five provinces of Hunan, Zhejiang, Guangdong, Guizhou, and Shanxi.

Efficiency value	Number of samples	Proportion (%)	Cumulative proportion (%)
0.893–0.904	2.00	0	0.00
0.905–0.914	2.00	0	0.01
0.915–0.925	13.00	2%	0.02
0.926–0.936	48.00	6%	0.08
0.937–0.947	118.00	15%	0.24
0.948–0.957	123.00	16%	0.40
0.958–0.968	123.00	16%	0.55
0.967–0.979	138.00	18%	0.73
0.980–0.989	107.00	14%	0.87
0.990–1.000	99.00	13%	1.00
Minimum	0.89		
Maximum	1.00		
Mean	0.96		
SD.	0.02		
Sample	773		

**Table 7:** The production efficiency results of each welfare level table.

Type	Productivity of different welfare levels (mean)			
	poor	medium	good	optimal
Comprehensive technical efficiency	0.345	0.568	0.626	0.983

The Tobit model was used to analyze the comprehensive efficiency of agriculture production with Eviews7 statistical software. These results are shown in Table 8.

**Table 8:** Statistical analysis.

variable	Whole model			Simplest model		
	coefficient	SD	Z	coefficient	SD	Z
Intercept item	0.98084***	0.01049	93.47964	0.97304***	0.00356	273.12380
Age	-0.00015	0.00018	-0.81803	0.00370**	0.00160	2.31367
Gender	0.00383**	0.00175	2.18801			
Education level	-0.00163	0.00143	-1.13859	-0.00052***	0.00020	-2.62543
Pig breeding years	-0.00044***	0.00025	-1.79103			
Proportion of pig breeding accounting for total revenue	0.00008	0.00008	0.98509	-0.00420***	0.00139	-3.03323
Farming scale	-0.00594***	0.00213	-2.79507	0.01343***	0.00204	6.57747
Feeding form	0.01233***	0.00222	5.55014			
Waste disposal equipment	-0.00114	0.00266	-0.42786	-0.00423***	0.00157	-2.69394
Science and technology personnel	-0.00440***	0.00158	-2.78692			
Comprehensive pig welfare level	0.00163***	0.00160	1.01747			
Log likelihood	1938.822				1936.671	
AIC	-4.985				-4.993	

Note:

***p<0.01,

**p<0.05

From the results, it can be observed that the average comprehensive technical efficiency of the farmers is 0.96. A total of 13% of the 773 farmers’ production efficiency value is close to 1 or is 1, which means that these farmers are efficient in the DEA method. The remaining farmers are in a relatively inefficient state regarding pig breeding production, which suggests a need for considerable improvement. For example, the comprehensive technical efficiency of pig farming is 0.893, which indicates that there is a waste of 10.7% of the breeding resources in pig farming production. In general, the comprehensive technical efficiency of farmers is mostly higher than 0.9, and there is room for improvement.

In terms of the pigs’ welfare level, the comprehensive efficiency of the farmers with the optimal condition for pig welfare is 0.983, and the average efficiencies of the poor, medium, and good farmers are 0.345, 0.568, and 0.626, respectively. There is confirmation that the different welfare levels of the farmers also have different efficiencies. The efficiency of agriculture farming also has a certain impact. Additionally, the pig welfare level has a specific impact on the pig production efficiency of the farmers.

By adopting forced enter and backward elimination methods of DEA-Tobit model, the whole model and simplest model were obtained. Compare with the whole model results, the simplest model has lower AIC value, which means the simplest model has better explanation. As can be seen in Table 8, there are a significant positive relationship between gender, feeding form and comprehensive technical efficiency. Pig breeding years, farming scale and science and technology personnel have negative relation with comprehensive technical efficiency.

The gender of the pig farmer has an impact on comprehensive technical efficiency. In terms of pig breeding, each increase in male farmers results in an efficiency increase of 0.383%.

When the farmers’ farming life is longer, the farmers’ pig production efficiency is higher. In the family characteristics of the variables, the farmers’ number of pig breeding years have a certain impact on the production efficiency of pigs and results in a significance degree of 1%. When the farmers’ number of pig breeding years is higher, the pig farming experience is fuller and the farmers tend to be more concerned regarding the country's policies, which may result in higher production efficiency.

The farming scale is negatively related with comprehensive technical efficiency and results in a significance degree of 1%, which means farmers with larger scale do not have more advantages of scale effect than small to medium scale farmers. Not opened feeding form has higher comprehensive technical efficiency than opened feeding form. The science and technology personnel index has negative relation with comprehensive technical efficiency, implying that they have not play an important role in improving.

The pig welfare level of pig breeding farmers has a substantial impact on efficiency, both overall and for the individual factors. The comprehensive situation of pig welfare has a significant effect on the pig production efficiency of farmers with a significance degree of 1%. This finding shows that when the evaluation of pig welfare is better, the efficiency of pig breeding is higher. If the welfare level increases by one unit, agriculture efficiency will increase by 0.163 units, which better maximizes the benefits of farmers.

## Conclusions and policy implications

Based on the survey data from 773 pig farmers in 23 counties in the Hunan, Zhejiang, Guangdong, Guizhou, and Shanxi Provinces, this paper conducted a comprehensive evaluation of the pig welfare levels of pig farmers by using the Delphi method, AHP, and DEA-Tobit regression model to analyze pig farmers’ pig production efficiency and influencing factors. The main conclusions are as follows. First, the pig welfare levels for most of the farmers are at middle, lower, and poor levels. Second, the comprehensive technical efficiency of farmers is in a good level. This is because of the low feeding cost and intensive feeding of small-medium farmers. Large scale farmers valued R&D investment, disease prevention and risk control, which has led to a higher level of comprehensive technical efficiency as well. Third, in the analysis of factors that affect the efficiency of pig breeding, the basic characteristics of individual farmers, family characteristics, the characteristics of the welfare level of the pigs, and the comprehensive welfare level of the pigs have an impact on pig production efficiency, especially pig welfare levels. The characteristic variables are significant at the 5%level. When the scale of farming is larger, the levels of agricultural, science, and technology personnel are more adequate, and the farmers’ pig breeding technology is more scientific, which results in higher pig production efficiency. In addition, the overall welfare level of pigs also has a significant impact on the efficiency of pig farming. The farmers who treat their pigs well achieve higher efficiency in agriculture.

Therefore, the government should build a safety supervision system to strengthen the quality of live pig production, regulate breeding behavior, and force farmers to choose healthy farming methods to improve the level of pig welfare and the quality of pig meat. This strategy will, in turn, improve consumer demand and confidence. In addition, the government should make full use of a variety of methods to communicate with farmers, such as television, radio, the Internet, and other media, to improve the understanding of pig welfare and to create a healthy environment for the development of pig breeding. The government should regularly strengthen the breeding technology training of farmers and promote the awareness of epidemic prevention to form good pig breeding habits. It is important for the government to increase the policies that support farmers’ agriculture pig production to promote agriculture industry development. Finally, farmers should also actively learn new farming techniques and increase their own knowledge to improve the level of pig farming.

## Supporting information

S1 File Weight analysis results. (PDF)

S2 File 773 farmers pig welfare level calculation index. (PDF)

S3 File Average input and output of the 773 pig farmers every year for each pig. (PDF)

S4 File Variables explained and statistical description. (PDF)

S5 File Production efficiency results of the 773 pig farmers. (PDF)
