# Supplementary material for: Empirical analysis of pig welfare levels and their impact on pig breeding efficiency—Based on 773 pig farmers’ survey data
Source: PLoS One. 2017 Dec 27;12(12):e0190108. doi: 10.1371/journal.pone.0190108 (PMC5744959; doi:10.1371/journal.pone.0190108)
Supplement: S1 File — (PDF) [file pone.0190108.s001.pdf]

S1: Weight analysis results

Expert No.	B1B2	B1B3	B1B4	B1B5	B2B1	B2B3	B2B4	B2B5	B3B1	B3B2	B3B4	B3B5	B4B1	B4B2	B4B3	B4B5	B5B1	B5B2	B5B3	B5B4
1	1	1/3	2	3	1	1	2	3	3	1	3	4	1/2	1/2	1/3	1/3	1/3	1/2	1/4	3
2	1	1/3	2	3	1	1	2	3	3	1	3	3	1/2	1/2	1/3	1/3	1/3	1/3	1/3	3
3	1	1/3	2	3	1	1	2	3	3	1	3	4	1/2	1/2	1/3	1/3	1/3	1/3	1/4	3
4	1	1/3	2	3	1	1	2	2	3	1	3	3	1/2	1/2	1/3	1/2	1/3	1/2	1/3	2
5	1	1/3	2	3	1	1	2	2	3	1	3	4	1/2	1/2	1/3	1/2	1/3	1/2	1/3	2
6	1	1/3	2	3	2	1	2	2	3	1	3	4	1/2	1/2	1/3	1/3	1/3	1/2	1/4	3
7	3	1/3	1	3	1/3	1	2	2	3	1	3	3	1	1/2	1/3	1/4	1/3	1/2	1/3	4
8	3	1/3	1	3	1/2	1	2	2	3	1	3	3	1	1/2	1/3	1/5	1/3	1/2	1/3	5
9	3	1/3	2	3	1/2	1	3	2	3	1	3	4	1/2	1/3	1/3	1/3	1/3	1/2	1/4	3
10	2	1/3	2	3	1/2	1/2	3	3	3	2	3	3	1/2	1/3	1/3	1/3	1/3	1/2	1/3	3
11	2	1/3	2	3	1	1	1	2	3	1	2	4	1/2	1	1/2	1/3	1/3	1/2	1/4	3
12	1	1/3	2	3	1	1	1	2	3	1	2	3	1/2	1	1/2	1/3	1/3	1/2	1/3	3
13	1	1/3	2	3	1/2	1	1	2	3	1	2	3	1/2	1	1/2	1/3	1/3	1/2	1/3	3
14	1	1/3	3	3	1	1	1	2	3	1	3	2	1/3	1	1/3	1/3	1/3	1/2	1/2	3
15	1	1/3	3	3	1	1	1/2	3	3	1	3	3	1/3	2	1/3	1/3	1/3	1/3	1	3
16	1	1/3	2	3	2	1	2	3	2	1	3	1	1/2	1/2	1/3	1/3	1/3	1/3	1/3	3
17	1	1/2	2	3	1	1	2	2	1	1	3	3	1/2	1/2	1/3	1/3	1/3	1/2	1/3	3
18	1	1	2	1	1	1	2	2	1	1	3	3	1/2	1/2	1/3	1/3	1	1/2	1/3	3
19	1	1	2	2	2	1	2	2	1	1	3	2	1/2	1/2	1/3	1/3	1/2	1/2	1/2	3
20	1/2	1	2	2	2	1	2	2	1	1	3	3	1/2	1/2	1/3	1/3	1/2	1/2	1/3	3

21	1/3	1	2	3	2	2	1	2	1	1/2	3	3	1/2	2	1/3	1/3	1/3	1/2	1/3	3
22	1/2	1/2	1	3	2	2	2	2	2	1/2	3	2	1	1/2	1/3	1/3	1/3	1/2	1/2	3
23	1/3	1/3	3	3	3	2	1	2	3	1/2	3	2	1/3	2	1/3	1/3	1/3	1/2	1/2	3
24	1	1/3	2	3	1	3	2	2	3	1/3	3	3	1/2	1/2	1/3	1/2	1/3	1/2	1/3	3
25	1	1/3	2	3	1	1	2	2	3	1	3	3	1/2	1/2	1/3	1/2	1/3	1/2	1/3	3
26	1	1/3	2	3	1	2	2	2	3	1/2	3	3	1/2	1/2	1/3	1	1/3	1/2	1/3	3
27	1	1/3	2	3	1	1	2	2	3	1	3	3	1/2	1/2	1/3	1/3	1/3	1/2	1/3	3
28	1	1/3	2	3	1	1	2	2	3	1	3	3	1/2	1/2	1/3	1/3	1/3	1/2	1/3	3
29	1	1/3	2	3	1	1	2	2	3	1	3	3	1/2	1/2	1/3	1/3	1/3	1/2	1/3	3
30	1	1/3	2	3	1	1	2	2	3	1	3	3	1/2	1/2	1/3	1/3	1/3	1/2	1/3	3
31	1	1/3	2	2	1	1	2	2	3	1	3	3	1/2	1/2	1/3	1/3	1/2	1/2	1/3	3
32	1	1/3	2	3	1	1	2	2	3	1	3	3	1/2	1/2	1/3	1/3	1/3	1/2	1/3	3
33	1	1/3	2	2	1	1	2	2	3	1	3	3	1/2	1/2	1/3	1/3	1/3	1/2	1/3	3
34	1	1/3	2	3	1	1	2	2	3	1	3	3	1/2	1/2	1/3	1/3	1/3	1/2	1/3	3
35	1	1/3	2	3	1	1	2	2	3	1	3	3	1/2	1/2	1/3	1/3	1/2	1/2	1/3	3
36	1	1	2	3	1	1	2	2	1	1	3	3	1/2	1/2	1/3	1/3	1/3	1/2	1/3	3
37	1	1	2	2	1	1	2	2	1	1	3	3	1/2	1/2	1/3	1/3	1/2	1/2	1/3	3
38	1	2	2	3	1	1	2	2	1/2	1	3	3	1/2	1/2	1/3	1/3	1/3	1/2	1/3	3
39	1	1/2	2	3	1	1	2	2	2	1	3	3	1/2	1/2	1/3	1/3	1/3	1/2	1/3	3
40	1	1/2	2	1	1	1	2	2	2	1	3	3	1/2	1/2	1/3	1/3	1	1/2	1/3	3
41	2	1/2	2	4	1	1	2	2	2	1	3	3	1/2	1/2	1/3	1/3	1/4	1/2	1/3	3
42	1	1/3	2	4	1	2	2	2	3	1	3	3	1/2	1/2	1/3	1/3	1/4	1/2	1/3	3
43	1	1/3	2	4	1	2	2	2	3	1	3	3	1/2	1/2	1/3	1/3	1/4	1/2	1/3	3
44	1	1/3	2	3	1	2	2	2	3	1	2	3	1/2	1/2	1/3	1/3	1/3	1/2	1/3	3
45	1	1/2	1	3	1	2	2	2	3	1	3	3	1	1/2	1/3	1/3	1/3	1/2	1/3	3

46	3	1/3	1	2	1/2	1	2	2	3	2	3	4	1	1/2	1/3	1/3	1/2	1/2	1/4	3
47	1	1	1	2	1	2	2	2	1	1	3	4	1	1/2	1/3	1/3	1/2	1/2	1/4	3
48	1	1	3	3	1	1	2	2	1	1	2	4	1/3	1/2	1/3	1/3	1/3	1/2	1/4	3
49	1	1/3	3	3	1	1	2	2	3	1	3	3	1/3	1/2	1/3	1/3	1/3	1/2	1/3	3
50	1	1/3	2	3	1	1	2	2	3	1	4	3	1/2	1/2	1/4	1/3	1/3	1/2	1/3	3
51	1	1/3	2	3	1	1	2	2	3	1	3	3	1/2	1/2	1/3	1/3	1/3	1/2	1/3	3
52	1	1/3	2	3	1	1	2	2	3	1	3	3	1/2	1/2	1/3	1/3	1/3	1/2	1/3	3
53	1	1/3	2	2	1	1	2	2	3	1	3	3	1/2	1/2	1/3	1/3	1/2	1/2	1/3	3
