# Supplementary material for: Empirical analysis of pig welfare levels and their impact on pig breeding efficiency—Based on 773 pig farmers’ survey data
Source: PLoS One. 2017 Dec 27;12(12):e0190108. doi: 10.1371/journal.pone.0190108 (PMC5744959; doi:10.1371/journal.pone.0190108)
Supplement: S2 File — (PDF) [file pone.0190108.s002.pdf]

S2: 773 farmers pig welfare level calculation index

Sample No.	B1（21%）			B2（23%）			B3（34%）				B4（9%）		B5（13%）		Total score	Scale	Level
	B1 score	C1	C2	B2 score	C3	C4	B3 score	C5	C6	C7	B4 score	C8	B5 score	C9			
1	12.60	55	65	17.83	80	75	13.60	65	0	55	0.00	0	7.80	60	51.83	42	1
2	13.65	60	70	17.25	75	75	13.03	65	0	50	0.00	0	7.80	60	51.73	75	1
3	14.70	70	70	18.40	85	75	17.00	75	0	75	2.25	25	7.80	60	60.15	78	2
4	12.60	55	65	17.83	80	75	12.47	65	0	45	0.00	0	7.80	60	50.69	89	1
5	12.60	55	65	18.40	85	75	13.60	65	0	55	0.00	0	7.80	60	52.40	91	1
6	14.70	70	70	15.53	65	70	19.83	80	25	70	4.50	50	8.45	65	63.01	334	2
7	14.70	70	70	15.53	70	65	22.67	80	50	70	4.50	50	8.45	65	65.84	446	2
8	14.70	70	70	14.95	65	65	20.97	85	25	75	4.50	50	9.10	70	64.22	532	2
9	14.70	70	70	15.53	65	70	20.40	80	25	75	4.50	50	8.45	65	63.58	641	2
10	15.75	75	75	16.68	70	75	19.83	80	25	70	6.75	75	8.45	65	67.46	783	2
11	15.75	75	75	17.25	75	75	23.80	85	50	75	4.50	50	8.45	65	69.75	806	2
12	15.75	75	75	16.68	75	70	26.63	80	75	80	6.75	75	9.75	75	75.56	1269	3
13	18.38	85	90	17.83	75	80	27.77	85	75	85	6.75	75	9.75	75	80.47	1341	3
14	16.80	80	80	14.38	60	65	26.07	85	75	70	6.75	75	9.75	75	73.74	1555	2
15	16.80	80	80	14.95	65	65	26.07	85	75	70	4.50	50	9.75	75	72.07	1603	2
16	13.65	65	65	14.38	60	65	26.07	75	75	80	6.75	75	9.10	70	69.94	1776	2
17	17.33	80	85	16.68	70	75	26.63	80	75	80	6.75	75	10.40	80	77.78	1897	3
18	17.85	85	85	18.40	75	85	29.47	90	80	90	9.00	100	11.05	85	85.77	2367	4
19	11.55	55	55	17.83	80	75	12.47	65	0	45	2.25	25	7.80	60	51.89	38	1
20	13.65	60	70	17.83	80	75	11.90	50	0	55	0.00	0	8.45	65	51.83	67	1
21	15.23	70	75	14.38	60	65	25.50	85	75	65	0.00	0	9.10	70	64.20	75	2

22	13.65	60	70	17.25	75	75	12.47	60	0	50	0.00	0	7.80	60	51.17	82	1
23	13.65	60	70	17.25	75	75	16.43	65	25	55	0.00	0	7.80	60	55.13	128	1
24	14.18	65	70	17.25	75	75	17.57	70	25	60	2.25	25	8.45	65	59.69	245	1
25	16.80	75	85	14.95	65	65	20.40	80	25	75	4.50	50	9.10	70	65.75	467	2
26	14.18	55	80	15.53	70	65	24.37	65	75	75	4.50	50	9.75	75	68.32	523	2
27	12.08	50	65	14.38	60	65	24.37	65	75	75	4.50	50	9.75	75	65.07	655	2
28	14.18	70	65	14.95	65	65	20.40	80	25	75	4.50	50	9.75	75	63.78	860	2
29	11.55	45	65	13.23	50	65	20.97	80	25	80	6.75	75	9.10	70	61.59	1120	2
30	12.60	65	55	12.65	45	65	25.50	70	75	80	6.75	75	9.75	75	67.25	1185	2
31	13.13	65	60	12.65	45	65	24.93	70	75	75	0.00	0	10.40	80	61.11	1240	2
32	16.28	75	80	16.68	70	75	25.50	80	70	75	6.75	75	10.40	80	75.60	1420	3
33	16.80	85	75	16.10	65	75	26.63	80	75	80	6.75	75	11.05	85	77.33	1450	3
34	12.08	60	55	17.83	75	80	13.03	65	0	50	0.00	0	7.80	60	50.73	35	1
35	14.18	60	75	17.83	75	80	13.03	65	0	50	0.00	0	7.80	60	52.83	40	1
36	13.65	60	70	17.83	75	80	13.03	65	0	50	0.00	0	7.80	60	52.31	43	1
37	13.65	60	70	17.25	75	75	13.03	65	0	50	0.00	0	7.80	60	51.73	51	1
38	12.60	60	60	17.25	75	75	12.47	60	0	50	0.00	0	7.80	60	50.12	56	1
39	15.23	65	80	15.53	70	65	25.50	75	75	75	0.00	0	9.10	70	65.35	68	2
40	13.65	60	70	17.25	75	75	13.03	65	0	50	0.00	0	7.80	60	51.73	70	1
41	13.65	60	70	17.25	75	75	13.03	65	0	50	0.00	0	7.80	60	51.73	76	1
42	13.65	60	70	17.25	75	75	13.03	65	0	50	2.25	25	7.80	60	53.98	85	1
43	14.18	60	75	17.25	75	75	13.60	65	0	55	0.00	0	7.80	60	52.83	110	1
44	14.18	65	70	17.25	75	75	16.43	65	25	55	0.00	0	8.45	65	56.31	325	1
45	15.23	70	75	14.38	60	65	26.63	85	75	75	4.50	50	9.75	75	70.48	480	2
46	14.18	55	80	14.38	60	65	19.27	70	25	75	4.50	50	9.75	75	62.07	550	2

47	13.65	60	70	14.38	60	65	20.40	80	25	75	4.50	50	9.75	75	62.68	620	2
48	14.18	65	70	13.23	50	65	19.83	75	25	75	4.50	50	10.40	80	62.13	890	2
49	17.33	80	85	19.55	85	85	28.90	85	80	90	9.00	100	11.05	85	85.83	4200	4
50	17.85	80	90	19.55	85	85	29.47	90	80	90	9.00	100	11.05	85	86.92	6600	4
51	13.13	55	70	16.68	80	65	11.90	60	0	45	0.00	0	8.45	65	50.15	45	1
52	13.13	60	65	16.10	75	65	13.60	65	0	55	0.00	0	7.80	60	50.63	48	1
53	12.60	55	65	17.25	75	75	13.03	60	0	55	0.00	0	7.80	60	50.68	55	1
54	13.13	60	65	17.25	75	75	14.17	65	0	60	0.00	0	7.15	55	51.69	56	1
55	13.13	60	65	17.25	75	75	13.60	65	0	55	0.00	0	7.80	60	51.78	69	1
56	12.08	55	60	17.25	75	75	13.03	65	0	50	0.00	0	8.45	65	50.81	78	1
57	16.28	85	70	13.80	55	65	20.97	85	25	75	0.00	0	9.75	75	60.79	79	2
58	13.65	65	65	17.25	75	75	12.47	65	0	45	2.25	25	7.80	60	53.42	86	1
59	14.70	70	70	14.95	65	65	18.70	75	25	65	2.25	25	8.45	65	59.05	310	1
60	13.13	60	65	14.38	60	65	26.07	80	75	75	4.50	50	9.10	70	67.17	460	2
61	14.70	75	65	14.95	65	65	26.07	80	75	75	4.50	50	9.75	75	69.97	540	2
62	12.08	65	50	13.80	55	65	20.97	85	25	75	4.50	50	9.75	75	61.09	680	2
63	11.55	55	55	13.23	50	65	27.20	85	75	80	4.50	50	9.75	75	66.23	830	2
64	17.33	80	85	18.98	80	85	29.47	90	80	90	9.00	100	10.40	80	85.17	5500	4
65	12.60	65	55	17.83	75	80	13.03	65	0	50	0.00	0	7.80	60	51.26	45	1
66	13.65	60	70	17.25	75	75	13.60	65	0	55	0.00	0	8.45	65	52.95	55	1
67	13.65	60	70	18.40	80	80	12.47	60	0	50	2.25	25	7.80	60	54.57	58	1
68	13.13	60	65	15.53	75	60	14.17	65	0	60	0.00	0	7.80	60	50.62	64	1
69	12.60	60	60	16.10	75	65	13.03	65	0	50	0.00	0	7.80	60	49.53	66	1
70	13.65	60	70	17.25	75	75	13.03	65	0	50	0.00	0	7.80	60	51.73	72	1
71	14.70	75	65	16.10	75	65	13.03	65	0	50	2.25	25	9.10	70	55.18	78	1

72	13.13	60	65	16.10	75	65	13.03	65	0	50	0.00	0	7.80	60	50.06	80	1
73	16.28	70	85	16.68	70	75	23.80	85	50	75	0.00	0	9.75	75	66.50	93	2
74	12.08	50	65	17.83	80	75	12.47	65	0	45	0.00	0	7.80	60	50.17	206	1
75	13.65	60	70	14.38	60	65	21.53	65	50	75	4.50	50	9.75	75	63.81	334	2
76	15.23	75	70	14.38	60	65	23.80	85	50	75	4.50	50	9.75	75	67.65	455	2
77	14.18	65	70	14.38	60	65	23.23	80	50	75	4.50	50	9.75	75	66.03	582	2
78	14.70	70	70	14.95	65	65	26.63	85	75	75	4.50	50	9.75	75	70.53	627	2
79	14.18	65	70	13.80	55	65	26.07	75	75	80	4.50	50	9.75	75	68.29	768	2
80	14.70	65	75	13.23	50	65	27.20	85	75	80	4.50	50	9.75	75	69.38	850	2
81	18.38	90	85	20.13	90	85	29.47	90	80	90	9.00	100	11.05	85	88.02	7500	4
82	13.65	65	65	16.68	70	75	11.90	60	0	45	0.00	0	8.45	65	50.68	38	1
83	13.13	60	65	16.10	70	70	13.03	60	0	55	0.00	0	7.80	60	50.06	48	1
84	14.18	65	70	17.25	75	75	12.47	65	0	45	0.00	0	8.45	65	52.34	60	1
85	12.60	55	65	17.83	85	70	13.60	65	0	55	2.25	25	7.80	60	54.08	80	1
86	12.08	55	60	16.68	75	70	13.03	65	0	50	0.00	0	9.10	70	50.88	85	1
87	11.55	55	55	18.40	85	75	12.47	65	0	45	0.00	0	9.10	70	51.52	85	1
88	13.65	60	70	16.68	70	75	13.60	65	0	55	2.25	25	8.45	65	54.63	91	1
89	14.18	70	65	13.23	55	60	16.43	75	0	70	0.00	0	9.10	70	52.93	220	1
90	14.70	70	70	12.08	50	55	19.83	75	25	75	2.25	25	8.45	65	57.31	240	1
91	13.13	60	65	14.38	60	65	24.93	70	75	75	4.50	50	9.75	75	66.68	320	2
92	14.70	80	60	14.38	60	65	26.63	85	75	75	4.50	50	9.75	75	69.96	470	2
93	11.55	55	55	14.38	60	65	20.97	85	25	75	4.50	50	9.75	75	61.14	530	2
94	11.03	50	55	12.65	45	65	23.80	80	50	80	4.50	50	9.75	75	61.73	860	2
95	15.75	70	80	17.25	75	75	26.63	80	75	80	4.50	50	11.05	85	75.18	1350	3
96	16.80	85	75	16.68	70	75	26.63	80	75	80	4.50	50	11.05	85	75.66	1470	3

97	16.28	80	75	16.68	70	75	26.63	80	75	80	4.50	50	11.05	85	75.13	1620	3
98	17.33	85	80	16.10	65	75	26.63	80	75	80	6.75	75	11.05	85	77.86	1850	3
99	12.60	60	60	13.80	55	65	13.60	65	0	55	0.00	0	8.45	65	48.45	40	1
100	14.70	65	75	14.38	60	65	13.60	65	0	55	0.00	0	7.80	60	50.48	55	1
101	11.55	45	65	15.53	70	65	13.60	65	0	55	0.00	0	8.45	65	49.13	75	1
102	14.70	65	75	13.80	50	70	13.60	65	0	55	0.00	0	8.45	65	50.55	160	1
103	13.13	60	65	15.53	70	65	20.97	85	25	75	4.50	50	9.10	70	63.22	280	2
104	15.23	70	75	14.38	60	65	20.97	85	25	75	4.50	50	9.75	75	64.82	340	2
105	11.55	65	45	14.95	65	65	20.40	80	25	75	4.50	50	9.10	70	60.50	460	2
106	12.60	65	55	13.80	55	65	14.17	70	0	55	0.00	0	8.45	65	49.02	530	1
107	13.13	75	50	14.38	60	65	20.97	85	25	75	4.50	50	9.10	70	62.07	620	2
108	15.23	85	60	14.38	60	65	20.97	85	25	75	4.50	50	9.10	70	64.17	750	2
109	11.03	50	55	14.38	60	65	24.93	65	75	80	4.50	50	9.75	75	64.58	1050	2
110	11.55	70	40	12.65	45	65	27.20	85	75	80	4.50	50	9.75	75	65.65	1100	2
111	15.75	75	75	13.23	50	65	27.20	85	75	80	4.50	50	9.75	75	70.43	1400	2
112	13.65	55	75	12.08	40	65	21.53	85	25	80	4.50	50	9.75	75	61.51	1700	2
113	13.13	60	65	12.08	40	65	27.20	85	75	80	4.50	50	9.75	75	66.65	1750	2
114	17.33	80	85	16.68	70	75	26.63	80	75	80	4.50	50	10.40	80	75.53	1820	3
115	17.33	85	80	16.10	55	85	25.50	80	75	70	9.00	100	10.40	80	78.33	2100	3
116	18.90	95	85	18.40	75	85	33.43	100	95	100	9.00	100	12.35	95	92.08	32000	4
117	11.55	50	60	14.38	65	60	10.77	65	0	30	0.00	0	7.15	55	43.84	35	1
118	13.13	65	60	14.38	60	65	11.33	65	0	35	2.25	25	7.15	55	48.23	38	1
119	11.55	50	60	13.23	50	65	12.47	55	0	55	0.00	0	8.45	65	45.69	50	1
120	14.70	65	75	14.38	60	65	13.03	60	0	55	0.00	0	8.45	65	50.56	62	1
121	16.80	80	80	14.38	60	65	23.23	85	75	45	0.00	0	9.10	70	63.51	68	2

122	14.70	70	70	13.23	60	55	13.60	65	0	55	0.00	0	9.10	70	50.63	75	1
123	11.03	55	50	14.38	60	65	13.03	70	0	45	0.00	0	7.80	60	46.23	85	1
124	14.18	65	70	15.53	70	65	18.70	75	25	65	2.25	25	8.45	65	59.10	245	1
125	15.23	70	75	13.80	55	65	23.80	85	75	50	4.50	50	9.10	70	66.43	352	2
126	15.23	80	65	15.53	70	65	25.50	80	75	70	4.50	50	9.10	70	69.85	468	2
127	13.13	65	60	13.80	55	65	25.50	75	75	75	4.50	50	9.10	70	66.03	577	2
128	11.55	55	55	15.53	70	65	20.97	85	25	75	4.50	50	9.10	70	61.64	693	2
129	12.08	60	55	14.38	60	65	20.40	80	25	75	4.50	50	9.75	75	61.10	707	2
130	17.85	85	85	16.68	70	75	26.63	80	75	80	4.50	50	10.40	80	76.06	736	3
131	13.65	65	65	14.38	60	65	20.97	85	25	75	4.50	50	9.75	75	63.24	752	2
132	14.70	85	55	13.23	50	65	18.13	80	25	55	4.50	50	9.75	75	60.31	780	2
133	17.85	80	90	17.25	75	75	26.07	80	75	75	4.50	50	10.40	80	76.07	860	3
134	17.33	85	80	19.55	85	85	29.47	90	80	90	9.00	100	11.05	85	86.39	3750	4
135	10.50	55	45	15.53	70	65	10.77	65	0	30	0.00	0	8.45	65	45.24	32	1
136	9.98	50	45	15.53	70	65	14.73	65	0	65	0.00	0	8.45	65	48.68	38	1
137	13.13	55	70	15.53	70	65	13.03	65	0	50	0.00	0	7.80	60	49.48	46	1
138	12.08	60	55	15.53	70	65	11.90	65	0	40	0.00	0	9.10	70	48.60	47	1
139	9.98	45	50	15.53	70	65	14.17	65	0	60	0.00	0	9.10	70	48.77	60	1
140	12.08	65	50	15.53	70	65	13.03	65	0	50	0.00	0	9.75	75	50.38	65	1
141	13.13	75	50	14.38	60	65	24.93	85	75	60	4.50	50	8.45	65	65.38	83	2
142	13.65	70	60	14.38	60	65	12.47	65	0	45	2.25	25	8.45	65	51.19	180	1
143	12.60	55	65	14.95	65	65	10.77	65	0	30	2.25	25	7.80	60	48.37	240	1
144	11.03	60	45	13.80	55	65	11.33	65	0	35	2.25	25	8.45	65	46.86	260	1
145	14.70	70	70	14.38	60	65	26.07	80	75	75	4.50	50	9.10	70	68.74	370	2
146	12.60	55	65	13.80	55	65	26.63	85	75	75	4.50	50	9.10	70	66.63	450	2

147	16.80	75	85	17.25	75	75	26.63	80	75	80	9.00	100	10.40	80	80.08	780	3
148	15.75	80	70	16.10	80	60	26.63	80	75	80	9.00	100	10.40	80	77.88	1150	3
149	15.23	75	70	14.95	70	60	27.20	85	75	80	9.00	100	10.40	80	76.78	1250	3
150	17.85	85	85	17.83	75	80	26.63	80	75	80	9.00	100	10.40	80	81.71	1850	3
151	16.80	80	80	16.10	60	80	27.20	85	75	80	9.00	100	10.40	80	79.50	2100	3
152	17.33	85	80	16.68	60	85	26.63	80	75	80	9.00	100	9.75	75	79.38	2250	3
153	12.60	65	55	15.53	70	65	14.17	65	0	60	0.00	0	7.15	55	49.44	50	1
154	14.18	60	75	15.53	70	65	12.47	65	0	45	0.00	0	9.10	70	51.27	55	1
155	14.18	70	65	14.95	65	65	22.67	60	75	65	0.00	0	8.45	65	60.24	65	2
156	12.60	60	60	16.10	65	75	15.30	70	0	65	0.00	0	8.45	65	52.45	78	1
157	12.08	50	65	15.53	70	65	14.73	65	0	65	2.25	25	7.80	60	52.38	120	1
158	10.50	45	55	14.95	65	65	14.73	65	0	65	2.25	25	8.45	65	50.88	125	1
159	12.08	70	45	15.53	70	65	13.03	65	0	50	2.25	25	8.45	65	51.33	135	1
160	13.65	75	55	14.38	60	65	11.33	65	0	35	2.25	25	7.80	60	49.41	140	1
161	13.13	60	65	14.95	65	65	10.77	65	0	30	2.25	25	8.45	65	49.54	160	1
162	14.70	75	65	15.53	70	65	22.67	65	75	60	4.50	50	7.80	60	65.19	290	2
163	13.65	65	65	13.23	50	65	26.63	85	75	75	4.50	50	9.75	75	67.76	1300	2
164	14.70	75	65	12.65	45	65	23.80	85	50	75	4.50	50	9.75	75	65.40	1450	2
165	14.70	75	65	12.08	40	65	23.80	85	50	75	4.50	50	9.75	75	64.83	1480	2
166	16.80	80	80	16.68	70	75	26.63	80	75	80	9.00	100	9.75	75	78.86	1520	3
167	14.70	75	65	16.68	70	75	27.20	85	75	80	6.75	75	9.75	75	75.08	1680	3
168	16.80	75	85	15.53	60	75	27.77	90	75	80	9.00	100	9.75	75	78.84	2200	3
169	12.08	60	55	17.83	75	80	13.60	70	0	50	0.00	0	7.80	60	51.30	32	1
170	15.23	70	75	13.80	55	65	22.67	75	75	50	0.00	0	8.45	65	60.14	40	2
171	12.08	55	60	17.83	75	80	14.73	65	0	65	0.00	0	8.45	65	53.08	45	1

172	13.65	60	70	17.83	75	80	14.17	65	0	60	0.00	0	8.45	65	54.09	51	1
173	12.08	50	65	17.25	70	80	14.17	65	0	60	0.00	0	7.80	60	51.29	55	1
174	11.55	50	60	17.83	75	80	15.30	65	0	70	0.00	0	9.10	70	53.78	72	1
175	13.13	65	60	14.95	65	65	21.53	75	50	65	2.25	25	8.45	65	60.31	85	2
176	14.18	65	70	15.53	70	65	13.60	65	0	55	2.25	25	9.10	70	54.65	253	1
177	12.60	65	55	13.23	50	65	24.37	85	75	55	4.50	50	9.75	75	64.44	327	2
178	15.75	65	85	15.53	70	65	24.37	85	75	55	4.50	50	9.10	70	69.24	336	2
179	14.18	75	60	14.38	60	65	24.37	80	75	60	4.50	50	9.10	70	66.52	345	2
180	16.80	85	75	14.95	65	65	24.37	80	75	60	4.50	50	9.10	70	69.72	405	2
181	15.23	70	75	13.80	55	65	19.27	85	25	60	4.50	50	9.10	70	61.89	425	2
182	13.13	60	65	13.80	55	65	26.07	85	75	70	4.50	50	9.10	70	66.59	580	2
183	16.80	80	80	16.68	70	75	24.93	70	75	75	9.00	100	9.75	75	77.16	610	3
184	12.08	55	60	14.38	60	65	22.10	75	50	70	4.50	50	9.10	70	62.15	735	2
185	12.60	65	55	13.23	50	65	23.80	85	50	75	4.50	50	9.10	70	63.23	780	2
186	17.33	80	85	18.98	80	85	29.47	90	80	90	9.00	100	11.05	85	85.82	3500	4
187	15.23	70	75	17.25	80	70	14.17	65	0	60	0.00	0	7.80	60	54.44	38	1
188	16.28	80	75	13.80	55	65	18.70	85	25	55	2.25	25	9.10	70	60.13	40	2
189	10.50	45	55	16.68	80	65	15.30	70	0	65	0.00	0	9.10	70	51.58	45	1
190	14.70	65	75	13.80	55	65	24.37	85	75	55	0.00	0	8.45	65	61.32	56	2
191	9.45	45	45	16.10	70	70	12.47	65	0	45	0.00	0	8.45	65	46.47	62	1
192	11.55	50	60	17.25	80	70	11.90	70	0	35	0.00	0	7.15	55	47.85	68	1
193	13.13	60	65	15.53	65	70	10.77	65	0	30	0.00	0	8.45	65	47.87	74	1
194	14.70	65	75	14.38	60	65	22.10	65	75	55	0.00	0	9.10	70	60.28	81	2
195	13.65	70	60	17.25	75	75	13.60	65	0	55	0.00	0	7.80	60	52.30	85	1
196	11.03	45	60	14.38	60	65	18.70	75	25	65	2.25	25	8.45	65	54.80	355	1

197	16.80	85	75	14.38	60	65	24.93	85	75	60	4.50	50	9.75	75	70.36	450	2
198	16.28	80	75	13.80	55	65	25.50	85	75	65	4.50	50	9.75	75	69.83	460	2
199	13.65	65	65	15.53	70	65	22.67	85	50	65	4.50	50	9.75	75	66.09	556	2
200	13.65	50	80	16.10	75	65	25.50	80	75	70	4.50	50	9.75	75	69.50	580	2
201	13.13	60	65	13.80	55	65	23.23	85	50	70	4.50	50	9.75	75	64.41	665	2
202	13.65	65	65	14.38	60	65	24.37	75	75	65	4.50	50	9.75	75	66.64	775	2
203	16.28	75	80	12.65	45	65	25.50	85	75	65	4.50	50	9.75	75	68.68	880	2
204	16.80	85	75	12.65	45	65	27.77	85	75	85	6.75	75	10.40	80	74.37	3100	2
205	12.08	55	60	17.25	80	70	12.47	65	0	45	0.00	0	9.10	70	50.89	41	1
206	12.08	60	55	16.10	70	70	13.60	65	0	55	0.00	0	8.45	65	50.23	45	1
207	13.65	60	70	15.53	65	70	14.17	70	0	55	0.00	0	7.80	60	51.14	58	1
208	14.18	65	70	17.25	80	70	12.47	65	0	45	0.00	0	8.45	65	52.34	65	1
209	12.60	55	65	14.95	60	70	12.47	65	0	45	0.00	0	7.80	60	47.82	80	1
210	13.13	70	55	14.38	55	70	14.17	65	0	60	0.00	0	7.80	60	49.47	81	1
211	15.23	80	65	13.80	55	65	23.80	85	75	50	0.00	0	9.10	70	61.93	85	2
212	15.75	75	75	14.95	65	65	23.80	85	75	50	0.00	0	9.10	70	63.60	88	2
213	15.75	65	85	14.38	60	65	21.53	85	50	55	2.25	25	9.75	75	63.66	224	2
214	11.55	65	45	13.23	50	65	14.73	65	0	65	2.25	25	8.45	65	50.21	268	1
215	13.65	65	65	14.38	60	65	19.83	65	50	60	4.50	50	9.75	75	62.11	385	2
216	11.03	55	50	13.80	55	65	21.53	75	50	65	4.50	50	9.75	75	60.61	412	2
217	13.65	75	55	13.80	55	65	22.67	85	50	65	4.50	50	9.75	75	64.37	533	2
218	16.28	80	75	15.53	70	65	22.67	80	50	70	4.50	50	9.75	75	68.72	628	2
219	12.08	70	45	14.95	65	65	23.23	85	50	70	4.50	50	9.75	75	64.51	780	2
220	17.85	80	90	15.53	70	65	25.50	70	75	80	9.00	100	9.75	75	77.63	820	3
221	16.80	85	75	17.25	75	75	26.63	90	75	70	9.00	100	9.75	75	79.43	1900	3

222	13.13	70	55	17.25	75	75	14.73	65	0	65	0.00	0	8.45	65	53.56	36	1
223	13.65	65	65	16.68	70	75	13.60	65	0	55	0.00	0	7.80	60	51.73	40	1
224	11.55	35	75	17.25	75	75	14.17	65	0	60	0.00	0	7.80	60	50.77	45	1
225	13.13	55	70	15.53	60	75	12.47	65	0	45	0.00	0	7.15	55	48.27	58	1
226	15.23	80	65	14.95	65	65	23.80	80	75	55	4.50	50	8.45	65	66.93	62	2
227	13.13	60	65	17.25	75	75	13.60	65	0	55	0.00	0	7.15	55	51.13	70	1
228	13.13	65	60	17.25	75	75	11.90	65	0	40	0.00	0	8.45	65	50.73	80	1
229	13.13	70	55	14.38	55	70	13.60	65	0	55	2.25	25	7.80	60	51.15	280	1
230	15.23	65	80	14.38	60	65	25.50	85	75	65	4.50	50	9.75	75	69.35	402	2
231	16.80	80	80	13.80	55	65	24.37	70	75	70	4.50	50	9.75	75	69.22	460	2
232	11.55	55	55	13.80	55	65	20.97	70	50	65	4.50	50	9.75	75	60.57	520	2
233	15.23	65	80	13.80	55	65	20.40	85	25	70	4.50	50	9.75	75	63.68	550	2
234	14.18	80	55	14.38	60	65	20.40	85	25	70	4.50	50	9.75	75	63.20	630	2
235	15.23	80	65	13.80	55	65	18.13	65	25	70	4.50	50	9.75	75	61.41	720	2
236	15.75	75	75	13.23	50	65	26.07	85	75	70	4.50	50	9.75	75	69.29	820	2
237	15.75	80	70	13.23	50	65	23.80	65	75	70	4.50	50	9.75	75	67.03	840	2
238	17.85	85	85	17.25	75	75	24.93	80	75	65	6.75	75	9.10	70	75.88	1850	3
239	11.55	65	45	17.25	75	75	14.73	65	0	65	0.00	0	8.45	65	51.98	36	1
240	14.18	65	70	17.25	75	75	14.73	65	0	65	0.00	0	8.45	65	54.61	41	1
241	14.18	60	75	15.53	60	75	13.60	65	0	55	0.00	0	8.45	65	51.75	48	1
242	12.60	60	60	16.10	65	75	15.30	65	0	70	0.00	0	7.80	60	51.80	53	1
243	11.55	55	55	16.10	65	75	14.17	65	0	60	0.00	0	7.80	60	49.62	62	1
244	12.60	65	55	17.25	75	75	13.60	65	0	55	0.00	0	7.80	60	51.25	73	1
245	12.60	60	60	15.53	60	75	13.60	65	0	55	0.00	0	9.10	70	50.83	85	1
246	10.50	55	45	15.53	65	70	14.17	65	0	60	0.00	0	8.45	65	48.64	87	1

247	16.28	80	75	13.80	55	65	21.53	60	75	55	0.00	0	9.10	70	60.71	91	2
248	14.70	75	65	14.38	60	65	22.10	60	75	60	2.25	25	9.75	75	63.18	310	2
249	16.28	75	80	14.38	60	65	22.67	85	50	65	4.50	50	9.75	75	67.57	435	2
250	16.80	80	80	14.95	65	65	22.67	85	50	65	4.50	50	9.75	75	68.67	568	2
251	15.23	80	65	14.95	65	65	22.67	85	50	65	4.50	50	9.75	75	67.09	654	2
252	16.28	70	85	13.80	55	65	22.67	85	50	65	4.50	50	9.75	75	66.99	708	2
253	15.75	65	85	13.23	50	65	24.93	80	75	65	4.50	50	9.75	75	68.16	788	2
254	17.33	80	85	16.68	70	75	24.93	65	75	80	9.00	100	9.10	70	77.03	872	3
255	18.38	85	90	18.40	75	85	28.90	85	80	90	9.00	100	11.05	85	85.73	4150	4
256	13.13	60	65	18.40	80	80	13.60	60	0	60	0.00	0	7.80	60	52.93	55	1
257	13.13	60	65	18.40	80	80	13.60	60	0	60	0.00	0	9.10	70	54.23	63	1
258	14.18	65	70	18.40	80	80	12.47	60	0	50	0.00	0	9.10	70	54.14	102	1
259	14.18	80	55	13.80	55	65	23.23	70	75	60	4.50	50	9.75	75	65.46	116	2
260	15.23	80	65	16.10	75	65	24.37	85	75	55	4.50	50	9.75	75	69.94	146	2
261	14.70	65	75	14.38	60	65	19.27	85	25	60	4.50	50	9.75	75	62.59	187	2
262	11.03	60	45	13.80	55	65	22.67	85	50	65	4.50	50	9.75	75	61.74	233	2
263	11.03	65	40	14.38	60	65	23.80	70	75	65	4.50	50	9.75	75	63.45	413	2
264	12.60	65	55	13.80	55	65	25.50	85	75	65	4.50	50	9.75	75	66.15	685	2
265	13.13	70	55	13.23	50	65	20.97	70	50	65	4.50	50	10.40	80	62.22	1032	2
266	16.28	70	85	13.23	50	65	24.37	70	75	70	4.50	50	9.10	70	67.47	1148	2
267	15.75	75	75	13.23	50	65	26.63	85	75	75	4.50	50	10.40	80	70.51	1241	2
268	16.80	85	75	17.25	75	75	26.63	85	75	75	6.75	75	10.40	80	77.83	1289	3
269	16.28	80	75	15.53	70	65	26.63	80	75	80	6.75	75	10.40	80	75.58	1350	3
270	16.80	85	75	16.68	70	75	26.07	80	75	75	6.75	75	10.40	80	76.69	1475	3
271	19.95	95	95	17.25	60	90	33.43	100	95	100	9.00	100	12.35	95	91.98	26000	4

272	18.90	95	85	16.68	60	85	33.43	100	95	100	9.00	100	12.35	95	90.36	28000	4
273	14.18	65	70	18.40	80	80	12.47	60	0	50	0.00	0	7.15	55	52.19	36	1
274	12.60	60	60	18.40	80	80	12.47	60	0	50	0.00	0	6.50	50	49.97	40	1
275	14.18	70	65	18.40	80	80	13.03	60	0	55	0.00	0	7.80	60	53.41	50	1
276	13.65	60	70	18.40	80	80	12.47	60	0	50	0.00	0	7.15	55	51.67	58	1
277	15.23	75	70	18.40	80	80	13.03	60	0	55	0.00	0	8.45	65	55.11	63	1
278	13.65	60	70	18.40	80	80	12.47	60	0	50	0.00	0	7.15	55	51.67	66	1
279	16.80	80	80	13.80	55	65	19.27	65	50	55	2.25	25	8.45	65	60.57	70	2
280	12.08	60	55	18.40	80	80	14.73	60	0	70	0.00	0	9.10	70	54.31	75	1
281	10.50	55	45	18.40	80	80	14.73	60	0	70	0.00	0	8.45	65	52.08	81	1
282	16.80	80	80	14.38	60	65	18.13	80	25	55	2.25	25	9.10	70	60.66	225	2
283	16.80	80	80	14.38	60	65	19.27	85	25	60	2.25	25	9.10	70	61.79	260	2
284	13.65	65	65	13.80	55	65	20.40	85	25	70	4.50	50	9.10	70	61.45	291	2
285	15.23	65	80	14.95	65	65	18.70	85	25	55	4.50	50	9.10	70	62.48	348	2
286	14.70	75	65	13.80	55	65	18.13	75	25	60	4.50	50	9.10	70	60.23	365	2
287	16.80	80	80	15.53	70	65	22.10	85	50	60	4.50	50	9.10	70	68.03	510	2
288	12.08	60	55	13.23	50	65	26.63	85	75	75	4.50	50	9.10	70	65.53	750	2
289	17.33	80	85	15.53	70	65	25.50	70	75	80	6.75	75	10.40	80	75.50	920	3
290	14.70	80	60	12.65	45	65	26.63	85	75	75	4.50	50	9.75	75	68.23	2650	2
291	11.03	60	45	17.83	80	75	12.47	60	0	50	0.00	0	7.15	55	48.47	40	1
292	13.13	55	70	18.40	80	80	12.47	60	0	50	0.00	0	5.85	45	49.84	45	1
293	14.18	70	65	13.80	55	65	21.53	75	50	65	2.25	25	8.45	65	60.21	55	2
294	14.18	55	80	14.38	60	65	20.97	50	70	65	2.25	25	9.10	70	60.87	62	2
295	16.28	75	80	13.80	55	65	21.53	55	75	60	0.00	0	8.45	65	60.06	64	2
296	15.23	75	70	13.80	55	65	21.53	55	75	60	2.25	25	8.45	65	61.26	70	2

297	13.65	60	70	18.40	80	80	14.17	60	0	65	0.00	0	5.85	45	52.07	80	1
298	12.08	65	50	14.38	60	65	14.73	65	0	65	2.25	25	8.45	65	51.88	201	1
299	15.23	75	70	14.95	65	65	24.93	85	75	60	2.25	25	9.10	70	66.46	240	2
300	14.18	70	65	12.65	45	65	13.60	65	0	55	2.25	25	8.45	65	51.13	300	1
301	13.13	65	60	14.95	65	65	23.80	75	75	60	4.50	50	9.10	70	65.48	367	2
302	12.08	55	60	14.38	60	65	25.50	85	75	65	4.50	50	9.10	70	65.55	382	2
303	14.18	85	50	14.38	60	65	22.67	60	75	65	4.50	50	9.10	70	64.82	478	2
304	13.65	75	55	13.80	55	65	25.50	85	75	65	4.50	50	9.10	70	66.55	536	2
305	13.65	75	55	15.53	70	65	25.50	85	75	65	4.50	50	9.10	70	68.28	750	2
306	16.28	75	80	17.25	70	80	26.63	85	75	75	6.75	75	10.40	80	77.31	1700	3
307	17.33	80	85	16.10	65	75	26.63	80	75	80	6.75	75	10.40	80	77.21	1900	3
308	16.80	75	85	15.53	60	75	27.77	90	75	80	6.75	75	10.40	80	77.24	2200	3
309	15.75	75	75	13.23	50	65	23.23	55	75	75	6.75	75	9.10	70	68.06	2500	2
310	13.13	55	70	16.68	80	65	11.90	60	0	45	0.00	0	8.45	65	50.15	52	1
311	15.75	75	75	14.95	65	65	18.70	60	50	55	2.25	25	8.45	65	60.10	55	2
312	11.55	40	70	16.10	75	65	11.90	60	0	45	0.00	0	9.10	70	48.65	65	1
313	13.65	65	65	15.53	70	65	19.83	65	50	60	2.25	25	9.10	70	60.36	70	2
314	12.08	55	60	16.68	80	65	13.60	60	0	60	0.00	0	7.15	55	49.50	70	1
315	13.65	65	65	16.68	80	65	14.17	60	0	65	0.00	0	6.50	50	50.99	135	1
316	14.70	70	70	14.38	60	65	19.83	85	25	65	2.25	25	9.10	70	60.26	240	2
317	13.13	80	45	16.10	75	65	24.37	85	75	55	4.50	50	9.10	70	67.19	320	2
318	17.33	80	85	15.53	70	65	21.53	85	50	55	4.50	50	9.10	70	67.98	400	2
319	13.65	65	65	15.53	70	65	20.40	65	50	65	4.50	50	9.10	70	63.18	450	2
320	16.28	80	75	14.38	60	65	25.50	85	75	65	4.50	50	9.10	70	69.75	480	2
321	14.18	50	85	13.80	55	65	26.07	85	75	70	4.50	50	9.10	70	67.64	650	2

322	16.80	80	80	17.83	80	75	27.20	85	75	80	6.75	75	9.75	75	78.33	1100	3
323	14.18	75	60	13.80	50	70	26.63	85	75	75	4.50	50	9.75	75	68.86	1150	2
324	16.28	75	80	16.68	70	75	26.07	75	75	80	6.75	75	9.75	75	75.52	1200	3
325	14.70	75	65	12.65	45	65	27.20	85	75	80	4.50	50	9.75	75	68.80	1300	2
326	15.23	80	65	16.68	65	80	27.77	90	75	80	6.75	75	9.75	75	76.17	1800	3
327	12.60	55	65	16.68	80	65	13.60	60	0	60	0.00	0	8.45	65	51.33	35	1
328	13.13	55	70	15.53	70	65	11.90	60	0	45	0.00	0	7.80	60	48.35	37	1
329	11.55	50	60	16.68	80	65	14.73	60	0	70	0.00	0	8.45	65	51.41	40	1
330	11.03	50	55	16.68	80	65	11.90	60	0	45	0.00	0	8.45	65	48.05	50	1
331	15.75	70	80	14.38	60	65	21.53	70	50	70	0.00	0	9.10	70	60.76	55	2
332	13.13	65	60	15.53	70	65	13.60	65	0	55	0.00	0	7.15	55	49.40	120	1
333	12.60	60	60	14.95	65	65	15.87	65	0	75	0.00	0	7.15	55	50.57	150	1
334	11.55	65	45	15.53	70	65	13.60	65	0	55	2.25	25	6.50	50	49.43	180	1
335	13.65	65	65	15.53	70	65	14.17	65	0	60	2.25	25	7.80	60	53.39	220	1
336	15.75	70	80	15.53	70	65	17.00	75	25	50	2.25	25	9.75	75	60.28	260	2
337	15.75	70	80	14.38	60	65	20.97	85	50	50	4.50	50	9.75	75	65.34	350	2
338	16.28	80	75	14.95	60	70	22.67	85	50	65	4.50	50	9.75	75	68.14	400	2
339	16.28	80	75	14.95	65	65	23.23	85	50	70	4.50	50	9.75	75	68.71	500	2
340	15.75	65	85	14.38	60	65	21.53	70	50	70	4.50	50	9.75	75	65.91	550	2
341	14.70	80	60	14.38	60	65	21.53	70	50	70	4.50	50	9.75	75	64.86	700	2
342	13.65	65	65	13.80	55	65	22.10	70	50	75	4.50	50	9.75	75	63.80	730	2
343	14.18	65	70	16.68	70	75	27.77	85	75	85	6.75	75	9.75	75	75.12	1250	3
344	13.13	60	65	15.53	75	60	14.17	65	0	60	0.00	0	8.45	65	51.27	35	1
345	14.18	70	65	15.53	75	60	13.03	65	0	50	0.00	0	7.80	60	50.53	50	1
346	12.60	55	65	14.95	65	65	23.80	100	50	60	0.00	0	9.10	70	60.45	52	2

347	14.18	50	85	15.53	70	65	22.10	85	50	60	2.25	25	9.10	70	63.15	150	2
348	14.18	70	65	12.65	45	65	22.10	85	50	60	2.25	25	9.10	70	60.28	180	2
349	14.18	75	60	13.80	55	65	22.67	65	75	60	2.25	25	9.10	70	61.99	210	2
350	12.60	65	55	14.38	60	65	22.67	65	75	60	2.25	25	9.10	70	60.99	240	2
351	16.28	80	75	13.80	55	65	19.27	85	25	60	2.25	25	9.10	70	60.69	280	2
352	15.75	80	70	14.38	60	65	19.27	85	25	60	2.25	25	9.10	70	60.74	350	2
353	15.75	85	65	14.38	60	65	19.27	85	25	60	2.25	25	9.10	70	60.74	380	2
354	13.13	80	45	13.80	55	65	20.40	60	50	70	4.50	50	9.10	70	60.93	450	2
355	13.65	80	50	13.80	55	65	26.07	85	75	70	4.50	50	9.10	70	67.12	530	2
356	13.65	70	60	13.23	50	65	26.07	85	75	70	4.50	50	9.10	70	66.54	760	2
357	17.33	80	85	13.23	50	65	22.67	50	75	75	4.50	50	9.75	75	67.47	1180	2
358	16.28	80	75	16.68	70	75	26.07	75	75	80	6.75	75	9.75	75	75.52	1200	3
359	19.43	95	90	14.95	55	75	33.43	100	95	100	9.00	100	12.35	95	89.16	22000	4
360	12.08	60	55	14.95	70	60	13.03	60	0	55	0.00	0	7.15	55	47.21	38	1
361	13.65	75	55	14.95	70	60	14.17	65	0	60	0.00	0	5.85	45	48.62	40	1
362	11.55	55	55	15.53	75	60	14.17	65	0	60	0.00	0	7.15	55	48.39	45	1
363	13.13	60	65	14.95	70	60	14.17	60	0	65	0.00	0	7.15	55	49.39	50	1
364	14.18	60	75	15.53	75	60	14.73	70	0	60	0.00	0	7.80	60	52.23	56	1
365	13.13	60	65	16.10	80	60	15.30	65	0	70	0.00	0	9.10	70	53.63	62	1
366	12.08	45	70	15.53	75	60	13.60	65	0	55	0.00	0	8.45	65	49.65	75	1
367	16.80	80	80	15.53	70	65	18.70	75	25	65	0.00	0	9.10	70	60.13	81	2
368	12.60	60	60	15.53	75	60	15.30	75	0	60	2.25	25	8.45	65	54.13	86	1
369	16.80	80	80	14.38	60	65	22.10	85	50	60	4.50	50	9.75	75	67.53	432	2
370	15.75	65	85	13.80	55	65	22.67	85	50	65	4.50	50	9.75	75	66.47	540	2
371	15.23	65	80	13.80	55	65	25.50	85	75	65	4.50	50	9.75	75	68.78	650	2

372	13.65	65	65	12.65	45	65	25.50	85	75	65	4.50	50	9.75	75	66.05	780	2
373	17.33	80	85	13.23	50	65	26.07	85	75	70	4.50	50	9.75	75	70.87	850	2
374	16.80	75	85	16.68	55	90	27.77	90	75	80	9.00	100	11.05	85	81.29	4600	3
375	13.13	65	60	15.53	75	60	12.47	65	0	45	0.00	0	7.80	60	48.92	32	1
376	13.65	65	65	16.68	85	60	11.90	65	0	40	0.00	0	6.50	50	48.73	38	1
377	16.80	80	80	14.38	60	65	20.97	85	50	50	0.00	0	9.10	70	61.24	42	2
378	11.03	60	45	15.53	75	60	13.60	65	0	55	0.00	0	5.85	45	46.00	49	1
379	11.55	55	55	15.53	75	60	14.17	65	0	60	0.00	0	7.80	60	49.04	52	1
380	13.13	70	55	15.53	70	65	23.23	85	50	70	0.00	0	8.45	65	60.33	60	2
381	14.70	60	80	13.80	55	65	23.23	85	50	70	0.00	0	8.45	65	60.18	65	2
382	13.13	60	65	13.80	60	60	14.73	65	0	65	0.00	0	8.45	65	50.11	73	1
383	16.80	80	80	14.95	65	65	20.97	85	50	50	0.00	0	9.10	70	61.82	80	2
384	14.18	65	70	16.10	65	75	15.87	65	25	50	4.50	50	7.80	60	58.44	145	1
385	14.18	80	55	14.95	65	65	21.53	85	50	55	2.25	25	9.10	70	62.01	285	2
386	13.13	80	45	13.80	55	65	24.93	85	75	60	4.50	50	9.75	75	66.11	560	2
387	14.18	70	65	13.80	55	65	25.50	85	75	65	2.25	25	9.75	75	65.48	650	2
388	14.70	75	65	14.38	60	65	25.50	85	75	65	4.50	50	9.75	75	68.83	750	2
389	16.80	80	80	12.65	45	65	25.50	85	75	65	4.50	50	9.75	75	69.20	835	2
390	17.33	80	85	16.68	60	85	27.77	90	75	80	6.75	75	9.75	75	78.27	2700	3
391	12.08	65	50	16.68	80	65	14.17	60	0	65	0.00	0	8.45	65	51.37	40	1
392	17.33	85	80	16.10	75	65	16.43	65	25	55	2.25	25	8.45	65	60.56	45	2
393	14.70	70	70	16.68	80	65	11.90	60	0	45	0.00	0	8.45	65	51.73	47	1
394	10.50	45	55	16.68	80	65	13.03	60	0	55	0.00	0	8.45	65	48.66	52	1
395	16.28	75	80	14.95	65	65	18.13	75	25	60	2.25	25	8.45	65	60.06	62	2
396	16.80	80	80	15.53	70	65	16.43	55	25	65	2.25	25	9.10	70	60.11	67	2

397	17.33	85	80	15.53	70	65	17.00	65	25	60	2.25	25	9.10	70	61.20	75	2
398	16.80	80	80	16.10	75	65	15.87	60	25	55	2.25	25	9.10	70	60.12	79	2
399	13.13	60	65	17.25	75	75	16.43	65	25	55	2.25	25	7.80	60	56.86	160	1
400	13.65	65	65	13.80	55	65	22.10	85	50	60	2.25	25	9.10	70	60.90	220	2
401	17.33	80	85	14.38	60	65	22.67	85	50	65	4.50	50	9.75	75	68.62	400	2
402	16.28	70	85	15.53	65	70	23.23	85	50	70	4.50	50	9.75	75	69.28	550	2
403	13.65	50	80	13.80	55	65	26.07	85	75	70	4.50	50	9.75	75	67.77	630	2
404	15.23	80	65	14.95	60	70	23.23	85	50	70	4.50	50	9.75	75	67.66	710	2
405	11.55	65	45	13.80	55	65	26.07	85	75	70	4.50	50	9.75	75	65.67	750	2
406	13.13	80	45	13.23	50	65	24.37	65	75	75	4.50	50	9.75	75	64.97	880	2
407	11.55	60	50	14.38	60	65	30.03	85	100	80	6.75	75	11.05	85	73.76	3750	2
408	13.13	60	65	16.10	75	65	15.30	65	0	70	0.00	0	7.15	55	51.68	33	1
409	12.60	65	55	15.53	75	60	14.17	65	0	60	0.00	0	9.10	70	51.39	36	1
410	12.08	60	55	14.38	65	60	15.30	65	0	70	0.00	0	8.45	65	50.20	40	1
411	10.50	55	45	15.53	75	60	15.30	65	0	70	0.00	0	7.80	60	49.13	42	1
412	9.98	55	40	14.95	70	60	14.17	65	0	60	0.00	0	7.80	60	46.89	45	1
413	13.13	60	65	15.53	75	60	12.47	65	0	45	0.00	0	8.45	65	49.57	50	1
414	12.08	45	70	15.53	75	60	14.17	65	0	60	0.00	0	7.15	55	48.92	55	1
415	13.65	60	70	15.53	70	65	22.67	75	50	75	0.00	0	9.10	70	60.94	60	2
416	15.23	70	75	16.10	75	65	17.57	65	25	65	2.25	25	9.10	70	60.24	73	2
417	15.75	80	70	14.95	65	65	20.40	65	50	65	0.00	0	9.10	70	60.20	105	2
418	14.70	80	60	16.10	75	65	20.40	60	50	70	0.00	0	9.10	70	60.30	115	2
419	16.28	70	85	14.95	65	65	19.83	60	50	65	0.00	0	9.10	70	60.16	120	2
420	12.60	60	60	17.83	80	75	16.43	65	25	55	2.25	25	5.85	45	54.96	122	1
421	13.13	55	70	17.25	75	75	15.87	65	25	50	0.00	0	7.80	60	54.04	135	1

422	14.18	70	65	17.25	75	75	17.00	65	25	60	2.25	25	8.45	65	59.13	160	1
423	16.80	80	80	13.80	55	65	19.83	70	50	55	2.25	25	9.10	70	61.78	265	2
424	12.60	55	65	17.83	80	75	10.77	50	0	45	0.00	0	8.45	65	49.64	35	1
425	12.60	55	65	17.83	80	75	11.90	50	0	55	0.00	0	5.85	45	48.18	36	1
426	15.23	80	65	13.80	55	65	22.10	75	75	45	0.00	0	9.10	70	60.23	40	2
427	13.65	60	70	16.68	75	70	13.03	55	0	60	0.00	0	5.85	45	49.21	45	1
428	13.13	55	70	17.83	80	75	14.17	65	0	60	0.00	0	7.15	55	52.27	48	1
429	16.28	75	80	13.80	55	65	21.53	60	75	55	0.00	0	8.45	65	60.06	55	2
430	14.18	70	65	15.53	70	65	21.53	55	75	60	0.00	0	9.10	70	60.33	60	2
431	15.75	70	80	15.53	70	65	20.97	60	75	50	0.00	0	7.80	60	60.04	70	2
432	12.08	60	55	17.25	75	75	14.17	60	0	65	0.00	0	8.45	65	51.94	75	1
433	16.80	80	80	14.95	65	65	19.27	50	50	70	0.00	0	9.10	70	60.12	80	2
434	14.70	70	70	17.83	80	75	10.77	60	0	35	0.00	0	7.80	60	51.09	88	1
435	14.18	85	50	14.38	60	65	23.80	60	75	75	0.00	0	8.45	65	60.80	93	2
436	14.18	85	50	13.80	55	65	23.80	60	100	50	0.00	0	9.10	70	60.88	150	2
437	15.23	65	80	14.38	60	65	24.37	80	75	60	2.25	25	9.10	70	65.32	370	2
438	16.80	80	80	13.80	55	65	26.63	85	75	75	4.50	50	9.75	75	71.48	450	2
439	15.75	75	75	16.10	70	70	11.90	60	0	45	0.00	0	8.45	65	52.20	40	1
440	13.65	60	70	16.10	65	75	12.47	65	0	45	0.00	0	7.15	55	49.37	45	1
441	9.45	45	45	15.53	65	70	11.90	50	0	55	0.00	0	7.15	55	44.03	50	1
442	14.18	65	70	17.25	80	70	13.03	55	0	60	0.00	0	7.80	60	52.26	56	1
443	11.03	60	45	17.83	80	75	15.30	70	0	65	0.00	0	5.85	45	50.00	63	1
444	14.70	65	75	14.95	65	65	22.67	85	50	65	0.00	0	7.80	60	60.12	72	2
445	13.65	60	70	16.68	70	75	12.47	50	0	60	0.00	0	7.15	55	49.94	88	1
446	15.23	60	85	15.53	70	65	21.53	85	50	55	0.00	0	8.45	65	60.73	91	2

447	14.18	55	80	14.95	65	65	21.53	85	50	55	2.25	25	7.80	60	60.71	200	2
448	15.23	70	75	14.95	65	65	20.40	70	50	60	2.25	25	8.45	65	61.28	230	2
449	15.23	80	65	13.80	55	65	21.53	70	50	70	4.50	50	9.75	75	64.81	560	2
450	17.33	75	90	14.95	70	60	26.63	80	75	80	9.00	100	9.75	75	77.66	920	3
451	17.33	85	80	16.68	75	70	30.03	90	80	95	9.00	100	12.35	95	85.38	3500	4
452	9.98	65	30	14.95	70	60	8.50	45	0	30	0.00	0	7.80	60	41.23	40	1
453	13.65	65	65	17.83	80	75	11.33	55	0	45	0.00	0	9.10	70	51.91	45	1
454	16.80	80	80	13.80	55	65	20.97	85	50	50	0.00	0	9.10	70	60.67	50	2
455	12.60	75	45	14.95	65	65	24.37	85	75	55	0.00	0	9.10	70	61.02	110	2
456	16.80	80	80	14.95	65	65	22.67	65	75	60	0.00	0	9.10	70	63.52	120	2
457	13.13	65	60	13.80	55	65	14.73	65	0	65	0.00	0	7.15	55	48.81	130	1
458	10.50	65	35	17.25	70	80	15.30	70	0	65	2.25	25	7.80	60	53.10	200	1
459	13.13	70	55	13.80	55	65	15.30	65	0	70	2.25	25	8.45	65	52.93	230	1
460	12.08	70	45	14.38	55	70	22.67	85	50	65	2.25	25	9.10	70	60.47	301	2
461	15.75	70	80	13.23	50	65	26.07	85	75	70	4.50	50	9.75	75	69.29	1050	2
462	16.80	80	80	13.23	50	65	22.67	50	75	75	4.50	50	9.75	75	66.94	1120	2
463	11.55	55	55	13.23	50	65	26.63	85	75	75	4.50	50	9.75	75	65.66	1180	2
464	17.33	80	85	14.95	70	60	26.63	80	75	80	9.00	100	9.75	75	77.66	1250	3
465	15.75	75	75	17.25	75	75	26.63	80	75	80	9.00	100	10.40	80	79.03	1270	3
466	16.28	80	75	16.68	70	75	27.77	90	75	80	9.00	100	10.40	80	80.12	1360	3
467	19.95	95	95	16.68	70	75	33.43	100	95	100	9.00	100	12.35	95	91.41	19000	4
468	13.65	60	70	15.53	75	60	14.73	65	0	65	0.00	0	8.45	65	52.36	32	1
469	12.60	75	45	15.53	75	60	14.73	65	0	65	0.00	0	7.80	60	50.66	45	1
470	13.65	65	65	15.53	75	60	13.60	60	0	60	0.00	0	7.15	55	49.93	65	1
471	15.23	70	75	17.25	65	85	18.70	70	50	45	0.00	0	9.10	70	60.28	75	2

472	11.55	55	55	14.95	70	60	13.60	65	0	55	0.00	0	5.85	45	45.95	130	1
473	11.55	45	65	15.53	75	60	13.60	65	0	55	0.00	0	7.15	55	47.83	180	1
474	15.75	80	70	15.53	70	65	21.53	65	75	50	2.25	25	9.10	70	64.16	240	2
475	16.80	80	80	15.53	70	65	21.53	65	75	50	2.25	25	9.10	70	65.21	260	2
476	16.80	80	80	13.80	55	65	21.53	85	50	55	2.25	25	9.10	70	63.48	350	2
477	16.28	80	75	15.53	70	65	22.10	85	50	60	2.25	25	9.10	70	65.25	360	2
478	14.70	65	75	13.80	55	65	22.67	85	50	65	2.25	25	9.10	70	62.52	470	2
479	13.65	55	75	16.10	75	65	25.50	85	75	65	2.25	25	9.10	70	66.60	520	2
480	13.65	55	75	14.38	55	70	26.07	85	75	70	4.50	50	9.75	75	68.34	685	2
481	17.85	85	85	16.68	70	75	27.20	85	75	80	9.00	100	10.40	80	81.13	1150	3
482	17.85	85	85	17.25	75	75	27.20	85	75	80	9.00	100	9.10	70	80.40	1300	3
483	17.33	85	80	17.83	75	80	26.63	80	75	80	9.00	100	9.10	70	79.88	1850	3
484	12.60	60	60	15.53	75	60	14.73	60	0	70	0.00	0	7.15	55	50.01	41	1
485	12.60	65	55	15.53	75	60	16.43	70	0	75	0.00	0	7.80	60	52.36	45	1
486	14.70	65	75	16.10	60	80	20.40	50	75	55	0.00	0	9.10	70	60.30	62	2
487	14.18	70	65	17.25	75	75	20.40	60	50	70	0.00	0	9.10	70	60.93	75	2
488	16.80	80	80	14.95	65	65	20.97	55	75	55	0.00	0	9.10	70	61.82	120	2
489	12.08	60	55	16.10	80	60	14.17	65	0	60	0.00	0	9.10	70	51.44	125	1
490	12.08	55	60	15.53	75	60	14.73	65	0	65	0.00	0	8.45	65	50.78	136	1
491	14.70	80	60	15.53	55	80	20.97	55	75	55	0.00	0	9.10	70	60.29	210	2
492	16.80	80	80	13.80	55	65	22.67	65	75	60	2.25	25	9.10	70	64.62	280	2
493	15.23	80	65	14.95	65	65	22.10	85	50	60	2.25	25	9.10	70	63.63	350	2
494	15.75	70	80	13.80	55	65	22.10	85	50	60	2.25	25	9.10	70	63.00	370	2
495	15.23	65	80	14.38	60	65	22.10	85	50	60	2.25	25	9.10	70	63.05	440	2
496	15.23	80	65	13.80	55	65	20.97	75	50	60	2.25	25	9.10	70	61.34	530	2

497	15.75	80	70	16.10	75	65	22.10	85	50	60	2.25	25	9.75	75	65.95	680	2
498	12.08	50	65	15.53	70	65	24.93	85	75	60	4.50	50	9.75	75	66.78	740	2
499	15.75	85	65	12.65	45	65	26.63	85	75	75	4.50	50	10.40	80	69.93	1250	2
500	16.80	80	80	16.68	70	75	26.63	80	75	80	9.00	100	9.10	70	78.21	1440	3
501	16.80	80	80	16.10	60	80	26.63	80	75	80	9.00	100	9.10	70	77.63	1800	3
502	12.60	55	65	15.53	75	60	15.87	75	0	65	2.25	25	8.45	65	54.69	35	1
503	13.65	70	60	15.53	75	60	13.03	65	0	50	0.00	0	7.15	55	49.36	65	1
504	15.23	80	65	13.80	55	65	20.97	65	75	45	2.25	25	8.45	65	60.69	122	2
505	12.08	70	45	16.68	85	60	14.73	65	0	65	0.00	0	6.50	50	49.98	126	1
506	16.80	80	80	14.95	65	65	23.23	85	75	45	2.25	25	9.10	70	66.33	130	2
507	16.28	75	80	14.38	60	65	20.97	65	75	45	2.25	25	8.45	65	62.32	130	2
508	13.13	70	55	15.53	75	60	14.17	65	0	60	0.00	0	5.85	45	48.67	210	1
509	12.60	65	55	14.95	65	65	16.43	75	25	45	2.25	25	8.45	65	54.68	270	1
510	14.18	80	55	13.80	55	65	21.53	85	50	55	2.25	25	9.10	70	60.86	360	2
511	14.70	70	70	13.80	55	65	21.53	75	50	65	2.25	25	9.10	70	61.38	425	2
512	15.23	70	75	14.38	60	65	22.67	85	50	65	4.50	50	9.10	70	65.87	500	2
513	16.28	80	75	12.08	40	65	22.67	85	50	65	4.50	50	9.10	70	64.62	750	2
514	16.80	80	80	13.23	50	65	21.53	70	50	70	4.50	50	9.75	75	65.81	1023	2
515	16.28	80	75	12.65	45	65	22.10	75	50	70	4.50	50	9.75	75	65.28	1069	2
516	16.80	80	80	17.83	80	75	27.77	90	75	80	9.00	100	9.10	70	80.49	1102	3
517	16.80	75	85	16.68	70	75	27.77	90	75	80	9.00	100	9.10	70	79.34	1120	3
518	17.85	85	85	15.53	70	65	26.63	80	75	80	9.00	100	9.10	70	78.11	1250	3
519	13.13	60	65	17.83	80	75	11.33	65	0	35	2.25	25	7.80	60	52.33	40	1
520	13.13	60	65	17.83	80	75	12.47	50	0	60	0.00	0	8.45	65	51.87	42	1
521	14.18	65	70	17.25	75	75	13.60	60	0	60	0.00	0	7.80	60	52.83	47	1

522	12.60	65	55	17.25	75	75	18.13	65	25	70	0.00	0	7.80	60	55.78	50	1
523	13.13	65	60	17.25	75	75	18.13	70	25	65	2.25	25	8.45	65	59.21	51	1
524	12.08	55	60	17.83	75	80	13.60	65	0	55	0.00	0	7.80	60	51.30	55	1
525	13.13	60	65	17.83	75	80	13.60	65	0	55	0.00	0	7.80	60	52.35	60	1
526	13.65	60	70	17.83	75	80	13.03	65	0	50	0.00	0	7.80	60	52.31	71	1
527	13.13	55	70	17.25	75	75	12.47	65	0	45	0.00	0	7.80	60	50.64	110	1
528	16.80	80	80	13.80	55	65	21.53	85	50	55	0.00	0	9.10	70	61.23	116	2
529	11.03	60	45	17.25	75	75	14.17	60	0	65	0.00	0	7.80	60	50.24	118	1
530	15.23	65	80	13.80	55	65	22.10	85	50	60	2.25	25	9.10	70	62.48	350	2
531	12.08	60	55	14.95	60	70	22.10	85	50	60	2.25	25	9.10	70	60.48	435	2
532	14.70	75	65	14.38	60	65	22.10	85	50	60	2.25	25	9.10	70	62.53	468	2
533	14.18	75	60	13.80	55	65	22.67	85	50	65	4.50	50	9.10	70	64.24	594	2
534	13.65	65	65	13.23	50	65	22.10	85	50	60	4.50	50	9.10	70	62.58	750	2
535	15.75	85	65	14.95	70	60	26.63	80	75	80	9.00	100	9.10	70	75.43	1250	3
536	10.50	55	45	17.25	75	75	14.17	65	0	60	0.00	0	7.80	60	49.72	32	1
537	11.03	60	45	17.25	75	75	13.03	65	0	50	0.00	0	7.80	60	49.11	38	1
538	13.13	70	55	17.25	75	75	14.73	65	0	65	2.25	25	7.80	60	55.16	45	1
539	12.60	45	75	17.25	75	75	14.17	65	0	60	0.00	0	7.80	60	51.82	52	1
540	14.70	80	60	16.10	65	75	22.10	70	75	50	0.00	0	8.45	65	61.35	53	2
541	13.13	65	60	17.25	75	75	18.13	65	25	70	0.00	0	8.45	65	56.96	72	1
542	13.65	60	70	16.68	80	65	13.03	60	0	55	2.25	25	8.45	65	54.06	130	1
543	15.23	80	65	14.95	55	75	20.97	85	50	50	0.00	0	9.10	70	60.24	170	2
544	12.08	70	45	15.53	60	75	21.53	85	50	55	2.25	25	9.10	70	60.48	240	2
545	14.18	55	80	14.95	65	65	24.37	85	75	55	2.25	25	9.10	70	64.84	310	2
546	15.75	80	70	13.80	55	65	24.93	85	75	60	2.25	25	9.10	70	65.83	360	2

547	11.55	65	45	13.80	55	65	25.50	85	75	65	2.25	25	9.10	70	62.20	390	2
548	16.80	80	80	13.80	55	65	23.80	70	75	65	2.25	25	9.10	70	65.75	500	2
549	13.65	65	65	15.53	70	65	26.07	85	75	70	4.50	50	9.75	75	69.49	710	2
550	16.80	80	80	13.23	50	65	26.07	85	75	70	4.50	50	9.10	70	69.69	760	2
551	15.75	80	70	14.95	70	60	27.20	85	75	80	9.00	100	9.10	70	76.00	1450	3
552	13.13	60	65	16.10	75	65	14.17	65	0	60	0.00	0	7.80	60	51.19	37	1
553	12.60	55	65	16.68	75	70	14.17	60	0	65	0.00	0	7.80	60	51.24	45	1
554	14.70	60	80	13.80	55	65	23.23	70	75	60	0.00	0	8.45	65	60.18	51	2
555	12.08	55	60	17.25	75	75	13.60	65	0	55	0.00	0	7.15	55	50.08	56	1
556	12.60	60	60	17.25	75	75	14.17	65	0	60	0.00	0	7.80	60	51.82	64	1
557	12.60	60	60	17.25	75	75	13.03	70	0	45	0.00	0	8.45	65	51.33	75	1
558	13.65	65	65	17.25	75	75	15.30	65	0	70	2.25	25	7.80	60	56.25	155	1
559	16.28	75	80	14.95	65	65	24.93	85	75	60	2.25	25	9.10	70	67.51	265	2
560	16.28	85	70	13.80	55	65	25.50	85	75	65	2.25	25	9.10	70	66.93	382	2
561	15.75	80	70	15.53	70	65	25.50	85	75	65	2.25	25	9.10	70	68.13	425	2
562	14.18	65	70	13.80	55	65	25.50	85	75	65	4.50	50	9.10	70	67.08	450	2
563	16.80	80	80	15.53	70	65	24.37	75	75	65	4.50	50	9.10	70	70.29	530	2
564	14.18	55	80	13.80	55	65	25.50	85	75	65	4.50	50	9.75	75	67.73	580	2
565	13.13	60	65	14.38	60	65	24.93	85	70	65	4.50	50	9.10	70	66.03	710	2
566	15.23	85	60	13.80	55	65	26.07	85	75	70	6.75	75	10.40	80	72.24	750	2
567	16.80	80	80	15.53	70	65	26.63	80	75	80	9.00	100	9.10	70	77.06	1089	3
568	14.70	70	70	14.95	65	65	18.13	75	25	60	2.25	25	8.45	65	58.48	46	1
569	12.60	60	60	17.83	75	80	12.47	65	0	45	0.00	0	8.45	65	51.34	55	1
570	12.60	55	65	17.25	75	75	14.73	65	0	65	0.00	0	8.45	65	53.03	58	1
571	16.80	80	80	13.23	50	65	21.53	60	75	55	0.00	0	9.10	70	60.66	60	2

572	16.80	80	80	13.80	55	65	21.53	60	75	55	0.00	0	9.10	70	61.23	115	2
573	12.60	60	60	18.40	80	80	14.17	60	0	65	2.25	25	7.80	60	55.22	160	1
574	12.60	55	65	15.53	75	60	14.17	65	0	60	0.00	0	7.80	60	50.09	175	1
575	14.70	70	70	16.10	65	75	20.40	65	55	60	0.00	0	9.10	70	60.30	188	2
576	14.18	55	80	14.95	55	75	19.83	65	50	60	2.25	25	9.10	70	60.31	192	2
577	12.08	50	65	14.95	50	80	22.10	65	65	65	2.25	25	9.10	70	60.48	215	2
578	12.08	55	60	16.68	70	75	20.40	70	50	60	2.25	25	9.10	70	60.50	236	2
579	16.80	80	80	13.80	55	65	19.83	65	50	60	2.25	25	9.10	70	61.78	275	2
580	16.80	80	80	13.80	55	65	19.83	65	50	60	2.25	25	9.10	70	61.78	280	2
581	15.75	70	80	16.10	75	65	20.97	70	50	65	2.25	25	9.10	70	64.17	293	2
582	11.55	55	55	12.65	45	65	23.80	85	50	75	4.50	50	9.75	75	62.25	1240	2
583	17.85	80	90	19.55	85	85	26.63	80	75	80	9.00	100	9.75	75	82.78	1350	3
584	17.85	85	85	16.68	70	75	26.63	80	75	80	9.00	100	9.75	75	79.91	1400	3
585	17.85	85	85	16.68	70	75	26.63	80	75	80	4.50	50	9.75	75	75.41	1510	3
586	11.03	50	55	14.38	65	60	14.17	65	0	60	0.00	0	7.15	55	46.72	35	1
587	14.18	70	65	14.38	60	65	13.60	65	0	55	2.25	25	6.50	50	50.90	38	1
588	13.13	65	60	13.23	50	65	14.17	55	0	70	0.00	0	8.45	65	48.97	43	1
589	16.80	80	80	15.53	70	65	23.23	75	75	55	0.00	0	9.10	70	64.66	113	2
590	13.65	65	65	14.38	60	65	11.90	60	0	45	0.00	0	8.45	65	48.38	168	1
591	13.13	65	60	13.23	60	55	15.30	65	0	70	0.00	0	9.10	70	50.75	175	1
592	11.55	55	55	14.38	60	65	13.60	70	0	50	0.00	0	7.80	60	47.33	220	1
593	14.18	65	70	13.80	55	65	23.80	75	75	60	0.00	0	9.75	75	61.53	240	2
594	14.70	75	65	14.95	65	65	19.27	75	25	70	0.00	0	9.75	75	58.67	265	1
595	17.33	80	85	15.53	70	65	23.80	75	75	60	0.00	0	9.75	75	66.40	271	2
596	15.75	85	65	14.38	60	65	24.93	85	75	60	2.25	25	9.75	75	67.06	350	2

597	16.80	80	80	13.23	50	65	26.07	85	75	70	2.25	25	9.75	75	68.09	1023	2
598	14.70	75	65	13.23	50	65	23.80	65	75	70	2.25	25	9.75	75	63.73	1065	2
599	16.28	75	80	12.65	45	65	26.63	85	75	75	2.25	25	9.75	75	67.56	1132	2
600	16.80	75	85	17.25	75	75	27.77	85	80	80	4.50	50	9.75	75	76.07	1150	3
601	19.95	95	95	18.98	75	90	33.43	100	95	100	9.00	100	12.35	95	93.71	12000	4
602	14.18	65	70	16.10	60	80	15.30	65	0	70	0.00	0	7.80	60	53.38	38	1
603	10.50	30	70	16.10	65	75	15.30	65	0	70	0.00	0	8.45	65	50.35	45	1
604	10.50	35	65	15.53	55	80	14.17	60	0	65	2.25	25	7.80	60	50.24	52	1
605	14.70	65	75	14.95	65	65	22.10	60	75	60	0.00	0	9.10	70	60.85	70	2
606	16.80	80	80	14.38	60	65	22.10	60	75	60	0.00	0	9.10	70	62.38	120	2
607	17.33	80	85	13.80	55	65	22.10	60	75	60	0.00	0	9.10	70	62.33	128	2
608	11.03	45	60	13.80	60	60	14.73	65	0	65	0.00	0	7.80	60	47.36	133	1
609	16.28	70	85	14.95	65	65	21.53	55	75	60	0.00	0	9.10	70	61.86	140	2
610	13.13	70	55	16.10	65	75	14.73	65	0	65	0.00	0	7.80	60	51.76	146	1
611	13.13	65	60	16.10	65	75	14.73	65	0	65	0.00	0	7.80	60	51.76	152	1
612	15.75	70	80	14.38	60	65	22.10	60	75	60	0.00	0	9.10	70	61.33	158	2
613	12.08	55	60	12.65	45	65	12.47	65	0	45	2.25	25	9.10	70	48.54	166	1
614	13.13	70	55	15.53	55	80	22.67	65	75	60	0.00	0	9.10	70	60.42	180	2
615	13.65	80	50	14.38	60	65	23.23	70	75	60	0.00	0	9.10	70	60.36	210	2
616	16.80	80	80	13.80	55	65	26.07	85	75	70	2.25	25	10.40	80	69.32	1100	2
617	16.28	70	85	17.83	80	75	27.77	80	80	85	4.50	50	9.75	75	76.12	1120	3
618	19.95	95	95	14.95	65	65	33.43	100	95	100	9.00	100	12.35	95	89.68	16000	4
619	12.08	50	65	17.25	85	65	14.73	65	0	65	0.00	0	7.80	60	51.86	40	1
620	10.50	45	55	14.95	55	75	13.60	65	0	55	0.00	0	7.80	60	46.85	43	1
621	17.33	85	80	15.53	70	65	24.37	75	75	65	0.00	0	9.10	70	66.32	120	2

622	11.03	60	45	15.53	60	75	14.17	60	0	65	0.00	0	8.45	65	49.17	130	1
623	14.70	80	60	15.53	70	65	23.80	75	75	60	0.00	0	8.45	65	62.48	135	2
624	9.98	35	60	15.53	65	70	14.17	60	0	65	0.00	0	7.80	60	47.47	150	1
625	16.28	75	80	13.80	55	65	23.23	75	75	55	0.00	0	9.10	70	62.41	170	2
626	16.28	80	75	12.65	50	60	24.37	75	75	65	2.25	25	9.75	75	65.29	1032	2
627	14.18	65	70	13.23	50	65	24.93	75	75	70	2.25	25	9.75	75	64.33	1069	2
628	15.23	65	80	13.23	50	65	24.37	70	75	70	2.25	25	9.75	75	64.82	1075	2
629	15.75	80	70	12.65	50	60	24.93	75	75	70	2.25	25	9.75	75	65.33	1098	2
630	16.80	75	85	16.68	70	75	26.63	80	75	80	6.75	75	9.75	75	76.61	1150	3
631	15.75	75	75	17.83	80	75	27.77	85	75	85	4.50	50	9.75	75	75.59	1178	3
632	16.28	80	75	16.68	70	75	27.77	90	75	80	6.75	75	9.75	75	77.22	1185	3
633	16.80	95	65	16.68	75	70	33.43	100	95	100	9.00	100	12.35	95	88.26	23000	4
634	13.13	65	60	16.10	65	75	14.17	65	0	60	0.00	0	8.45	65	51.84	37	1
635	12.60	60	60	13.23	45	70	14.17	65	0	60	2.25	25	7.80	60	50.04	41	1
636	10.50	45	55	14.38	55	70	14.73	65	0	65	0.00	0	9.10	70	48.71	45	1
637	13.65	60	70	14.95	55	75	13.60	65	0	55	0.00	0	9.10	70	51.30	48	1
638	14.70	65	75	16.10	65	75	15.30	65	0	70	2.25	25	8.45	65	56.80	52	1
639	10.50	45	55	13.80	60	60	16.43	75	0	70	0.00	0	9.10	70	49.83	55	1
640	7.35	35	35	13.80	65	55	19.83	75	25	75	2.25	25	8.45	65	51.68	65	1
641	16.80	80	80	13.80	55	65	22.67	75	75	50	0.00	0	8.45	65	61.72	140	2
642	11.55	55	55	12.65	45	65	15.30	65	0	70	0.00	0	8.45	65	47.95	180	1
643	11.03	60	45	16.68	65	80	24.37	75	75	65	0.00	0	8.45	65	60.52	220	2
644	11.03	55	50	16.10	60	80	24.37	75	75	65	0.00	0	9.10	70	60.59	240	2
645	11.55	60	50	17.25	70	80	22.67	60	75	65	0.00	0	9.10	70	60.57	246	2
646	16.80	80	80	14.38	60	65	23.23	75	75	55	0.00	0	8.45	65	62.86	255	2

647	15.75	80	70	13.80	55	65	23.23	75	75	55	0.00	0	9.10	70	61.88	263	2
648	14.18	55	80	13.80	55	65	23.80	75	75	60	0.00	0	9.10	70	60.88	267	2
649	13.65	70	60	14.38	60	65	24.37	75	75	65	0.00	0	9.10	70	61.49	288	2
650	13.65	50	80	14.38	60	65	24.37	80	75	60	0.00	0	9.10	70	61.49	295	2
651	15.23	80	65	14.38	60	65	25.50	80	75	70	2.25	25	9.75	75	67.10	575	2
652	12.60	60	60	12.65	45	65	14.73	65	0	65	0.00	0	7.80	60	47.78	36	1
653	11.03	45	60	16.68	80	65	14.17	65	0	60	0.00	0	8.45	65	50.32	38	1
654	12.08	60	55	14.38	55	70	12.47	65	0	45	0.00	0	8.45	65	47.37	45	1
655	11.03	60	45	14.95	65	65	13.03	70	0	45	0.00	0	7.80	60	46.81	80	1
656	14.18	55	80	15.53	55	80	22.67	80	75	45	0.00	0	9.10	70	61.47	125	2
657	11.03	60	45	13.80	60	60	14.73	65	0	65	0.00	0	7.15	55	46.71	132	1
658	13.13	70	55	14.95	65	65	14.17	65	0	60	2.25	25	6.50	50	50.99	139	1
659	15.23	80	65	13.80	55	65	23.23	80	75	50	0.00	0	8.45	65	60.71	210	2
660	14.70	75	65	15.53	70	65	23.80	75	75	60	0.00	0	9.10	70	63.13	245	2
661	15.75	70	80	13.80	55	65	23.80	80	75	55	2.25	25	9.10	70	64.70	306	2
662	11.55	55	55	15.53	55	80	22.67	60	75	65	2.25	25	9.10	70	61.09	411	2
663	15.23	65	80	12.65	45	65	24.37	80	75	60	2.25	25	9.10	70	63.59	445	2
664	14.18	85	50	13.23	50	65	21.53	55	75	60	2.25	25	9.75	75	60.93	500	2
665	14.18	80	55	13.80	55	65	23.80	80	75	55	2.25	25	9.10	70	63.13	560	2
666	14.18	80	55	13.23	50	65	24.93	80	75	65	4.50	50	9.75	75	66.58	605	2
667	16.28	75	80	13.23	50	65	26.07	85	75	70	2.25	25	9.10	70	66.92	711	2
668	16.28	80	75	16.68	70	75	27.20	85	75	80	6.75	75	9.75	75	76.65	1100	3
669	18.90	95	85	16.68	75	70	32.30	100	95	90	9.00	100	12.35	95	89.23	18000	4
670	13.13	55	70	14.95	65	65	9.63	55	0	30	0.00	0	8.45	65	46.16	41	1
671	12.60	55	65	15.53	70	65	11.90	60	0	45	0.00	0	8.45	65	48.48	46	1

672	13.13	65	60	15.53	80	55	14.73	65	0	65	0.00	0	9.10	70	52.48	58	1
673	13.13	70	55	16.10	75	65	11.90	70	0	35	0.00	0	7.80	60	48.93	72	1
674	9.45	45	45	14.95	65	65	15.30	75	25	35	2.25	25	8.45	65	50.40	135	1
675	15.75	70	80	15.53	55	80	20.97	75	75	35	0.00	0	9.10	70	61.34	220	2
676	13.65	80	50	14.95	65	65	24.37	85	75	55	0.00	0	9.10	70	62.07	265	2
677	15.23	65	80	13.80	55	65	22.10	75	75	45	0.00	0	9.10	70	60.23	278	2
678	15.23	80	65	14.95	65	65	24.37	85	75	55	0.00	0	9.10	70	63.64	285	2
679	14.70	60	80	14.95	65	65	24.93	85	75	60	2.25	25	9.10	70	65.93	360	2
680	16.28	80	75	13.80	55	65	24.37	85	75	55	2.25	25	9.10	70	65.79	530	2
681	16.28	80	75	14.95	55	75	22.67	85	50	65	2.25	25	9.75	75	65.89	700	2
682	14.18	55	80	13.23	50	65	23.23	85	50	70	2.25	25	11.05	85	63.93	1120	2
683	12.60	50	70	12.65	45	65	23.80	85	50	75	2.25	25	10.40	80	61.70	1170	2
684	17.33	85	80	12.65	45	65	23.23	80	50	75	2.25	25	10.40	80	65.86	1200	2
685	17.33	85	80	17.25	75	75	26.63	80	75	80	4.50	50	10.40	80	76.11	1300	3
686	16.28	75	80	17.25	75	75	26.63	80	75	80	4.50	50	10.40	80	75.06	1350	3
687	18.38	95	80	16.68	80	65	33.43	100	95	100	9.00	100	12.35	95	89.83	23000	4
688	11.03	40	65	14.95	65	65	15.87	65	0	75	0.00	0	8.45	65	50.29	46	1
689	11.03	45	60	15.53	55	80	14.17	60	0	65	0.00	0	9.10	70	49.82	52	1
690	13.65	65	65	14.38	45	80	14.17	60	0	65	0.00	0	9.10	70	51.29	58	1
691	8.93	30	55	16.68	65	80	14.73	60	0	70	0.00	0	7.15	55	47.48	60	1
692	16.80	75	85	14.95	50	80	19.83	80	50	45	0.00	0	9.10	70	60.68	200	2
693	15.23	65	80	16.10	60	80	20.40	80	50	50	0.00	0	8.45	65	60.18	210	2
694	12.60	55	65	16.10	60	80	10.77	60	0	35	2.25	25	6.50	50	48.22	215	1
695	15.75	65	85	16.10	60	80	19.83	75	50	50	0.00	0	8.45	65	60.13	220	2
696	15.75	70	80	14.95	65	65	20.40	80	50	50	0.00	0	9.10	70	60.20	245	2

697	17.85	80	90	14.95	65	65	21.53	65	75	50	0.00	0	8.45	65	62.78	260	2
698	15.75	85	65	13.80	55	65	23.23	80	75	50	0.00	0	9.10	70	61.88	275	2
699	14.18	55	80	12.65	45	65	24.93	80	75	65	2.25	25	9.75	75	63.76	1050	2
700	11.55	65	45	13.23	50	65	25.50	80	75	70	2.25	25	9.75	75	62.28	1080	2
701	12.60	80	40	14.38	60	65	26.07	80	75	75	2.25	25	9.75	75	65.04	1095	2
702	17.33	80	85	17.25	75	75	26.07	80	70	80	4.50	50	10.40	80	75.54	1120	3
703	16.80	75	85	16.68	70	75	28.33	95	75	80	4.50	50	10.40	80	76.71	1200	3
704	17.33	95	70	16.68	80	65	33.43	100	95	100	9.00	100	12.35	95	88.78	31000	4
705	12.60	60	60	13.23	35	80	11.90	60	0	45	0.00	0	7.80	60	45.53	40	1
706	10.50	55	45	16.68	65	80	14.17	60	0	65	0.00	0	7.15	55	48.49	42	1
707	10.50	45	55	17.25	70	80	10.77	60	0	35	0.00	0	8.45	65	46.97	45	1
708	11.03	35	70	18.98	85	80	14.17	60	0	65	0.00	0	7.15	55	51.32	54	1
709	11.03	55	50	16.68	65	80	14.17	60	0	65	0.00	0	9.10	70	50.97	60	1
710	17.33	80	85	13.80	55	65	24.37	85	75	55	0.00	0	8.45	65	63.94	72	2
711	15.75	70	80	15.53	70	65	24.37	85	75	55	0.00	0	9.10	70	64.74	112	2
712	13.13	50	75	15.53	70	65	24.37	85	75	55	0.00	0	9.10	70	62.12	138	2
713	15.23	75	70	13.80	55	65	22.67	70	75	55	0.00	0	8.45	65	60.14	146	2
714	15.23	80	65	13.80	55	65	22.67	65	75	60	0.00	0	9.10	70	60.79	155	2
715	13.13	65	60	15.53	55	80	14.17	60	0	65	0.00	0	8.45	65	51.27	160	1
716	11.55	60	50	16.68	65	80	13.60	65	0	55	2.25	25	6.50	50	50.58	225	1
717	16.80	80	80	15.53	70	65	22.10	65	75	55	0.00	0	9.75	75	64.18	270	2
718	13.13	80	45	16.10	65	75	22.67	70	75	55	2.25	25	7.80	60	61.94	278	2
719	15.23	65	80	13.80	55	65	22.67	65	75	60	0.00	0	9.10	70	60.79	285	2
720	17.33	85	80	17.25	75	75	27.20	80	80	80	4.50	50	10.40	80	76.68	1550	3
721	12.60	60	60	16.68	65	80	13.03	60	0	55	0.00	0	5.85	45	48.16	32	1

722	12.60	65	55	15.53	55	80	13.60	60	0	60	0.00	0	5.85	45	47.58	38	1
723	15.23	70	75	12.65	45	65	15.30	65	0	70	0.00	0	7.15	55	50.33	45	1
724	12.60	55	65	12.65	45	65	15.30	65	0	70	0.00	0	8.45	65	49.00	47	1
725	12.60	70	50	17.25	75	75	21.53	65	75	50	0.00	0	9.10	70	60.48	55	2
726	10.50	45	55	14.95	65	65	11.33	65	0	35	0.00	0	8.45	65	45.23	61	1
727	11.55	55	55	15.53	70	65	12.47	65	0	45	0.00	0	7.15	55	46.69	74	1
728	9.98	60	35	14.38	60	65	14.17	65	0	60	0.00	0	7.15	55	45.67	80	1
729	10.50	35	65	16.10	75	65	18.13	75	25	60	2.25	25	8.45	65	55.43	225	1
730	16.28	75	80	14.38	60	65	24.37	85	75	55	0.00	0	9.10	70	64.12	265	2
731	11.55	65	45	13.80	55	65	24.93	85	75	60	2.25	25	9.10	70	61.63	460	2
732	15.23	65	80	13.80	55	65	24.93	85	75	60	2.25	25	9.75	75	65.96	470	2
733	13.13	80	45	13.23	50	65	24.93	85	75	60	2.25	25	9.75	75	63.28	550	2
734	16.28	75	80	13.80	55	65	23.23	85	50	70	2.25	25	9.75	75	65.31	680	2
735	15.23	80	65	13.23	50	65	22.67	85	50	65	4.50	50	9.10	70	64.72	785	2
736	15.75	85	65	13.23	50	65	22.10	75	50	70	4.50	50	9.75	75	65.33	820	2
737	16.80	80	80	13.23	50	65	24.93	75	75	70	2.25	25	9.75	75	66.96	865	2
738	13.65	55	75	13.23	50	65	25.50	75	75	75	2.25	25	9.75	75	64.38	910	2
739	12.60	60	60	15.53	65	70	14.17	65	0	60	0.00	0	8.45	65	50.74	55	1
740	17.33	85	80	17.83	80	75	26.63	80	75	80	4.50	50	10.40	80	76.68	1120	3
741	15.23	65	80	14.38	55	70	21.53	75	75	40	0.00	0	9.10	70	60.23	130	2
742	14.18	70	65	14.95	65	65	11.90	70	0	35	0.00	0	7.80	60	48.83	135	1
743	17.33	80	85	13.80	55	65	22.10	75	75	45	0.00	0	9.10	70	62.33	200	2
744	15.75	80	70	15.53	70	65	23.80	85	75	50	0.00	0	9.75	75	64.83	210	2
745	14.18	65	70	14.95	65	65	23.80	85	75	50	0.00	0	9.10	70	62.03	235	2
746	15.75	80	70	14.38	60	65	23.80	85	75	50	0.00	0	9.10	70	63.03	255	2

747	16.80	80	80	13.80	55	65	24.37	85	75	55	0.00	0	9.10	70	64.07	268	2
748	15.75	70	80	13.80	55	65	24.37	80	75	60	0.00	0	9.10	70	63.02	275	2
749	12.08	60	55	15.53	60	75	23.80	80	75	55	0.00	0	9.10	70	60.50	280	2
750	15.23	65	80	13.80	55	65	23.23	80	75	50	0.00	0	9.10	70	61.36	290	2
751	13.65	50	80	12.08	40	65	26.07	85	75	70	2.25	25	9.75	75	63.79	1020	2
752	13.65	65	65	13.23	50	65	26.63	85	75	75	2.25	25	10.40	80	66.16	1035	2
753	15.75	70	80	16.68	70	75	26.63	80	75	80	6.75	75	10.40	80	76.21	1055	3
754	17.33	80	85	16.68	70	75	27.77	90	75	80	4.50	50	10.40	80	76.67	1088	3
755	19.95	95	95	19.55	85	85	33.43	100	95	100	9.00	100	12.35	95	94.28	23000	4
756	14.18	70	65	13.80	60	60	11.33	65	0	35	0.00	0	7.15	55	46.46	40	1
757	12.08	60	55	15.53	70	65	10.77	65	0	30	2.25	25	6.50	50	47.12	45	1
758	10.50	55	45	12.65	45	65	11.33	55	0	45	0.00	0	8.45	65	42.93	48	1
759	9.98	55	40	14.38	55	70	15.30	65	0	70	0.00	0	8.45	65	48.10	101	1
760	13.13	60	65	14.38	60	65	15.30	70	0	65	0.00	0	7.80	60	50.60	136	1
761	12.60	55	65	14.95	70	60	14.17	65	0	60	0.00	0	7.15	55	48.87	145	1
762	16.80	80	80	13.80	55	65	22.10	85	75	35	0.00	0	9.10	70	61.80	150	2
763	9.98	65	30	13.80	55	65	12.47	65	0	45	2.25	25	6.50	50	44.99	155	1
764	14.70	70	70	14.95	65	65	13.60	55	0	65	0.00	0	8.45	65	51.70	160	1
765	10.50	50	50	12.65	45	65	26.07	85	75	70	2.25	25	9.75	75	61.22	1103	2
766	14.70	70	70	14.38	60	65	26.63	85	75	75	2.25	25	9.75	75	67.71	1105	2
767	12.60	55	65	12.65	50	60	24.37	75	70	70	6.75	75	10.40	80	66.77	1009	2
768	13.65	65	65	18.40	85	75	26.63	80	75	80	9.00	100	10.40	80	78.08	1020	3
769	17.33	80	85	16.10	65	75	26.63	80	75	80	4.50	50	10.40	80	74.96	1055	3
770	16.28	75	80	17.25	75	75	27.20	85	75	80	6.75	75	9.10	70	76.58	1065	3
771	16.49	82	75	16.68	70	75	26.63	80	75	80	6.75	75	9.10	70	75.64	1070	3

772	17.85	80	90	18.40	85	75	27.77	90	75	80	2.25	25	9.75	75	76.02	1175	3
773	19.95	95	95	20.70	85	95	33.43	100	95	100	9.00	100	12.35	95	95.43	12000	4
