# Supplementary material for: Empirical analysis of pig welfare levels and their impact on pig breeding efficiency—Based on 773 pig farmers’ survey data
Source: PLoS One. 2017 Dec 27;12(12):e0190108. doi: 10.1371/journal.pone.0190108 (PMC5744959; doi:10.1371/journal.pone.0190108)
Supplement: S3 File — (PDF) [file pone.0190108.s003.pdf]

S3: Average input and output of the 773 pig farmers every year for each pig

Sample No.	Farm Scale(head/farm)	output value of pig （rmb/head）	young piglet cost （rmb/head）	feed costs （rmb/head）	Labour costs （rmb/head）	epidemic prevention cost （rmb/head）	death penalty （rmb/head）	power costs （rmb/head）	other costs （rmb/head）	Total input （rmb/head）
1	42	1779.11	563.84	939.19	106.30	3.85	12.27	4.88	11.86	1642.19
2	75	1768.36	564.01	926.04	119.06	3.90	12.13	4.68	12.11	1641.93
3	78	1771.31	564.35	915.15	119.16	3.94	12.09	4.69	11.84	1631.22
4	89	1781.15	564.44	913.12	119.33	3.98	12.42	4.82	12.26	1630.37
5	91	1768.71	564.80	913.00	119.60	4.03	11.96	5.06	12.29	1630.74
6	334	1782.28	565.07	913.09	119.68	4.10	13.91	5.18	12.42	1633.45
7	446	1766.55	564.84	894.57	119.89	4.44	10.67	4.98	12.50	1611.89
8	532	1770.66	564.95	915.22	119.94	4.69	10.00	4.89	12.73	1632.42
9	641	1773.81	510.99	890.97	173.50	19.08	8.81	6.38	16.78	1626.51
10	783	1782.28	510.86	892.81	173.54	19.30	11.70	6.51	17.13	1631.85
11	806	1777.59	510.45	903.64	173.83	19.46	13.97	6.83	17.21	1645.39
12	1269	1708.68	506.97	833.67	173.92	19.81	12.85	6.85	17.30	1571.37
13	1341	1696.91	506.08	807.75	174.26	20.22	12.13	6.96	17.79	1545.19
14	1555	1761.54	505.74	892.13	174.39	20.35	12.28	6.17	17.98	1629.04
15	1603	1738.35	505.53	863.44	174.55	20.63	8.86	6.69	18.15	1597.85
16	1776	1757.59	505.39	870.89	174.82	20.84	12.79	6.76	18.31	1609.80
17	1897	1741.32	504.51	865.23	175.17	21.02	9.62	6.09	18.39	1600.03

18	2367	1750.40	504.29	873.14	176.09	21.20	11.27	5.81	18.50	1610.30
19	38	1774.68	566.23	909.91	119.56	3.71	12.12	4.76	12.06	1628.35
20	67	1760.25	567.05	892.94	119.72	4.89	11.98	4.38	12.09	1613.05
21	75	1765.61	567.39	901.56	119.96	4.40	12.42	3.95	12.05	1621.73
22	82	1771.38	567.61	904.99	120.07	4.31	12.48	3.39	12.20	1625.05
23	128	1756.65	567.78	896.01	120.12	4.65	12.19	5.47	12.74	1618.96
24	245	1737.61	568.06	879.23	120.27	4.34	10.73	4.92	12.92	1600.47
25	467	1744.36	568.54	871.53	120.35	4.19	11.79	5.41	13.01	1594.82
26	523	1741.62	568.70	862.01	121.14	4.52	12.57	5.56	11.59	1586.09
27	655	1748.34	509.06	865.27	170.56	18.52	11.98	6.68	16.50	1598.57
28	860	1756.54	508.49	889.83	173.35	18.70	10.71	6.26	16.81	1624.15
29	1120	1718.58	506.47	831.78	175.13	19.03	10.33	6.53	16.90	1566.17
30	1185	1683.26	507.00	814.49	175.25	19.45	12.96	6.08	16.99	1552.22
31	1240	1708.54	506.84	836.07	175.33	20.28	12.29	6.26	17.24	1574.31
32	1420	1694.72	507.56	805.82	175.46	21.03	12.59	6.68	17.32	1546.46
33	1450	1691.61	507.74	805.56	175.59	21.40	12.20	6.97	17.35	1546.81
34	35	1771.58	563.97	912.72	102.58	2.31	12.28	3.52	8.82	1606.20
35	40	1757.66	564.46	923.37	105.89	3.16	12.36	3.99	10.11	1623.34
36	43	1764.44	564.85	928.01	106.33	3.70	10.79	3.19	10.44	1627.31
37	51	1763.81	565.09	927.97	106.55	3.79	9.68	4.28	11.20	1628.56
38	56	1760.39	565.36	909.47	106.77	3.91	13.00	4.56	11.41	1614.48
39	68	1750.28	565.58	911.66	106.90	4.19	12.11	4.79	12.08	1617.31
40	70	1755.57	565.80	913.94	107.55	4.44	10.73	4.92	12.26	1619.64
41	76	1763.45	566.14	913.06	107.83	4.52	12.91	5.07	12.79	1622.32
42	85	1748.37	566.49	905.03	108.26	4.60	12.06	5.26	13.20	1614.90

43	110	1741.98	566.65	909.74	108.49	4.71	12.17	5.38	13.34	1620.48
44	325	1728.91	566.86	892.21	108.74	4.85	12.50	5.49	13.39	1604.04
45	480	1723.46	566.99	882.81	109.53	5.31	9.62	5.71	13.49	1593.46
46	550	1721.75	567.04	880.56	110.30	5.38	12.26	5.79	13.64	1594.97
47	620	1718.61	507.74	853.41	179.57	14.89	12.35	6.37	15.11	1589.44
48	890	1705.24	507.43	829.19	181.73	16.26	12.20	6.55	15.45	1568.81
49	4200	1661.48	504.85	773.75	186.24	19.70	10.82	7.12	17.10	1519.58
50	6600	1584.74	503.56	694.46	187.76	20.02	12.87	7.29	17.69	1443.65
51	45	1737.36	560.43	876.22	123.96	6.39	12.12	4.45	8.99	1592.56
52	48	1726.87	560.60	872.75	124.48	6.49	11.67	4.01	9.92	1589.92
53	55	1741.28	561.35	884.97	126.07	6.68	11.19	5.67	10.24	1606.17
54	56	1760.41	561.49	899.16	127.56	7.22	12.33	4.38	10.39	1622.53
55	69	1729.49	561.61	861.83	131.75	7.30	12.18	3.12	9.46	1587.25
56	78	1740.95	562.00	863.21	134.19	7.81	11.06	7.26	9.54	1595.07
57	79	1721.38	562.44	845.88	134.48	8.04	9.65	6.28	9.82	1576.59
58	86	1740.28	562.76	870.44	134.63	8.40	12.21	5.59	13.14	1607.17
59	310	1743.29	553.63	867.25	140.53	8.51	12.29	5.96	12.86	1601.03
60	460	1725.88	537.38	864.56	143.92	9.09	12.79	6.42	13.61	1587.77
61	540	1740.40	528.26	866.81	146.34	10.00	12.85	6.47	12.41	1583.14
62	680	1726.37	509.09	861.07	157.77	15.95	12.28	6.66	15.83	1578.65
63	830	1705.24	501.74	834.59	170.30	16.81	12.51	6.85	16.78	1559.58
64	5500	1659.72	497.94	785.05	179.39	18.02	12.43	6.75	17.74	1517.32
65	45	1727.37	553.09	883.63	117.33	6.78	10.67	3.97	10.69	1586.16
66	55	1734.20	552.47	880.44	117.46	6.90	12.39	4.43	10.93	1585.02
67	58	1737.86	551.88	882.38	117.70	7.02	11.68	5.49	11.42	1587.57

68	64	1715.62	551.80	861.79	117.88	7.19	11.28	5.88	12.84	1568.66
69	66	1724.70	551.45	872.40	117.95	7.26	12.49	5.69	12.10	1579.34
70	72	1754.34	551.36	905.96	118.02	7.30	12.38	5.85	11.56	1612.43
71	78	1741.19	551.11	898.83	118.46	7.43	11.82	6.02	12.38	1606.05
72	80	1724.11	550.94	879.83	118.69	7.52	12.70	6.07	9.59	1585.34
73	93	1700.36	550.63	851.16	120.37	7.64	10.01	6.09	11.24	1557.14
74	206	1717.68	531.46	871.48	126.59	11.85	11.88	6.12	13.20	1572.58
75	334	1682.67	523.99	836.75	131.90	12.24	11.28	6.26	13.36	1535.78
76	455	1674.39	517.56	840.95	137.56	13.09	12.23	6.28	13.39	1541.06
77	582	1671.50	506.96	829.89	152.90	16.09	10.92	7.09	13.99	1537.84
78	627	1660.39	505.77	805.82	157.44	16.88	12.09	7.78	18.44	1524.22
79	768	1654.77	503.50	806.23	165.19	17.33	12.70	8.37	17.11	1530.43
80	850	1657.64	501.81	803.05	171.55	17.39	11.19	7.91	17.72	1530.62
81	7500	1648.38	496.55	782.43	183.96	17.82	12.16	6.17	18.19	1517.28
82	38	1772.90	550.19	926.51	120.85	7.14	12.26	2.56	8.52	1628.03
83	48	1760.67	553.94	908.71	120.37	7.52	12.00	3.53	8.81	1614.88
84	60	1741.58	546.07	890.66	121.72	7.69	12.18	3.29	8.95	1590.56
85	80	1743.39	544.88	897.42	122.14	7.82	11.83	3.67	9.84	1597.60
86	85	1737.41	544.46	893.19	122.45	7.93	11.29	3.86	9.99	1593.17
87	85	1751.24	537.97	911.53	116.60	8.05	12.78	4.09	10.89	1601.91
88	91	1740.57	534.83	908.71	117.85	8.14	11.03	4.18	10.99	1595.73
89	220	1727.47	532.74	900.27	118.42	8.74	9.65	4.38	13.14	1587.34
90	240	1730.66	531.61	898.12	118.96	9.02	13.17	4.52	13.43	1588.83
91	320	1732.35	531.09	900.56	119.27	9.26	12.28	4.65	13.70	1590.81
92	470	1728.52	529.45	897.57	120.25	9.39	12.06	4.81	14.06	1587.59

93	530	1724.44	518.07	881.14	137.34	12.90	12.01	5.96	14.22	1581.64
94	860	1727.29	504.49	895.28	146.96	14.19	12.61	6.59	14.91	1595.03
95	1350	1714.39	502.60	871.61	159.89	15.79	11.33	6.27	15.80	1583.29
96	1470	1710.19	501.78	863.97	160.08	15.95	11.79	6.23	16.42	1576.22
97	1620	1694.65	498.43	847.40	163.43	16.43	11.20	6.36	16.72	1559.97
98	1850	1690.87	496.88	847.61	164.28	16.69	12.18	6.52	17.54	1561.70
99	40	1741.53	562.93	868.85	125.89	4.95	10.96	5.03	11.03	1589.64
100	55	1744.16	561.73	873.94	126.24	5.02	10.31	5.05	11.44	1593.73
101	75	1740.59	560.65	865.63	126.86	5.33	12.39	5.38	12.11	1588.35
102	160	1735.36	559.49	864.11	129.66	5.46	11.82	5.67	12.19	1588.40
103	280	1731.74	558.90	857.08	133.78	6.40	12.58	5.76	12.45	1586.95
104	340	1727.30	537.38	875.33	136.26	6.86	11.26	6.01	12.82	1585.92
105	460	1720.96	529.60	873.78	138.63	7.74	10.01	6.08	13.36	1579.20
106	530	1725.27	526.45	873.69	142.49	8.85	12.09	6.29	13.88	1583.74
107	620	1715.38	514.09	868.51	146.34	12.10	12.42	6.37	15.01	1574.84
108	750	1722.24	510.49	876.86	147.47	12.69	11.98	6.42	15.44	1581.35
109	1050	1686.72	505.60	849.27	153.98	14.44	10.77	6.82	15.50	1556.38
110	1100	1692.19	502.48	855.30	154.25	14.49	12.01	6.99	15.55	1561.07
111	1400	1694.41	501.53	853.56	154.39	14.80	12.68	7.06	15.83	1559.85
112	1700	1703.06	500.74	864.23	154.90	15.14	12.12	7.11	16.02	1570.26
113	1750	1678.84	499.43	827.14	155.04	15.18	11.96	7.13	16.38	1532.26
114	1820	1698.55	498.75	858.85	157.48	15.52	10.87	7.27	16.55	1565.29
115	2100	1672.30	497.83	833.22	158.55	16.00	12.52	7.35	16.72	1542.19
116	32000	1660.01	492.77	823.99	163.40	16.26	12.19	7.48	16.91	1533.00
117	35	1761.38	541.57	937.43	113.16	3.91	11.99	4.32	12.24	1624.62

118	38	1750.25	541.69	930.05	114.48	3.99	12.08	4.36	12.36	1619.01
119	50	1757.67	542.43	932.85	115.03	4.25	11.47	4.43	12.41	1622.87
120	62	1755.65	541.17	923.61	115.50	4.31	12.29	4.46	12.45	1613.79
121	68	1757.50	540.64	927.59	115.86	4.38	11.92	4.58	12.46	1617.43
122	75	1751.58	540.10	924.64	116.24	4.43	11.98	4.67	11.74	1613.80
123	85	1748.37	539.44	923.14	116.92	4.48	11.86	4.81	12.55	1613.20
124	245	1744.57	538.68	920.69	117.28	4.80	11.09	5.38	13.39	1611.31
125	352	1736.67	538.36	909.32	118.15	4.88	12.27	5.87	13.62	1602.47
126	468	1735.17	526.36	911.05	119.24	5.12	11.98	6.03	14.51	1594.29
127	577	1724.92	517.69	914.33	120.09	5.35	12.16	6.19	14.92	1590.73
128	693	1718.77	494.87	874.01	173.26	15.04	11.50	6.56	16.13	1591.37
129	707	1725.25	493.94	877.53	174.50	13.80	11.82	6.69	16.66	1594.94
130	736	1714.91	491.81	861.32	175.94	15.12	12.55	6.77	16.71	1580.22
131	752	1708.82	491.57	856.29	177.08	14.75	9.80	6.82	16.75	1573.06
132	780	1720.35	491.37	865.85	178.13	16.09	11.99	6.88	16.78	1587.09
133	860	1703.68	491.24	847.81	179.07	16.76	11.89	6.99	17.12	1570.88
134	3750	1675.45	487.00	829.42	180.09	16.23	11.52	7.26	17.35	1548.87
135	32	1763.37	535.17	936.79	115.94	3.78	11.88	4.61	11.77	1619.94
136	38	1760.89	534.00	933.93	117.15	3.61	12.65	4.66	11.95	1617.95
137	46	1757.91	533.56	935.07	117.48	3.54	9.02	4.75	12.26	1615.68
138	47	1753.67	533.33	929.33	118.07	3.90	9.75	4.81	12.68	1611.87
139	60	1748.95	532.90	922.19	119.26	4.22	10.93	4.83	12.73	1607.06
140	65	1746.34	532.68	917.13	120.35	4.29	11.69	4.87	12.86	1603.87
141	83	1740.88	530.64	914.23	120.96	4.35	11.19	4.89	13.25	1599.51
142	180	1734.47	527.86	906.59	122.30	4.40	11.72	5.49	14.69	1593.05

143	240	1730.26	527.49	906.42	124.42	4.69	12.78	5.61	14.75	1596.16
144	260	1728.38	526.71	904.08	125.04	4.92	12.08	5.66	14.77	1593.26
145	370	1726.69	524.78	903.47	126.12	5.03	11.93	5.82	15.15	1592.30
146	450	1724.12	513.09	909.39	127.25	5.46	11.70	5.91	15.48	1588.28
147	780	1721.16	496.94	869.58	170.16	15.31	11.57	6.26	16.03	1585.85
148	1150	1695.25	495.55	848.72	170.47	15.46	11.99	6.53	16.56	1565.28
149	1250	1699.00	495.43	852.76	171.97	15.62	11.52	6.56	16.61	1570.47
150	1850	1703.48	492.79	853.10	173.23	15.98	13.45	6.99	16.72	1572.26
151	2100	1704.49	492.60	854.56	174.49	16.25	12.80	7.08	16.75	1574.53
152	2250	1713.35	491.73	864.43	178.14	16.39	12.05	7.22	17.08	1587.04
153	50	1760.98	539.85	926.46	122.86	3.54	11.19	4.47	10.75	1619.12
154	55	1763.27	541.39	925.38	124.25	3.92	10.82	4.52	11.15	1621.43
155	65	1772.24	541.10	934.23	125.26	4.31	9.70	4.55	11.21	1630.36
156	78	1737.29	540.46	896.00	125.95	4.39	12.08	4.73	11.48	1595.09
157	120	1749.70	540.17	905.90	127.02	4.70	11.50	5.28	12.34	1606.91
158	125	1743.45	539.49	896.36	129.95	4.85	12.18	5.39	12.42	1600.64
159	135	1737.52	539.87	889.58	132.18	5.02	11.83	5.42	12.55	1596.45
160	140	1741.16	539.27	892.82	134.45	5.24	11.29	5.51	12.64	1601.22
161	160	1740.57	536.96	895.79	134.80	5.35	12.78	5.56	12.72	1603.96
162	290	1738.41	536.50	892.11	136.45	5.40	11.03	5.79	13.45	1600.73
163	1300	1737.87	504.80	889.01	163.56	14.16	9.65	6.27	15.18	1602.63
164	1450	1734.28	501.49	875.99	171.07	15.05	13.17	6.35	15.53	1598.65
165	1480	1731.46	497.35	877.22	173.42	15.81	12.28	6.46	15.65	1598.19
166	1520	1730.81	496.87	873.66	176.00	15.96	12.06	6.62	15.72	1596.89
167	1680	1727.44	495.49	868.79	176.90	16.45	12.01	6.68	15.86	1592.18

168	2200	1724.66	492.44	864.90	178.13	16.70	12.61	7.03	16.75	1588.56
169	32	1751.90	540.16	918.80	118.08	3.69	11.33	4.43	10.29	1606.78
170	40	1758.55	539.57	923.70	118.97	3.74	11.79	4.48	10.52	1612.77
171	45	1741.42	548.29	898.61	119.46	4.19	11.20	4.52	10.93	1597.20
172	51	1747.44	549.04	902.41	120.53	4.46	12.18	4.57	11.15	1604.34
173	55	1718.85	549.18	880.67	120.69	4.48	10.96	4.61	11.34	1581.93
174	72	1738.86	549.30	896.32	121.09	4.50	10.31	4.72	11.38	1597.62
175	85	1730.29	484.24	950.27	122.16	4.56	12.39	4.83	11.66	1590.11
176	253	1727.47	530.67	901.79	123.04	4.91	11.82	5.52	12.35	1590.10
177	327	1729.84	528.87	903.65	125.43	5.13	10.52	5.66	12.47	1591.73
178	336	1731.45	529.09	903.73	126.25	5.24	11.26	5.85	12.53	1593.95
179	345	1730.66	539.84	888.97	126.93	5.29	10.01	5.89	12.65	1589.58
180	405	1727.69	540.11	887.55	128.15	5.45	12.87	5.98	12.92	1593.03
181	425	1722.17	540.66	879.29	128.94	5.48	12.12	6.06	13.48	1586.03
182	580	1718.90	539.36	841.18	132.32	5.52	44.91	6.17	14.63	1584.09
183	610	1716.38	505.61	857.97	167.43	13.82	11.19	6.69	15.54	1578.25
184	735	1722.45	503.34	862.76	170.15	14.25	12.33	6.72	15.71	1585.26
185	780	1733.34	501.65	872.32	174.09	14.74	12.18	6.76	15.94	1597.68
186	3500	1710.52	496.39	850.37	181.16	17.00	11.62	7.27	16.91	1580.72
187	38	1782.58	549.94	933.15	115.16	3.44	12.03	4.37	10.27	1628.36
188	40	1793.37	549.35	940.42	118.97	3.49	12.08	4.43	10.65	1639.39
189	45	1781.68	558.07	918.65	120.45	3.81	11.86	4.47	11.04	1628.35
190	56	1772.29	558.82	907.11	121.17	3.96	11.98	4.51	11.16	1618.71
191	62	1770.81	558.96	903.32	122.48	4.05	11.63	4.56	11.25	1616.25
192	68	1773.48	559.08	905.12	122.85	4.14	12.78	4.67	11.53	1620.17

193	74	1773.67	494.02	969.39	125.00	4.25	12.11	4.02	11.65	1620.44
194	81	1770.47	523.17	935.60	126.02	4.30	12.65	4.76	11.73	1618.23
195	85	1771.38	522.48	938.33	126.96	4.39	11.63	4.85	11.78	1620.42
196	355	1751.31	522.05	920.71	128.15	4.81	9.78	5.42	13.03	1603.95
197	450	1753.29	521.63	923.61	130.33	5.05	8.58	5.57	13.48	1608.25
198	460	1760.37	521.44	927.26	131.22	5.35	11.92	5.63	13.62	1616.44
199	556	1753.28	516.96	923.76	131.93	5.46	11.98	5.81	14.97	1610.87
200	580	1747.90	510.86	878.64	168.43	14.04	11.52	6.09	15.52	1605.10
201	665	1752.72	507.38	884.74	168.84	14.40	13.45	6.41	15.69	1610.91
202	775	1754.48	506.49	883.73	173.07	14.85	12.80	6.55	15.85	1613.34
203	880	1757.42	504.92	888.05	174.05	15.32	12.05	6.79	16.32	1617.50
204	3100	1739.75	497.13	877.34	177.30	16.68	11.19	7.37	16.91	1603.92
205	41	1767.68	560.67	614.13	118.75	3.61	10.82	3.99	9.13	1321.10
206	45	1773.35	551.79	928.83	119.29	3.69	9.70	4.08	10.05	1627.43
207	58	1771.46	560.81	915.07	120.14	3.78	12.08	4.25	10.34	1626.47
208	65	1780.90	560.93	923.78	121.09	3.95	11.50	4.46	10.43	1636.14
209	80	1782.29	495.87	989.37	122.00	3.98	12.18	4.59	10.67	1638.66
210	81	1776.48	551.20	927.71	122.32	4.25	11.83	4.62	10.72	1632.65
211	85	1767.39	559.68	909.35	123.06	4.35	11.29	4.66	10.78	1623.17
212	88	1764.51	555.09	909.27	123.92	4.38	12.78	4.73	10.87	1621.04
213	224	1760.21	543.74	914.89	124.10	4.71	11.03	5.47	13.35	1617.29
214	268	1759.55	540.71	917.19	126.08	4.82	9.65	5.52	13.44	1617.41
215	385	1757.35	529.60	921.86	126.27	5.00	13.17	5.71	13.93	1615.54
216	412	1748.51	527.39	914.41	126.66	5.24	12.28	5.88	14.28	1606.14
217	533	1742.00	522.91	909.66	128.44	5.39	12.06	6.08	15.05	1599.59

218	628	1742.31	506.56	878.22	168.24	14.39	12.01	6.28	15.21	1600.91
219	780	1734.57	505.70	869.83	169.19	14.91	12.61	6.50	15.56	1594.30
220	820	1731.25	500.94	867.99	170.07	15.72	12.50	6.59	15.68	1589.49
221	1900	1720.70	498.74	865.84	171.49	16.46	9.62	7.12	16.55	1585.82
222	36	1761.70	549.59	918.31	119.96	3.79	12.26	4.18	10.27	1618.36
223	40	1760.79	558.31	906.71	120.25	3.85	12.35	4.33	11.23	1617.03
224	45	1763.26	559.06	907.63	120.50	3.94	12.20	4.36	11.45	1619.14
225	58	1762.58	559.20	909.31	121.15	4.16	10.82	4.49	11.61	1620.74
226	62	1753.27	539.81	917.11	121.24	4.19	12.87	4.57	11.65	1611.44
227	70	1754.57	548.53	907.75	123.43	4.28	12.12	4.62	11.74	1612.47
228	80	1746.34	549.41	899.77	124.35	4.32	11.67	4.66	11.78	1605.96
229	280	1748.87	548.93	900.24	124.52	5.31	11.19	5.55	13.35	1609.09
230	402	1743.28	531.64	907.83	124.77	5.45	12.33	5.89	15.04	1602.95
231	460	1741.40	524.41	912.53	125.09	5.49	12.18	5.92	15.27	1600.89
232	520	1740.61	508.69	928.20	125.86	5.60	11.06	5.98	15.63	1601.02
233	550	1741.26	507.83	878.79	169.96	14.01	9.65	6.08	15.67	1601.99
234	630	1738.77	505.23	873.59	171.17	14.49	12.21	6.37	15.95	1599.01
235	720	1740.27	497.76	880.46	171.45	14.84	12.29	6.51	16.06	1599.37
236	820	1737.27	497.10	881.98	171.67	15.42	11.79	6.65	16.23	1600.84
237	840	1732.19	496.69	874.71	173.44	15.54	12.85	6.71	16.32	1596.26
238	1850	1724.91	495.95	864.15	177.82	16.40	11.27	7.27	16.64	1589.50
239	36	1782.28	549.48	940.84	117.25	2.42	11.29	3.83	10.48	1635.59
240	41	1781.36	558.20	928.39	118.19	3.71	12.59	4.02	11.37	1636.47
241	48	1780.44	558.95	921.99	121.28	3.39	12.88	4.29	11.63	1634.41
242	53	1773.29	559.16	912.58	121.76	3.92	14.05	4.36	12.29	1628.12

243	62	1772.80	539.70	933.10	122.56	4.19	11.78	4.49	12.45	1628.27
244	73	1772.18	548.42	923.41	123.44	4.34	11.45	4.58	12.66	1628.30
245	85	1771.51	549.17	919.72	124.35	4.38	11.11	4.65	12.94	1626.32
246	87	1770.27	549.31	920.15	125.03	4.50	9.78	4.70	13.47	1626.94
247	91	1770.04	546.69	923.39	126.16	4.56	8.57	4.87	13.63	1627.87
248	310	1757.19	529.07	922.85	126.65	5.09	9.82	5.76	15.12	1614.36
249	435	1759.27	525.96	921.52	129.10	5.32	11.96	5.93	15.64	1615.43
250	568	1756.76	514.63	882.68	170.13	14.58	11.92	6.25	15.67	1615.86
251	654	1754.54	505.57	887.40	171.07	14.85	12.75	6.59	16.04	1614.27
252	708	1752.67	502.44	888.97	171.79	15.16	10.87	6.71	16.15	1612.09
253	788	1752.25	502.20	885.75	173.00	15.45	12.09	6.86	16.48	1611.83
254	872	1750.52	497.73	886.64	176.16	15.49	12.16	6.99	16.52	1611.69
255	4150	1737.35	492.40	875.70	180.02	17.00	11.97	7.38	17.57	1602.04
256	55	1748.41	541.98	921.80	116.28	3.95	11.89	4.25	11.35	1611.50
257	63	1746.15	541.49	922.02	114.34	4.03	10.82	4.21	11.48	1608.39
258	102	1746.88	540.56	918.12	117.09	4.06	13.16	4.62	11.74	1609.35
259	116	1745.62	539.88	918.66	118.98	4.19	10.62	4.67	12.28	1609.28
260	146	1742.69	538.67	913.90	121.17	4.31	11.81	4.78	12.55	1607.19
261	187	1734.61	533.93	909.85	123.15	4.42	10.38	4.88	12.64	1599.25
262	233	1737.67	532.67	912.37	124.09	5.10	12.08	5.11	13.03	1604.45
263	413	1733.44	517.44	919.61	126.83	5.31	11.89	5.89	14.97	1601.94
264	685	1730.61	511.27	871.18	168.32	13.29	11.52	6.35	15.46	1597.39
265	1032	1717.30	511.40	852.09	171.16	15.42	11.71	7.01	16.04	1584.83
266	1148	1718.18	506.15	855.03	171.93	15.48	12.61	7.08	16.33	1584.61
267	1241	1714.24	510.86	846.97	174.56	15.55	11.33	7.19	16.48	1582.94

268	1289	1711.35	507.38	847.34	174.92	15.70	11.79	7.31	16.54	1580.98
269	1350	1701.28	506.49	836.65	175.47	15.78	11.20	7.36	16.62	1569.57
270	1475	1704.91	504.79	841.00	175.89	15.88	12.18	7.39	16.68	1573.81
271	26000	1688.52	494.13	838.04	177.16	16.81	10.96	7.65	17.05	1561.80
272	28000	1692.49	493.05	840.96	177.84	17.32	11.31	7.72	17.11	1565.31
273	36	1761.34	542.18	943.10	108.45	3.61	12.39	3.88	10.67	1624.28
274	40	1763.26	541.00	946.54	110.18	3.72	11.82	3.95	11.12	1628.33
275	50	1760.59	540.51	945.68	110.65	3.80	10.52	4.07	11.25	1626.48
276	58	1759.19	540.39	939.18	113.29	3.96	12.56	4.26	11.88	1625.52
277	63	1741.57	539.96	924.21	113.74	4.04	10.01	4.62	11.36	1607.94
278	66	1747.26	539.87	926.88	114.10	4.22	12.87	4.67	11.64	1614.25
279	70	1743.27	539.56	923.28	114.29	4.34	12.12	4.74	11.82	1610.15
280	75	1727.98	538.44	908.14	115.95	4.39	11.70	4.87	12.01	1595.50
281	81	1752.39	538.31	932.07	116.39	4.50	11.57	4.92	12.03	1619.79
282	225	1745.27	535.00	926.72	114.83	5.11	11.99	5.39	14.22	1613.26
283	260	1741.88	533.64	924.13	115.85	5.18	11.52	5.42	14.37	1610.11
284	291	1739.67	532.88	919.69	116.96	5.34	13.45	5.48	14.43	1608.23
285	348	1734.88	532.15	915.80	118.25	5.41	10.78	5.86	14.94	1603.19
286	365	1731.36	527.93	912.98	121.35	5.48	12.05	6.01	15.12	1600.92
287	510	1736.71	521.69	871.97	165.16	14.32	11.19	6.08	15.38	1605.79
288	750	1734.56	506.25	883.16	167.43	15.39	10.82	6.59	15.59	1605.23
289	920	1727.77	505.67	872.05	169.94	16.02	9.70	6.96	16.33	1596.67
290	2650	1722.05	500.60	869.25	172.90	17.40	12.08	7.12	17.06	1596.41
291	40	1737.24	548.41	905.61	113.45	4.02	11.50	3.72	10.65	1597.36
292	45	1738.91	546.50	909.81	114.32	4.15	12.18	3.81	11.23	1602.00

293	55	1741.34	544.50	913.34	116.14	4.19	10.59	4.03	11.49	1604.28
294	62	1743.28	543.07	913.22	117.22	3.91	11.29	4.88	11.88	1605.47
295	64	1741.68	541.68	908.40	118.94	4.14	12.78	5.13	12.04	1603.11
296	70	1744.28	538.71	914.25	119.65	4.46	11.03	5.12	12.38	1605.60
297	80	1745.05	538.04	915.96	120.46	4.48	9.65	5.13	12.91	1606.63
298	201	1758.05	516.58	944.94	121.15	5.14	13.17	5.62	14.67	1621.27
299	240	1740.28	515.77	929.16	121.47	5.25	11.48	5.69	15.04	1603.86
300	300	1737.01	508.68	930.27	123.36	5.28	12.06	5.87	15.35	1600.87
301	367	1742.57	507.05	936.30	125.02	5.49	12.01	5.95	15.58	1607.40
302	382	1741.74	505.29	936.70	126.98	5.68	10.05	6.06	15.62	1606.38
303	478	1740.58	504.48	933.03	129.03	5.80	11.33	6.11	15.91	1605.69
304	536	1737.35	503.99	887.62	163.46	13.45	11.79	6.31	16.06	1602.68
305	750	1738.29	503.46	885.03	166.95	14.33	11.99	6.49	16.25	1604.50
306	1700	1730.41	503.37	871.88	170.05	15.82	11.92	6.61	16.63	1596.28
307	1900	1732.50	503.10	872.73	173.44	16.60	11.26	6.67	16.74	1600.54
308	2200	1726.24	502.75	866.84	176.29	17.11	12.08	6.78	16.79	1598.64
309	2500	1725.49	502.64	866.89	177.07	17.26	10.72	6.99	16.95	1598.52
310	52	1746.98	546.55	919.41	112.05	3.90	11.61	4.03	11.15	1608.70
311	55	1743.88	544.80	914.80	113.48	4.00	11.03	4.39	11.27	1603.77
312	65	1748.89	542.58	920.51	113.86	4.06	12.58	4.51	11.62	1609.72
313	70	1751.87	541.11	924.52	114.99	4.08	12.19	4.57	11.95	1613.41
314	70	1747.27	539.24	922.23	115.56	3.89	12.28	4.59	12.06	1609.85
315	135	1746.99	529.77	931.23	115.67	4.25	11.11	4.96	13.32	1610.31
316	240	1742.80	526.96	925.44	116.18	4.95	11.92	5.11	14.94	1605.50
317	320	1734.61	522.85	920.14	116.89	5.31	12.09	5.45	15.65	1598.38

318	400	1735.98	515.07	927.92	118.04	5.39	11.99	5.56	15.91	1599.88
319	450	1734.87	511.63	929.33	118.36	5.52	11.98	5.62	15.96	1598.40
320	480	1731.99	506.49	931.39	118.92	5.65	12.19	5.92	16.03	1596.59
321	650	1730.90	505.37	871.36	170.13	14.43	11.59	6.11	16.34	1595.33
322	1100	1727.68	503.10	872.47	172.23	15.44	11.19	6.59	16.65	1597.67
323	1150	1728.06	496.54	877.69	173.15	15.78	12.16	6.61	16.74	1598.67
324	1200	1726.90	491.78	881.59	173.65	15.90	12.26	6.67	16.79	1598.64
325	1300	1723.17	503.87	866.81	174.03	16.09	12.00	6.78	16.86	1596.44
326	1800	1720.88	504.28	862.69	174.45	16.21	12.18	6.99	17.47	1594.27
327	35	1793.38	538.91	961.92	114.48	3.71	11.83	4.03	11.54	1646.42
328	37	1791.21	538.07	959.94	114.58	3.76	11.29	4.11	11.65	1643.40
329	40	1778.49	536.99	949.28	114.66	3.14	12.78	4.37	11.68	1632.90
330	50	1777.36	542.48	940.01	117.23	4.40	11.03	4.46	12.33	1631.94
331	55	1773.77	542.06	937.42	117.92	4.49	9.65	4.78	12.49	1628.81
332	120	1772.67	541.50	933.55	118.47	3.33	13.17	4.91	13.32	1628.25
333	150	1770.49	541.01	933.36	120.06	4.44	8.51	4.93	13.64	1625.95
334	180	1768.88	539.63	929.13	120.95	4.25	12.06	4.96	14.03	1625.01
335	220	1767.25	539.50	926.78	121.37	4.49	12.01	5.12	14.91	1624.18
336	260	1763.46	532.85	929.66	122.18	5.14	9.89	5.38	15.55	1620.65
337	350	1762.88	526.74	931.66	123.22	5.51	11.33	5.49	15.63	1619.58
338	400	1763.39	525.10	931.90	125.44	5.68	11.79	5.92	15.85	1621.68
339	500	1764.51	522.85	885.74	166.19	13.93	11.20	6.47	15.96	1622.34
340	550	1762.15	518.69	884.19	169.15	14.31	12.18	6.52	16.18	1621.22
341	700	1740.87	518.08	860.51	172.26	14.46	11.99	6.60	16.45	1600.35
342	730	1739.78	517.21	859.88	173.84	14.71	10.31	6.65	16.52	1599.12

343	1250	1737.34	506.05	867.03	175.49	15.44	12.39	6.99	17.13	1600.52
344	35	1771.31	537.58	948.14	109.95	4.46	11.82	3.75	10.75	1626.45
345	50	1776.54	535.40	953.61	110.30	4.72	11.78	4.03	10.94	1630.78
346	52	1778.26	535.07	952.68	111.46	5.41	11.26	4.06	11.15	1631.09
347	150	1786.60	526.79	971.46	111.63	5.49	10.01	4.57	12.22	1642.17
348	180	1779.65	526.28	964.30	112.28	3.81	12.09	4.66	12.45	1635.87
349	210	1773.56	523.65	959.99	112.85	3.56	11.12	5.01	14.07	1630.25
350	240	1774.65	523.44	957.45	113.94	4.94	11.98	5.07	14.26	1631.08
351	280	1772.95	522.87	953.35	115.47	5.81	12.70	5.25	14.38	1629.83
352	350	1770.85	520.61	956.13	116.06	6.03	10.01	5.49	15.13	1629.46
353	380	1769.51	512.48	962.68	116.30	5.32	11.88	5.68	15.56	1629.90
354	450	1770.26	510.45	963.75	117.12	6.40	11.28	5.86	15.62	1630.48
355	530	1746.67	506.69	888.04	164.37	13.79	12.23	6.08	15.75	1606.95
356	760	1748.85	504.81	892.19	166.39	14.41	10.92	6.46	15.84	1611.02
357	1180	1747.26	497.68	894.11	168.24	14.91	12.09	6.68	16.08	1609.79
358	1200	1742.44	496.05	887.61	171.13	15.14	12.70	6.76	16.16	1605.55
359	22000	1737.58	491.93	875.96	177.82	17.46	11.19	7.37	22.55	1604.28
360	38	1776.54	535.17	935.06	111.18	3.75	12.16	4.25	11.65	1613.22
361	40	1767.86	531.70	932.41	111.49	3.84	12.26	4.46	11.74	1607.90
362	45	1756.65	530.83	920.77	111.55	3.93	12.00	4.53	12.11	1595.72
363	50	1760.26	530.36	926.84	112.13	4.02	12.18	4.57	12.37	1602.47
364	56	1753.10	529.95	914.19	112.40	4.10	11.83	4.61	12.54	1589.62
365	62	1756.94	529.17	917.10	112.73	4.21	11.29	4.72	12.62	1591.84
366	75	1757.31	527.44	917.66	113.50	4.34	12.40	4.73	13.05	1593.12
367	81	1752.42	523.95	917.59	113.95	4.43	11.03	4.81	13.83	1589.59

368	86	1750.20	520.73	916.18	114.94	4.54	9.65	4.86	14.34	1585.24
369	432	1753.49	516.96	909.45	115.87	5.41	13.17	5.92	16.21	1582.99
370	540	1761.26	511.67	859.62	168.16	14.31	12.28	5.98	16.35	1588.37
371	650	1768.69	506.36	870.35	168.85	14.49	12.06	6.09	16.66	1594.86
372	780	1753.14	501.50	856.56	169.97	14.80	12.01	6.37	17.04	1578.25
373	850	1784.77	496.61	891.30	171.56	15.42	10.71	6.78	17.18	1609.56
374	4600	1803.49	493.45	901.78	176.09	16.71	11.33	7.02	20.09	1626.47
375	32	1760.24	544.69	916.50	112.13	3.29	11.79	4.69	12.65	1605.74
376	38	1758.57	543.91	913.79	112.47	4.25	11.20	4.72	13.47	1603.81
377	42	1755.17	540.44	907.50	113.38	3.52	11.49	4.73	14.15	1595.21
378	49	1752.58	540.00	902.16	113.96	5.00	10.96	4.76	14.44	1591.28
379	52	1751.41	539.45	900.07	114.32	4.39	10.31	4.78	15.22	1588.54
380	60	1747.27	537.80	893.47	114.43	4.62	12.39	4.79	15.63	1583.13
381	65	1743.11	536.96	889.46	114.66	4.69	11.82	4.82	16.08	1578.49
382	73	1741.32	536.10	889.28	114.69	4.95	11.97	4.88	16.36	1578.23
383	80	1745.46	531.63	896.82	115.17	5.06	11.26	4.95	16.63	1581.52
384	145	1752.57	528.14	905.67	115.49	5.18	10.01	5.07	16.92	1586.48
385	285	1760.94	518.91	914.84	119.18	5.78	12.09	5.21	17.15	1593.16
386	560	1757.40	516.55	854.91	167.72	13.81	12.42	5.68	17.24	1588.33
387	650	1764.32	515.13	858.98	168.15	14.46	11.98	5.86	17.33	1591.89
388	750	1768.19	509.39	867.71	168.94	14.72	9.92	6.02	17.56	1594.26
389	835	1775.46	504.05	874.70	170.30	15.34	12.01	6.09	18.18	1600.67
390	2700	1791.97	496.87	885.96	174.16	16.10	12.68	6.72	20.42	1612.91
391	40	1672.38	528.85	842.79	113.27	5.19	12.12	4.37	11.37	1517.96
392	45	1683.29	528.46	850.76	113.48	4.42	12.49	4.49	12.21	1526.31

393	47	1695.68	526.99	858.94	114.00	4.91	12.38	4.53	12.64	1534.39
394	52	1710.30	526.47	874.96	114.08	5.09	11.82	4.61	13.43	1550.46
395	62	1716.72	526.07	881.54	115.43	5.35	10.52	4.66	14.02	1557.59
396	67	1724.87	525.64	885.73	115.77	5.50	10.01	4.77	15.58	1563.00
397	75	1735.40	525.39	891.19	116.40	5.71	11.88	4.98	16.22	1571.77
398	79	1742.92	523.88	899.52	117.18	5.79	11.28	5.05	17.03	1579.73
399	160	1746.17	518.96	901.22	117.82	6.15	12.11	5.47	18.34	1580.07
400	220	1750.31	516.20	904.09	120.15	6.00	10.92	5.89	19.17	1582.42
401	400	1755.64	506.87	910.32	124.48	6.44	12.09	6.26	20.35	1586.81
402	550	1757.72	504.44	864.55	164.40	14.44	11.99	6.57	21.16	1587.55
403	630	1760.20	499.09	868.39	167.46	14.68	11.19	6.69	21.32	1588.82
404	710	1762.10	497.91	867.68	168.95	15.25	12.16	6.76	21.55	1590.26
405	750	1763.28	497.76	868.35	169.85	15.80	12.26	6.89	21.66	1592.57
406	880	1768.66	495.74	870.07	173.04	15.92	12.00	6.99	21.94	1595.70
407	3750	1783.57	493.59	879.14	175.88	16.98	12.18	7.55	23.43	1608.75
408	33	1698.60	541.83	850.35	114.86	4.69	11.83	3.94	11.27	1538.77
409	36	1705.27	539.57	857.68	115.26	4.93	11.29	3.97	11.65	1544.35
410	40	1707.87	537.41	862.14	115.44	5.44	10.60	4.05	12.32	1547.40
411	42	1710.94	537.29	864.91	115.66	4.06	11.03	4.09	12.54	1549.58
412	45	1712.27	537.23	868.85	116.12	4.84	9.65	4.13	13.16	1553.98
413	50	1714.68	534.15	868.39	116.93	3.81	11.03	4.39	13.52	1552.22
414	55	1717.95	533.63	872.78	117.08	3.50	12.28	4.47	14.21	1557.95
415	60	1725.66	533.33	847.32	117.46	5.49	12.06	4.56	44.68	1564.90
416	73	1726.32	520.06	886.56	118.95	5.42	12.01	4.71	15.34	1563.05
417	105	1730.27	519.48	895.64	120.07	5.04	8.61	4.92	16.03	1569.79

418	115	1731.37	518.23	892.70	121.19	4.89	11.33	4.93	16.69	1569.96
419	120	1734.68	516.51	892.13	124.32	4.56	11.79	4.98	17.32	1571.61
420	122	1738.11	512.36	896.69	125.95	4.60	11.20	5.01	17.54	1573.35
421	135	1742.20	510.55	899.69	126.08	5.05	12.18	5.03	17.66	1576.24
422	160	1747.45	509.06	906.31	127.16	6.06	10.96	5.46	18.05	1583.06
423	265	1749.01	506.09	906.48	127.89	6.41	10.31	5.71	19.13	1582.02
424	35	1627.77	527.63	819.12	128.16	4.46	12.07	3.75	13.46	1508.65
425	36	1630.94	527.60	840.99	107.29	4.69	11.82	3.76	13.58	1509.73
426	40	1632.37	523.48	839.72	111.48	4.32	12.58	3.82	14.01	1509.41
427	45	1633.47	522.65	842.90	112.17	4.03	11.26	3.86	14.42	1511.29
428	48	1634.77	519.18	846.94	112.74	4.40	10.01	3.91	14.55	1511.73
429	55	1629.32	514.60	843.42	113.00	3.86	12.09	4.09	14.66	1505.72
430	60	1627.31	513.74	842.96	113.19	4.84	12.09	4.27	14.93	1506.02
431	70	1642.46	512.85	857.54	113.54	4.90	11.98	4.49	15.07	1520.37
432	75	1631.97	512.46	848.76	113.65	4.36	10.77	4.56	15.25	1509.81
433	80	1630.30	512.34	844.41	114.15	3.73	12.01	4.86	15.32	1506.82
434	88	1635.10	512.24	850.70	114.87	3.42	11.70	4.92	15.48	1513.33
435	93	1628.37	509.55	843.12	115.19	4.25	12.12	5.08	15.55	1504.86
436	150	1642.49	506.91	855.79	115.85	4.51	11.96	5.37	16.01	1516.40
437	370	1649.85	506.17	858.69	116.96	4.79	10.87	5.89	16.09	1519.46
438	450	1653.56	501.09	861.00	118.12	5.05	12.52	5.98	16.53	1520.29
439	40	1674.57	529.64	866.72	107.83	3.90	11.50	4.21	16.66	1540.46
440	45	1660.57	529.43	855.04	108.26	4.05	11.99	4.25	16.35	1529.37
441	50	1664.41	529.00	856.83	108.45	4.29	12.08	4.38	16.42	1531.45
442	56	1675.55	527.38	868.88	111.02	4.26	11.47	4.52	16.18	1543.71

443	63	1682.52	526.05	875.73	111.30	4.10	12.29	4.59	18.29	1552.35
444	72	1661.39	524.74	853.31	111.87	3.75	11.92	4.78	19.03	1529.40
445	88	1677.98	522.88	870.28	112.89	5.04	11.98	4.86	19.27	1547.20
446	91	1693.44	520.96	886.85	114.35	4.41	11.86	5.23	19.35	1563.01
447	200	1695.59	508.69	892.80	115.85	5.42	11.09	5.57	21.21	1560.63
448	230	1698.57	508.09	894.16	116.74	5.45	12.11	5.66	22.34	1564.55
449	560	1705.27	501.61	848.33	162.16	14.59	11.98	6.01	23.67	1568.35
450	920	1730.95	498.47	866.54	168.44	15.70	12.16	6.67	23.15	1591.13
451	3500	1758.36	493.45	884.96	176.03	16.82	11.50	7.03	23.99	1613.78
452	40	1627.78	537.17	833.94	107.88	3.59	11.82	4.52	11.63	1510.55
453	45	1630.84	536.04	834.65	108.19	3.93	12.19	4.66	12.07	1511.73
454	50	1643.46	532.75	847.48	108.92	4.44	9.80	4.73	11.45	1519.57
455	110	1661.70	531.40	859.42	115.10	4.01	11.99	5.57	10.85	1538.34
456	120	1650.27	527.44	851.37	115.47	4.46	11.89	5.66	10.74	1527.03
457	130	1671.38	525.40	872.05	115.64	4.30	11.52	5.69	11.36	1545.96
458	200	1659.75	514.83	863.86	118.12	4.95	11.88	5.86	12.15	1531.65
459	230	1664.37	513.85	868.17	118.72	5.19	12.65	5.92	13.48	1537.98
460	301	1672.68	512.47	875.01	123.46	5.44	12.06	5.99	13.94	1548.37
461	1050	1717.01	511.40	860.89	166.52	16.53	9.75	6.67	15.25	1587.01
462	1120	1723.66	506.15	870.06	168.04	16.78	10.93	6.71	15.75	1594.42
463	1180	1728.85	510.86	866.67	170.16	16.91	11.69	6.78	16.15	1599.22
464	1250	1739.37	507.38	878.63	171.07	16.99	11.19	6.83	16.52	1608.61
465	1270	1743.57	506.49	882.61	171.87	17.52	11.62	6.89	17.23	1614.23
466	1360	1746.98	495.95	889.38	173.49	17.54	12.78	6.96	17.87	1613.97
467	19000	1760.98	494.13	886.17	176.75	17.63	11.72	7.36	31.34	1625.10

468	32	1793.39	535.64	963.70	125.94	4.02	11.93	3.51	11.45	1656.19
469	45	1789.69	533.74	962.94	126.15	4.24	11.70	3.99	12.67	1655.43
470	65	1783.67	531.71	961.16	126.30	3.86	11.81	4.11	13.39	1652.34
471	75	1790.38	527.60	967.11	126.32	4.58	11.99	4.38	14.23	1656.21
472	130	1788.44	526.89	967.90	126.85	3.44	11.52	4.62	15.12	1656.34
473	180	1778.44	525.77	952.42	126.97	4.33	13.25	4.95	15.95	1643.64
474	240	1767.71	512.63	952.10	127.03	5.11	12.80	5.38	16.24	1631.29
475	260	1779.35	512.07	964.71	127.28	5.35	12.29	5.49	16.62	1643.81
476	350	1771.90	508.63	958.00	127.65	5.49	11.19	5.86	17.85	1634.67
477	360	1775.86	507.65	962.55	128.72	5.56	10.82	5.91	18.37	1639.58
478	470	1786.65	506.10	971.38	130.10	5.62	10.59	6.09	18.66	1648.54
479	520	1773.40	505.36	912.38	166.49	12.34	12.08	6.27	19.82	1634.74
480	685	1777.28	503.84	911.56	167.72	14.43	11.50	6.53	20.28	1635.86
481	1150	1790.21	500.47	912.05	170.15	15.82	12.18	6.92	21.33	1638.92
482	1300	1792.45	498.97	912.96	172.26	16.03	11.83	6.98	21.64	1640.67
483	1850	1782.58	497.33	901.62	172.97	16.52	11.29	7.02	22.21	1628.96
484	41	1775.57	529.61	940.53	122.95	4.10	11.92	7.55	12.05	1628.71
485	45	1777.69	529.09	949.21	123.47	4.26	11.03	4.17	12.58	1633.81
486	62	1773.50	527.68	947.59	124.32	4.28	11.45	4.49	13.45	1633.26
487	75	1772.25	527.36	941.06	124.53	4.42	13.17	4.76	13.62	1628.92
488	120	1770.81	526.00	940.60	126.29	3.81	12.28	4.91	15.15	1629.04
489	125	1741.28	525.57	909.66	126.44	4.48	12.06	4.98	15.28	1598.47
490	136	1774.44	523.96	942.97	127.09	4.23	11.52	5.05	15.52	1630.34
491	210	1775.54	521.57	943.56	127.12	4.40	12.61	5.59	15.93	1630.78
492	280	1773.98	517.69	946.43	127.50	4.91	11.33	5.77	16.45	1630.08

493	350	1770.81	519.07	938.56	128.02	5.14	11.48	5.71	17.04	1625.02
494	370	1774.65	516.86	941.28	128.94	5.29	11.92	5.88	17.47	1627.64
495	440	1779.37	512.63	948.82	130.45	5.50	12.09	6.03	18.03	1633.55
496	530	1782.57	510.48	906.15	165.63	15.40	11.56	6.39	18.94	1634.55
497	680	1783.42	505.46	909.67	167.36	15.61	11.11	6.49	20.21	1635.91
498	740	1778.72	503.45	900.34	168.84	15.99	11.83	6.78	21.38	1628.61
499	1250	1788.44	501.50	906.07	171.12	17.11	12.55	6.98	22.27	1637.60
500	1440	1795.60	498.77	913.39	172.26	17.39	11.78	6.96	23.45	1644.00
501	1800	1786.72	497.99	901.93	173.05	17.48	10.80	7.13	25.63	1634.01
502	35	1759.31	525.58	960.55	105.96	3.85	11.08	3.83	13.35	1624.20
503	65	1760.39	522.69	960.77	106.74	3.93	12.22	4.01	14.15	1624.51
504	122	1758.00	519.74	953.26	111.17	3.98	11.82	4.38	15.55	1619.90
505	126	1763.07	519.08	958.93	112.48	4.12	12.00	4.63	15.63	1626.87
506	130	1765.36	518.49	960.22	112.83	4.16	11.09	4.99	15.75	1627.53
507	130	1772.77	518.17	966.72	113.96	4.21	10.03	5.07	15.78	1633.94
508	210	1770.68	516.25	961.60	115.50	4.44	11.59	5.67	16.45	1631.50
509	270	1771.30	513.97	961.13	116.82	4.99	12.36	5.78	16.62	1631.67
510	360	1772.60	512.71	957.21	121.15	5.43	12.11	5.82	17.13	1631.56
511	425	1772.98	510.54	955.07	127.33	5.48	10.91	5.97	17.37	1632.67
512	500	1773.40	506.90	916.39	160.48	13.16	11.75	6.12	17.92	1632.72
513	750	1774.55	505.29	907.87	167.06	13.60	11.72	5.48	19.31	1630.33
514	1023	1783.19	503.95	909.20	169.16	14.81	12.21	6.72	20.25	1636.30
515	1069	1786.78	502.48	912.69	169.97	14.85	10.81	6.88	21.22	1638.90
516	1102	1797.20	500.77	920.76	171.49	14.86	10.89	6.92	21.64	1647.33
517	1120	1803.72	498.39	932.10	173.45	14.91	9.70	6.96	22.04	1657.55

518	1250	1782.90	496.04	909.15	174.06	15.04	12.13	7.06	24.56	1638.04
519	40	1772.58	535.48	960.54	105.27	3.75	11.99	3.88	14.17	1635.08
520	42	1771.65	534.70	959.72	105.73	3.81	10.87	4.11	15.28	1634.22
521	47	1767.84	532.58	957.76	107.15	3.89	11.61	4.38	16.13	1633.50
522	50	1770.76	532.16	960.77	108.49	4.01	12.35	4.53	16.32	1638.63
523	51	1768.51	528.64	960.28	108.57	4.09	11.97	4.69	16.48	1634.72
524	55	1764.41	525.06	955.73	110.26	4.24	12.11	4.88	17.35	1629.63
525	60	1767.08	519.45	961.47	111.13	4.28	12.00	5.08	18.25	1631.66
526	71	1753.46	516.59	948.49	112.48	4.33	11.52	5.26	19.13	1617.80
527	110	1764.47	514.10	958.10	114.50	4.40	11.09	5.52	21.26	1628.97
528	116	1771.71	510.41	964.54	116.56	4.14	11.70	5.86	21.64	1634.85
529	118	1770.57	509.89	955.88	122.27	4.48	12.06	5.98	21.71	1632.27
530	350	1773.44	506.45	958.43	123.17	5.08	11.91	6.29	23.37	1634.70
531	435	1774.58	505.33	955.08	128.44	5.19	10.83	6.52	24.26	1635.65
532	468	1774.89	502.96	953.25	128.72	5.42	12.10	6.63	25.18	1634.26
533	594	1778.77	502.07	911.81	164.39	14.44	11.79	6.67	25.94	1637.11
534	750	1776.66	499.45	904.87	167.46	14.91	12.11	6.81	27.45	1633.06
535	1250	1789.90	498.09	908.63	172.85	16.50	11.93	6.96	29.07	1644.03
536	32	1771.58	526.35	968.08	106.59	3.70	11.82	3.89	11.25	1631.68
537	38	1764.68	525.69	964.66	106.82	3.83	11.47	3.96	11.38	1627.81
538	45	1762.87	525.55	960.39	107.92	3.95	12.08	4.08	11.47	1625.44
539	52	1756.42	525.26	954.25	108.46	3.99	11.98	4.12	12.12	1620.18
540	53	1760.24	523.94	958.53	108.63	4.09	12.00	4.39	12.54	1624.12
541	72	1756.55	523.58	952.43	110.03	4.25	12.02	4.98	13.05	1620.34
542	130	1761.40	521.81	953.57	113.56	4.31	12.16	5.05	14.23	1624.69

543	170	1765.61	518.48	959.45	114.89	4.40	10.82	5.11	14.95	1628.10
544	240	1767.58	514.97	957.67	117.26	4.95	12.27	5.13	17.04	1629.29
545	310	1771.66	509.06	964.89	119.95	5.31	11.48	5.41	17.16	1633.26
546	360	1770.75	505.44	962.57	123.44	5.42	12.09	5.61	17.55	1632.12
547	390	1764.50	501.76	957.40	124.50	5.54	12.29	5.72	17.62	1624.83
548	500	1771.55	497.53	915.23	166.82	14.61	12.35	5.87	18.91	1631.32
549	710	1775.26	496.01	914.24	168.83	15.71	13.28	6.05	19.28	1633.40
550	760	1777.68	495.46	918.16	170.15	16.09	9.09	6.09	20.23	1635.27
551	1450	1783.91	492.00	917.84	174.49	16.81	9.72	6.81	21.47	1639.14
552	37	1741.72	532.38	935.10	105.46	3.71	11.98	4.59	11.27	1604.49
553	45	1749.40	530.71	942.19	106.26	3.82	12.06	4.76	11.48	1611.28
554	51	1754.27	530.48	947.08	106.89	3.86	9.57	4.85	12.34	1615.07
555	56	1754.99	527.53	948.40	108.06	3.88	10.12	4.91	12.65	1615.55
556	64	1757.39	523.96	951.75	108.99	4.14	10.91	4.93	12.92	1617.60
557	75	1761.14	519.50	956.66	109.88	4.18	11.50	4.97	13.57	1620.26
558	155	1762.35	508.76	964.90	111.29	4.35	11.99	5.65	14.08	1621.02
559	265	1765.88	505.86	964.78	117.13	4.41	11.06	5.71	15.13	1624.08
560	382	1771.10	504.60	967.12	120.49	4.83	10.73	5.82	15.27	1628.86
561	425	1763.57	503.97	953.63	126.23	5.05	10.23	5.86	15.92	1620.89
562	450	1767.65	503.48	954.04	126.96	5.42	12.10	5.98	16.25	1624.23
563	530	1774.25	503.10	912.28	166.39	14.10	12.16	6.11	16.49	1630.63
564	580	1773.48	501.89	911.61	166.88	14.49	11.99	6.36	16.53	1629.75
565	710	1778.98	500.59	915.35	168.16	15.28	11.61	6.59	16.61	1634.19
566	750	1779.47	500.51	913.67	168.63	15.46	12.29	6.67	16.65	1633.88
567	1089	1785.69	498.24	915.06	171.10	15.90	12.65	6.96	16.85	1636.76

568	46	1604.91	523.38	820.13	107.72	3.78	11.27	4.46	12.04	1482.78
569	55	1605.57	520.68	823.37	107.84	3.86	11.78	4.62	12.32	1484.47
570	58	1603.32	519.15	821.96	108.20	3.89	10.22	4.68	12.64	1480.74
571	60	1607.51	516.06	827.67	110.48	3.98	9.81	4.73	13.09	1485.82
572	115	1608.45	508.70	832.58	110.53	4.44	10.92	5.51	14.27	1486.95
573	160	1613.35	506.33	837.38	110.55	4.46	11.77	5.59	14.38	1490.46
574	175	1614.55	506.11	836.92	112.17	4.71	11.99	5.91	14.54	1492.35
575	188	1616.71	505.93	838.22	112.47	4.80	11.52	5.96	14.63	1493.53
576	192	1617.29	505.35	837.06	112.96	4.92	11.71	6.02	15.01	1493.03
577	215	1623.35	468.40	878.04	114.48	5.31	10.72	6.28	15.35	1498.58
578	236	1624.41	503.54	841.87	114.87	5.39	11.33	6.35	15.47	1498.82
579	275	1622.27	503.07	835.71	117.15	5.56	11.79	6.37	15.55	1495.20
580	280	1624.51	501.59	839.05	118.15	5.72	11.20	6.41	15.67	1497.79
581	293	1625.05	501.30	834.04	121.39	5.78	12.18	6.61	16.06	1497.36
582	1240	1649.40	497.55	800.40	167.86	15.45	10.96	6.97	16.68	1515.87
583	1350	1647.87	497.49	797.47	168.19	15.79	11.31	7.08	16.92	1514.25
584	1400	1652.26	497.37	795.78	170.26	16.42	12.09	7.19	17.05	1516.16
585	1510	1653.57	496.99	792.97	173.44	17.70	11.82	7.27	17.14	1517.33
586	35	1639.58	537.87	834.70	113.06	3.60	10.52	4.27	12.67	1516.69
587	38	1641.34	534.69	835.55	114.56	3.71	12.56	4.36	12.71	1518.14
588	43	1639.45	527.85	840.23	114.93	3.79	10.56	4.62	13.05	1515.03
589	113	1659.55	523.98	860.15	116.15	4.11	12.87	4.67	13.18	1535.11
590	168	1660.39	520.98	863.36	116.83	4.19	12.12	4.81	13.56	1535.85
591	175	1663.46	519.45	864.36	117.29	4.44	11.70	4.99	14.12	1536.35
592	220	1665.60	510.10	871.97	118.15	4.81	11.57	5.31	14.45	1536.36

593	240	1670.77	508.93	873.29	120.94	4.92	11.99	5.46	15.21	1540.74
594	265	1671.58	508.17	871.80	123.48	5.04	11.52	5.51	15.64	1541.16
595	271	1671.77	506.85	870.40	125.02	5.25	11.49	5.63	16.33	1540.97
596	350	1642.11	506.46	838.71	126.43	5.46	10.78	5.71	16.59	1510.14
597	1023	1684.34	506.01	824.93	167.56	15.98	12.05	5.98	16.89	1549.40
598	1065	1685.49	503.59	826.67	169.17	16.15	11.19	6.03	16.91	1549.71
599	1132	1685.91	502.78	825.38	170.84	16.43	10.82	6.26	16.97	1549.48
600	1150	1686.54	500.51	824.45	173.46	16.50	11.47	6.42	17.05	1549.86
601	12000	1773.92	494.00	892.24	178.75	18.42	12.08	7.29	20.93	1623.71
602	38	1630.61	532.11	840.25	101.84	3.79	11.50	4.59	12.67	1506.75
603	45	1631.16	527.99	842.34	102.97	3.85	12.18	4.66	12.74	1506.73
604	52	1632.44	524.86	847.09	103.48	3.91	10.59	4.52	13.32	1507.77
605	70	1633.25	521.05	849.93	103.55	3.92	11.29	4.76	13.66	1508.16
606	120	1634.51	519.47	852.69	103.79	3.99	9.82	4.99	14.53	1509.28
607	128	1634.98	514.96	853.68	105.25	4.08	11.03	5.25	14.77	1509.02
608	133	1635.07	511.67	855.35	105.49	4.16	11.60	5.42	15.14	1508.83
609	140	1635.30	508.43	858.02	105.86	4.24	11.47	5.67	15.35	1509.04
610	146	1636.18	506.01	860.40	106.20	4.30	11.48	5.77	15.42	1509.58
611	152	1637.38	505.55	860.27	106.92	4.39	12.06	5.78	15.58	1510.55
612	158	1637.91	504.90	858.48	109.05	4.45	12.01	5.85	15.95	1510.69
613	166	1638.74	502.76	854.80	114.48	4.49	11.45	5.92	16.04	1509.94
614	180	1638.27	500.50	851.57	118.16	4.51	11.33	5.99	16.47	1508.53
615	210	1637.84	498.69	846.26	122.34	5.24	11.79	6.08	17.13	1507.53
616	1100	1669.85	496.44	814.02	167.82	17.14	11.99	6.71	18.55	1532.67
617	1120	1671.47	496.11	812.91	168.76	17.21	11.92	6.86	19.31	1533.08

618	16000	1768.90	491.79	890.91	178.04	17.79	11.26	7.36	22.23	1619.38
619	40	1613.41	538.09	815.95	107.92	3.36	10.72	3.88	11.58	1491.50
620	43	1614.68	535.63	817.82	108.29	3.48	10.72	4.07	11.95	1491.96
621	120	1617.86	529.77	821.44	110.06	4.11	11.61	4.46	12.27	1493.72
622	130	1618.32	518.97	830.67	111.18	4.20	11.03	4.82	13.06	1493.93
623	135	1619.36	513.57	833.43	113.08	4.29	12.11	4.95	13.47	1494.90
624	150	1622.20	511.69	836.83	113.89	4.35	12.19	5.03	13.62	1497.60
625	170	1622.92	510.57	837.17	114.30	4.38	12.28	5.27	13.93	1497.90
626	1032	1660.27	508.17	800.26	166.60	16.42	11.38	6.69	15.65	1525.17
627	1069	1663.45	506.35	803.26	167.48	16.51	11.92	6.78	15.92	1528.22
628	1075	1665.58	505.27	803.53	168.96	17.19	12.09	6.85	16.01	1529.90
629	1098	1666.40	503.94	804.49	169.13	17.68	11.99	6.92	16.08	1530.23
630	1150	1667.37	501.48	806.74	170.29	17.72	11.60	6.99	16.12	1530.94
631	1178	1668.80	499.80	807.58	171.15	18.05	12.19	7.12	16.25	1532.14
632	1185	1669.87	497.35	810.97	171.22	18.10	11.59	7.18	16.65	1533.06
633	23000	1754.89	492.33	877.42	177.85	18.71	11.19	7.67	21.41	1606.58
634	37	1610.80	529.83	818.11	107.16	2.95	12.16	4.22	11.54	1485.97
635	41	1611.57	528.47	817.98	107.96	3.11	12.11	4.49	12.25	1486.37
636	45	1614.68	525.55	821.41	109.06	4.19	12.00	4.56	12.57	1489.34
637	48	1615.24	523.94	821.87	109.98	3.88	12.18	4.71	13.01	1489.57
638	52	1615.89	522.04	821.72	110.46	4.36	11.83	4.83	13.42	1488.66
639	55	1616.66	519.16	825.76	110.67	3.84	11.29	4.88	13.66	1489.26
640	65	1617.35	514.60	830.02	111.28	3.88	10.70	4.97	13.93	1489.38
641	140	1618.24	507.01	836.57	111.45	3.90	11.03	5.49	14.68	1490.13
642	180	1616.80	503.55	835.92	112.13	4.71	11.52	5.61	15.14	1488.58

643	220	1620.28	503.43	835.50	112.44	4.81	13.17	5.96	15.84	1491.15
644	240	1621.39	503.36	833.51	113.53	5.15	13.75	6.11	16.01	1491.42
645	246	1621.87	503.25	834.61	113.75	4.52	12.06	6.17	16.25	1490.61
646	255	1622.50	503.07	835.15	114.33	3.36	12.01	6.39	16.32	1490.63
647	263	1622.72	502.84	831.44	114.82	4.94	12.65	6.48	16.49	1489.66
648	267	1622.85	502.57	831.76	116.06	4.82	11.33	6.52	16.53	1489.59
649	288	1623.26	502.40	830.91	117.14	4.29	11.79	6.61	16.64	1489.78
650	295	1624.39	502.06	828.91	119.23	5.35	11.20	6.72	16.68	1490.15
651	575	1627.77	501.21	772.58	165.19	17.04	12.18	6.98	16.77	1491.95
652	36	1626.24	524.03	843.45	108.19	3.88	11.99	4.45	10.25	1506.24
653	38	1627.41	521.60	849.45	108.53	4.40	10.31	4.58	9.63	1508.50
654	45	1627.70	520.96	851.89	108.95	2.53	12.02	4.66	9.36	1510.37
655	80	1628.15	519.41	852.60	109.10	3.99	11.82	5.07	8.14	1510.13
656	125	1628.52	516.45	851.30	110.06	5.42	11.78	5.31	8.03	1508.35
657	132	1628.86	514.94	854.60	110.58	3.15	11.26	5.49	8.21	1508.23
658	139	1629.27	513.67	853.98	112.14	4.15	12.13	5.61	8.45	1510.13
659	210	1629.42	508.87	853.91	113.22	5.11	12.09	5.72	10.14	1509.06
660	245	1629.28	508.01	853.13	114.34	5.70	11.12	5.75	10.25	1508.30
661	306	1629.06	507.44	850.36	115.43	5.15	11.98	5.89	10.37	1506.62
662	411	1629.87	506.68	850.11	117.29	4.84	11.47	5.98	10.49	1506.86
663	445	1629.94	506.54	847.61	119.39	5.56	10.95	6.02	10.54	1506.61
664	500	1631.28	506.41	792.45	166.18	13.71	11.88	6.28	10.85	1507.76
665	560	1636.26	506.11	795.80	167.43	13.96	11.28	6.37	10.18	1511.13
666	605	1640.57	505.49	797.63	167.94	14.65	12.23	6.47	10.37	1514.78
667	711	1641.20	503.61	798.16	169.06	15.14	10.92	6.65	10.94	1514.48

668	1100	1643.65	500.48	793.87	172.89	16.00	12.09	7.26	11.45	1514.04
669	18000	1753.29	494.09	895.89	177.77	18.75	11.89	7.59	15.32	1621.30
670	41	1740.62	523.71	944.02	109.95	3.90	11.19	4.38	10.35	1607.50
671	46	1741.24	515.00	950.98	110.08	4.02	12.16	4.53	10.95	1607.72
672	58	1742.36	513.44	950.94	110.47	4.25	12.26	4.56	12.38	1608.30
673	72	1743.14	508.69	954.90	111.15	4.48	12.00	4.72	13.03	1608.97
674	135	1743.59	506.41	954.47	112.30	4.81	10.78	4.88	15.67	1609.32
675	220	1754.34	506.09	961.10	114.38	5.12	11.83	4.98	16.16	1619.66
676	265	1754.48	505.55	959.51	115.86	5.71	11.29	5.02	16.44	1619.38
677	278	1754.67	504.97	960.48	116.36	4.84	10.92	5.08	16.72	1619.37
678	285	1754.81	503.84	960.35	116.75	5.41	11.03	5.11	16.95	1619.44
679	360	1754.95	502.36	958.30	117.73	5.90	12.22	5.62	17.12	1619.25
680	530	1755.54	501.79	900.71	166.05	16.49	11.36	5.66	17.57	1619.63
681	700	1756.16	501.09	897.87	166.98	17.11	12.28	6.03	17.91	1619.27
682	1120	1760.67	500.37	896.81	168.45	17.92	12.06	6.47	18.42	1620.50
683	1170	1760.78	500.25	895.35	168.74	18.74	12.01	6.61	18.65	1620.35
684	1200	1760.96	498.49	896.77	169.13	19.60	10.71	6.68	19.12	1620.50
685	1300	1761.50	497.98	898.15	170.27	17.12	11.33	6.76	19.37	1620.98
686	1350	1761.72	497.46	898.10	170.45	16.26	11.79	6.81	20.25	1621.12
687	23000	1773.48	493.60	897.22	177.13	17.89	11.20	7.29	24.07	1628.40
688	46	1738.87	533.48	928.19	117.13	4.04	11.49	4.68	10.27	1609.28
689	52	1738.94	529.41	931.17	118.48	3.55	10.96	4.76	10.38	1608.71
690	58	1739.39	527.57	932.35	118.93	3.93	10.31	4.87	10.76	1608.72
691	60	1740.15	524.49	932.79	120.13	3.85	11.78	4.96	11.15	1609.15
692	200	1740.57	513.98	939.03	121.08	4.26	11.82	5.49	13.49	1609.15

693	210	1740.98	511.60	940.08	121.75	4.92	11.97	5.56	13.62	1609.50
694	215	1741.09	506.97	943.50	122.94	4.84	11.26	5.75	14.14	1609.40
695	220	1741.87	506.46	941.69	124.59	5.13	11.98	5.83	14.36	1610.04
696	245	1742.00	506.11	940.30	125.83	5.45	12.09	5.89	14.43	1610.10
697	260	1742.28	505.46	939.16	126.66	5.46	12.42	6.01	14.64	1609.81
698	275	1742.41	504.89	939.46	127.32	5.04	11.98	6.09	14.97	1609.75
699	1050	1747.45	503.45	890.81	167.43	17.02	10.69	6.36	15.93	1611.69
700	1080	1748.61	502.56	889.88	167.82	17.80	12.01	6.48	16.16	1612.71
701	1095	1749.16	501.77	890.28	168.00	17.31	12.68	6.56	16.37	1612.97
702	1120	1749.62	501.09	891.97	168.49	17.13	11.50	6.67	16.44	1613.29
703	1200	1749.84	500.04	890.39	169.03	18.09	12.49	6.78	16.58	1613.40
704	31000	1766.29	488.47	922.98	177.64	18.68	12.38	7.28	15.64	1643.07
705	40	1647.71	529.77	853.15	110.19	4.19	11.82	4.43	11.15	1524.70
706	42	1648.36	526.09	859.74	110.78	4.35	10.52	4.52	11.27	1527.27
707	45	1647.57	523.85	862.12	111.46	4.51	12.55	4.61	11.49	1530.59
708	54	1646.32	520.51	877.43	111.86	3.52	11.88	4.69	12.01	1541.90
709	60	1650.28	516.76	890.49	113.07	3.64	11.28	4.76	12.15	1552.15
710	72	1647.42	513.83	873.16	114.08	3.69	12.11	4.99	12.34	1534.20
711	112	1643.98	508.01	870.16	117.13	4.04	10.92	5.41	12.91	1528.58
712	138	1648.00	506.55	869.71	117.93	4.25	12.09	5.47	13.06	1529.06
713	146	1648.28	505.97	868.39	119.16	4.31	11.99	5.63	13.15	1528.60
714	155	1649.85	505.84	868.91	119.82	4.40	11.19	5.65	13.18	1528.99
715	160	1650.42	505.45	866.30	122.35	4.13	12.16	5.78	13.23	1529.40
716	225	1651.27	504.53	864.73	123.44	4.61	12.26	6.01	13.65	1529.23
717	270	1652.28	503.70	866.67	124.33	4.80	10.72	6.06	13.94	1530.22

718	278	1650.36	502.89	862.55	124.50	5.32	12.18	6.27	14.22	1527.93
719	285	1651.27	501.74	863.34	125.16	5.38	11.83	6.69	14.54	1528.68
720	1550	1682.27	496.63	845.50	166.04	15.31	11.29	7.01	15.18	1556.96
721	32	1605.64	525.84	834.50	106.58	3.29	10.60	4.95	10.75	1496.51
722	38	1607.35	525.10	834.10	107.14	3.89	11.60	4.98	10.78	1497.59
723	45	1608.20	523.97	835.46	107.66	3.91	10.82	5.03	11.12	1497.97
724	47	1608.92	522.86	836.02	107.73	4.05	11.03	5.07	11.34	1498.10
725	55	1608.97	521.61	835.41	108.07	4.08	12.28	5.09	11.49	1498.03
726	61	1609.08	520.64	835.95	108.48	4.14	12.06	5.11	11.57	1497.95
727	74	1609.46	519.45	834.02	111.15	4.21	12.01	5.31	11.65	1497.80
728	80	1609.92	519.16	835.89	112.29	4.29	9.23	5.41	11.71	1497.98
729	225	1610.27	509.58	836.40	116.35	4.39	11.33	6.47	13.36	1497.88
730	265	1610.76	509.40	835.37	117.13	5.00	11.11	6.59	13.67	1498.27
731	460	1610.92	508.97	826.50	123.22	5.14	11.20	7.07	15.03	1497.13
732	470	1611.55	508.48	824.97	123.94	5.39	12.18	7.19	15.21	1497.36
733	550	1612.86	508.16	782.60	156.58	17.36	10.96	7.22	15.61	1498.49
734	680	1613.40	507.13	777.89	162.99	17.40	10.31	7.25	15.75	1498.72
735	785	1614.21	504.70	773.72	167.38	17.74	12.07	7.27	16.03	1498.91
736	820	1614.62	501.57	773.92	168.90	17.79	11.82	7.31	16.15	1497.46
737	865	1617.31	499.04	776.97	169.74	18.10	11.82	7.33	16.31	1499.31
738	910	1623.84	496.99	780.94	172.19	18.21	11.26	7.36	16.47	1503.42
739	55	1630.39	519.98	856.63	113.29	4.04	8.69	4.61	11.68	1518.92
740	1120	1630.77	500.49	791.97	173.03	17.99	12.09	6.85	15.59	1518.01
741	130	1631.17	520.60	846.68	118.12	4.80	12.09	4.63	11.34	1518.26
742	135	1631.65	519.63	846.70	118.89	4.98	11.98	4.68	11.67	1518.53

743	200	1631.85	511.95	848.60	124.36	4.95	10.77	5.52	12.32	1518.47
744	210	1632.06	511.36	847.17	124.82	5.01	12.01	5.56	12.44	1518.37
745	235	1632.31	510.58	847.53	125.30	5.09	11.70	5.67	12.57	1518.44
746	255	1632.77	510.11	847.16	125.67	5.15	12.12	5.78	12.66	1518.65
747	268	1633.36	509.06	848.36	125.87	5.19	11.96	5.91	12.73	1519.08
748	275	1633.49	507.89	850.09	125.99	5.30	10.87	5.96	12.95	1519.05
749	280	1633.87	506.88	848.70	126.80	5.38	11.92	6.08	13.32	1519.08
750	290	1634.15	506.56	848.57	127.16	5.49	11.50	6.29	13.52	1519.09
751	1020	1656.60	504.11	816.73	166.36	17.12	11.99	6.61	15.65	1538.57
752	1035	1657.29	503.43	817.05	166.66	17.33	12.08	6.67	15.76	1538.98
753	1055	1657.82	502.48	817.90	167.04	17.44	11.47	6.76	15.95	1539.04
754	1088	1658.35	501.45	817.39	167.72	17.49	12.29	6.82	16.15	1539.31
755	23000	1684.46	490.94	843.01	177.50	18.35	11.91	7.33	14.02	1563.06
756	40	1612.91	552.11	796.74	117.73	3.91	11.19	4.78	12.36	1498.82
757	45	1613.26	538.49	807.26	119.15	4.26	11.86	4.82	13.01	1498.85
758	48	1613.61	526.86	817.93	120.48	4.28	11.09	4.98	13.33	1498.95
759	101	1613.82	515.05	826.64	121.23	4.35	12.11	5.05	14.48	1498.91
760	136	1614.34	510.97	828.95	122.88	4.40	11.98	5.47	14.69	1499.34
761	145	1614.61	506.51	829.02	126.94	4.52	11.80	5.61	15.14	1499.54
762	150	1614.97	506.39	827.43	128.02	4.69	11.99	5.65	15.62	1499.79
763	155	1615.30	506.28	826.88	128.19	5.11	11.47	5.78	16.33	1500.04
764	160	1615.39	504.60	826.15	128.68	5.19	11.91	5.82	17.71	1500.06
765	1103	1630.77	497.76	801.41	165.38	17.55	12.32	6.96	14.02	1515.40
766	1105	1633.31	497.57	803.91	165.75	17.60	11.81	7.01	14.18	1517.83
767	1009	1634.36	496.90	805.80	166.17	17.62	11.60	7.09	13.67	1518.85

768	1020	1635.57	496.50	805.47	166.34	17.65	12.08	7.18	14.46	1519.68
769	1055	1635.79	495.76	806.56	166.63	17.71	11.73	7.22	14.04	1519.65
770	1065	1635.95	493.95	807.88	167.02	17.79	11.12	7.25	13.65	1518.66
771	1070	1636.20	493.38	806.28	166.99	17.84	11.57	7.35	14.76	1518.17
772	1175	1636.62	493.07	805.92	167.36	18.43	11.93	7.37	14.13	1518.21
773	12000	1685.50	491.50	845.31	176.49	18.90	12.12	7.61	12.55	1564.48
Mean	843.59	1721.02	500.00	866.17	167.11	15.69	11.73	6.93	17.81	1585.44
SD.	2956.38	1380.56	359.29	1233.42	546.19	124.31	35.04	21.06	112.83	1214.41
Max	32000	1803.72	568.70	989.37	187.76	21.40	14.05	8.37	44.68	1657.55
Min	32	1584.74	468.40	614.13	101.84	2.31	8.51	2.56	8.03	1321.1
