# Supplementary material for: Empirical analysis of pig welfare levels and their impact on pig breeding efficiency—Based on 773 pig farmers’ survey data
Source: PLoS One. 2017 Dec 27;12(12):e0190108. doi: 10.1371/journal.pone.0190108 (PMC5744959; doi:10.1371/journal.pone.0190108)
Supplement: S4 File — (PDF) [file pone.0190108.s004.pdf]

S4: variables explained and statistical description

Sample No.	Age	gender (women=0；men=1)	education level (elementary school level and below=1；junior high school=2；high school=3；university or above=4)	breeding years	Revenue (%)	farming scale (small=1; medium-sized=2; large=3)	housed livestock (open=0；enclosed=1)	waste disposal equipment (do not have=0；have=1)	science and technology personnel (insufficient=0；adequate=1)	comprehensive pig welfare level (poor=1；medium=2；good=3；optimal=4)
1	50	1	3	19.08	26.15	1	0	0	1	1
2	55	1	2	17.25	25.72	1	0	0	0	1
3	53	1	3	16.42	27.21	1	0	0	0	2
4	49	0	2	14.5	29.72	1	0	0	0	1
5	54	1	2	12.67	30.40	1	0	0	0	1
6	45	1	3	9.83	51.89	2	1	0	1	2
7	44	0	3	7.92	48.32	2	0	0	0	2
8	43	0	3	10.33	38.52	2	1	0	0	2
9	45	0	3	9.25	39.24	2	1	0	1	2
10	48	1	3	11.17	56.43	2	1	0	0	2
11	44	0	3	12.58	54.89	2	1	0	0	2
12	41	0	2	4.67	66.39	3	0	1	1	3
13	43	1	3	5.83	67.23	3	0	1	1	3
14	44	1	2	9.17	61.98	3	1	1	1	2

15	49	1	3	7.92	66.99	3	1	1	1	2
16	40	1	4	7.42	67.81	3	1	1	1	2
17	37	0	4	6.33	64.61	3	1	1	1	3
18	39	1	3	11.5	69.80	3	1	1	1	4
19	61	1	1	8.58	20.34	1	0	0	1	1
20	55	1	2	13.83	21.04	1	0	0	0	1
21	46	0	2	12.25	21.42	1	0	0	0	2
22	50	1	3	15.17	19.53	1	0	0	0	1
23	41	0	3	6.83	21.23	2	0	0	0	1
24	52	0	2	9.08	27.23	2	0	0	0	1
25	47	1	3	14.75	33.08	2	1	0	1	2
26	44	1	3	12.33	38.35	2	1	0	0	2
27	48	1	3	8.17	43.74	2	1	0	1	2
28	45	0	3	9.83	50.42	2	1	0	1	2
29	44	0	3	8.25	62.52	3	1	1	0	2
30	46	1	3	5.42	71.53	3	0	1	0	2
31	36	1	4	9.83	73.32	3	1	1	1	2
32	40	1	3	9.92	78.52	3	1	1	1	3
33	44	1	3	5.17	76.96	3	0	1	0	3
34	51	0	2	6.33	22.56	1	0	0	0	1
35	58	1	1	11.67	27.62	1	0	0	0	1
36	62	1	1	13.75	23.21	1	0	0	0	1
37	53	0	3	10.08	24.85	1	0	0	0	1
38	55	1	2	6.25	20.24	1	0	0	0	1
39	44	1	3	9.83	21.40	1	0	0	0	2

40	47	0	3	17.42	21.35	1	1	0	0	1
41	51	0	3	15.58	23.20	1	0	0	0	1
42	48	1	3	11.83	23.17	1	0	0	1	1
43	55	1	2	14.25	23.09	2	0	0	0	1
44	50	0	3	13.17	24.02	2	0	0	1	1
45	46	1	3	15.83	25.12	2	1	0	1	2
46	45	1	3	13.08	27.87	2	1	0	0	2
47	37	1	3	10.5	28.31	2	1	0	1	2
48	33	1	3	10.67	41.69	2	1	0	1	2
49	42	1	3	11.33	80.52	3	1	1	1	4
50	49	1	3	5.67	85.71	3	1	1	0	4
51	50	0	2	6.42	20.45	1	0	0	0	1
52	50	0	3	8.42	21.23	1	0	0	0	1
53	52	0	2	9.58	21.44	1	0	0	1	1
54	60	1	1	11.33	22.25	1	0	0	0	1
55	39	0	3	11.75	22.52	1	0	0	1	1
56	45	1	2	9.25	22.62	1	0	0	0	1
57	41	1	3	7.17	23.42	1	0	0	0	2
58	49	1	3	9.83	24.23	1	0	0	0	1
59	48	1	3	11.83	24.73	2	0	0	1	1
60	50	1	3	10.67	28.76	2	1	0	1	2
61	54	1	3	12.42	21.83	2	0	0	0	2
62	40	0	3	13.25	27.37	2	1	0	0	2
63	44	1	3	13.83	42.83	2	1	0	1	2
64	36	1	4	9.08	82.56	3	1	1	0	4

65	43	0	2	15.33	20.47	1	0	0	1	1
66	37	0	3	14	20.25	1	0	0	1	1
67	55	1	3	16.75	19.54	1	0	0	0	1
68	60	1	1	12.17	21.40	1	0	0	1	1
69	59	1	2	14.08	23.90	1	0	0	0	1
70	61	1	1	13.67	24.40	1	0	0	0	1
71	55	1	2	9.58	27.77	1	0	0	0	1
72	47	0	2	6.83	19.66	1	0	0	0	1
73	52	1	3	11.25	20.69	1	0	0	0	2
74	54	1	2	15.17	44.87	2	0	0	0	1
75	50	1	2	16.42	48.79	2	0	0	1	2
76	49	1	3	13.33	51.51	2	0	0	1	2
77	46	1	3	11.75	54.29	2	1	0	1	2
78	41	0	2	15.42	55.63	2	1	0	1	2
79	35	1	3	9.5	55.86	2	1	0	1	2
80	37	0	3	8.33	56.92	2	0	0	1	2
81	42	1	4	5.25	78.21	3	1	1	1	4
82	50	0	2	7.25	24.36	1	0	0	0	1
83	39	0	3	6.58	19.24	1	0	0	1	1
84	44	0	3	11.67	20.95	1	0	0	0	1
85	51	0	2	9.83	22.67	1	0	0	0	1
86	46	0	3	12.17	23.51	1	0	0	0	1
87	47	0	3	15.08	24.19	1	0	0	0	1
88	33	0	3	14.75	24.44	1	0	0	1	1
89	56	1	2	10.83	23.66	2	0	0	0	1

90	49	1	2	9.92	24.11	2	0	0	1	1
91	58	1	1	8.42	27.75	2	0	0	1	2
92	40	1	2	15.25	29.87	2	1	0	1	2
93	46	1	3	13.75	33.07	2	1	0	1	2
94	41	1	3	10.17	53.44	2	1	0	0	2
95	39	1	3	8.33	64.71	3	1	1	1	3
96	45	1	3	9.42	66.42	3	1	1	1	3
97	49	1	3	9.83	68.66	3	1	1	0	3
98	50	1	3	8.25	72.05	3	1	1	1	3
99	63	1	1	7.75	17.02	1	0	0	1	1
100	59	1	1	9.17	17.63	1	0	0	1	1
101	37	0	3	15.33	19.76	1	0	0	0	1
102	45	0	2	15.58	21.60	2	0	0	0	1
103	40	0	2	14.17	25.03	2	0	0	0	2
104	38	1	3	13.83	28.25	2	0	0	0	2
105	36	0	3	13.5	32.48	2	0	1	0	2
106	44	1	3	18.17	34.83	2	1	1	1	1
107	41	1	3	12	37.73	2	1	1	0	2
108	42	1	2	12.5	51.01	2	0	1	1	2
109	48	1	3	12.75	59.49	3	1	1	1	2
110	53	1	2	14.33	60.27	3	1	1	0	2
111	49	1	3	11.25	62.38	3	1	1	1	2
112	47	1	3	11.58	66.91	3	1	1	1	2
113	41	0	4	10.92	67.56	3	1	1	1	2
114	50	1	3	13.25	67.78	3	1	1	1	3

115	52	1	3	13.08	67.12	3	1	1	1	3
116	40	1	4	7.33	90.61	3	1	1	1	4
117	57	0	1	12.5	20.43	1	0	0	0	1
118	61	1	1	17.58	21.18	1	0	0	0	1
119	34	0	3	8.33	22.56	1	0	0	1	1
120	39	0	2	11.42	23.22	1	0	0	0	1
121	50	0	2	14.58	23.40	1	0	0	1	2
122	55	1	2	13.75	24.13	1	0	0	0	1
123	58	1	1	15.83	24.35	1	0	0	0	1
124	56	1	1	15.25	24.73	2	0	0	0	1
125	64	1	1	21.42	24.88	2	1	0	0	2
126	41	1	3	9.25	25.53	2	0	0	1	2
127	45	1	3	8.17	25.76	2	0	0	0	2
128	50	1	3	11.67	27.30	2	1	0	0	2
129	52	1	2	14.42	27.88	2	1	0	1	2
130	39	1	3	6.33	19.64	2	0	0	1	3
131	34	1	3	5.42	20.35	2	0	0	1	2
132	32	1	3	2.75	23.91	2	0	0	0	2
133	48	1	3	10.58	26.84	2	1	0	1	3
134	27	1	4	2.67	80.42	3	1	1	1	4
135	38	0	3	5.75	22.84	1	0	0	1	1
136	60	1	1	16.25	23.22	1	0	0	1	1
137	59	1	1	17.83	23.60	1	0	0	0	1
138	55	1	2	16.92	24.33	1	0	0	1	1
139	49	0	3	15	25.49	1	0	0	0	1

140	58	1	1	17.42	19.89	1	0	0	1	1
141	55	0	1	15.5	21.76	1	0	0	0	2
142	53	0	2	16.58	27.37	2	0	0	0	1
143	54	1	3	14.83	31.39	2	0	0	0	1
144	47	1	3	11.75	32.80	2	0	0	0	1
145	51	1	3	13.42	34.97	2	0	0	1	2
146	49	1	3	16.33	36.76	2	1	0	1	2
147	48	1	3	16.17	46.78	2	1	0	0	3
148	43	0	3	11.83	57.55	3	1	0	1	3
149	45	1	3	18.25	61.72	3	1	0	0	3
150	41	1	3	14.75	72.27	3	1	1	0	3
151	37	1	4	9.83	67.36	3	1	1	1	3
152	34	1	4	6.58	69.73	3	1	1	1	3
153	50	0	2	12.67	27.31	1	0	0	1	1
154	52	0	2	13.75	28.48	1	0	0	0	1
155	56	0	1	14.17	19.70	1	0	0	0	2
156	39	0	3	10.83	20.95	1	0	0	0	1
157	45	0	2	13.25	27.31	2	0	0	0	1
158	51	0	3	16.75	28.59	2	0	0	0	1
159	52	0	1	18.33	28.97	2	0	0	0	1
160	58	1	2	19.92	30.33	2	0	0	0	1
161	46	0	3	12.75	31.39	2	0	0	1	1
162	41	0	2	14.08	37.93	2	0	0	0	2
163	48	0	3	16.17	65.93	3	1	0	1	2
164	52	1	3	18.75	68.75	3	1	0	1	2

165	45	1	3	12.58	69.55	3	1	1	1	2
166	37	1	4	10.5	70.36	3	1	1	1	3
167	52	1	3	18.25	68.25	3	1	1	0	3
168	35	1	4	9.67	68.93	3	1	1	0	3
169	57	1	2	19.83	24.44	1	0	0	1	1
170	51	0	2	17.42	27.51	1	0	0	0	2
171	60	1	1	20.08	28.23	1	0	0	1	1
172	58	0	1	23.5	19.75	1	0	0	1	1
173	53	0	2	17.75	21.70	1	0	0	0	1
174	47	0	2	8.67	26.89	1	0	0	1	1
175	52	0	2	14.58	27.77	1	0	0	0	2
176	53	1	2	15.33	33.87	2	0	0	1	1
177	44	0	3	11.83	41.51	2	0	0	1	2
178	45	1	3	14.92	42.47	2	0	0	1	2
179	30	1	3	7.25	44.90	2	0	0	1	2
180	36	0	3	5.42	49.82	2	0	0	1	2
181	56	1	1	14.67	50.24	2	0	0	1	2
182	51	1	3	15.75	55.60	2	0	0	0	2
183	52	1	2	18.83	56.30	2	1	0	0	3
184	55	1	1	19.42	58.41	2	1	0	1	2
185	46	1	3	15.33	64.45	2	1	0	0	2
186	39	1	4	8.25	79.94	3	1	1	1	4
187	52	1	2	17.25	20.23	1	0	0	1	1
188	58	1	2	15.33	21.44	1	0	0	0	2
189	57	1	2	16.17	22.89	1	0	0	0	1

190	61	1	1	18.33	23.05	1	0	0	0	2
191	63	1	1	22.75	23.54	1	0	0	0	1
192	56	1	1	20.08	24.22	1	0	0	0	1
193	53	0	2	11.83	24.62	1	1	0	0	1
194	58	1	1	17.75	24.51	1	0	0	0	2
195	55	0	1	19.67	25.26	1	0	0	0	1
196	50	0	2	17.42	23.63	2	1	0	1	1
197	49	0	2	13.83	26.21	2	1	0	0	2
198	52	1	3	18	27.48	2	1	0	1	2
199	48	1	3	16.5	33.57	2	1	0	0	2
200	50	1	3	14.25	34.35	2	1	0	0	2
201	46	1	3	13.33	41.33	2	1	0	1	2
202	42	1	3	11.75	49.80	2	1	0	1	2
203	47	0	3	15.17	62.54	2	1	0	1	2
204	39	1	4	14.08	81.57	3	1	1	1	2
205	48	1	2	16.83	22.89	1	0	0	1	1
206	51	1	2	18.33	23.72	1	0	0	1	1
207	59	1	1	19.08	25.25	1	0	0	0	1
208	52	0	2	20.5	18.69	1	1	0	0	1
209	53	0	1	22.33	23.71	1	0	0	0	1
210	56	0	1	21.17	24.32	1	0	0	0	1
211	50	0	2	16.75	25.23	1	0	0	0	2
212	45	0	3	11.67	27.14	1	0	0	0	2
213	48	1	3	14.17	41.40	2	1	0	0	2
214	46	1	3	13.42	42.15	2	0	0	0	1

215	51	1	2	15.58	45.56	2	1	0	0	2
216	47	1	3	16.83	46.43	2	1	0	0	2
217	41	1	3	12.42	48.63	2	1	0	0	2
218	39	1	3	11.33	51.95	2	1	0	1	2
219	49	1	3	18.25	84.35	2	1	0	1	2
220	44	0	3	13.5	58.72	2	1	0	1	3
221	37	1	4	7.5	78.28	3	1	1	1	3
222	62	1	1	17.33	20.58	1	0	0	1	1
223	51	0	2	15.17	21.10	1	0	0	1	1
224	53	0	2	16.67	21.75	1	0	0	1	1
225	59	1	1	20.42	23.32	1	1	0	1	1
226	36	0	3	3.17	23.97	1	0	0	1	2
227	40	0	3	6.67	24.19	1	0	0	1	1
228	52	0	2	14.5	24.95	1	0	0	1	1
229	53	1	2	15.25	21.70	2	0	0	1	1
230	55	1	1	16.08	41.45	2	1	0	1	2
231	50	1	3	15.67	42.30	2	1	0	1	2
232	36	1	3	8.33	45.57	2	0	0	1	2
233	41	1	3	10.25	46.45	2	1	0	0	2
234	46	0	3	12.67	47.34	2	1	0	1	2
235	49	1	3	15.17	49.33	2	1	0	1	2
236	48	1	3	16.83	51.63	2	1	0	0	2
237	45	1	3	12.92	52.96	2	1	0	1	2
238	44	1	3	11.75	78.37	3	1	1	1	3
239	56	1	2	18.67	24.44	1	0	0	0	1

240	57	1	1	15.75	24.93	1	0	0	0	1
241	44	0	2	12.08	25.06	1	0	0	0	1
242	41	0	3	10.67	25.28	1	0	0	0	1
243	58	1	1	20.17	25.66	1	0	0	0	1
244	50	0	2	16.67	26.31	1	1	0	1	1
245	41	0	3	12.75	27.39	1	0	0	1	1
246	46	0	2	13.17	27.53	1	0	0	1	1
247	55	1	2	19.08	27.82	1	0	0	0	2
248	52	1	2	18.67	54.62	2	0	0	0	2
249	53	1	3	14.25	57.34	2	0	0	0	2
250	50	1	3	13.67	61.50	2	1	0	0	2
251	48	1	3	12.92	62.37	2	1	0	0	2
252	44	1	3	11.83	64.25	2	1	0	1	2
253	43	0	2	12.42	66.31	2	1	0	0	2
254	41	1	3	11.33	69.63	2	1	0	1	3
255	49	1	3	16.17	78.87	3	1	1	1	4
256	55	1	2	17.83	22.35	1	0	0	0	1
257	51	0	3	16.58	17.72	1	0	0	0	1
258	53	1	1	17.67	18.97	2	0	0	1	1
259	52	0	2	15.67	19.26	2	0	0	0	2
260	50	0	3	16.33	19.78	2	0	0	1	2
261	54	1	3	18.25	20.67	2	0	0	1	2
262	56	1	1	19.08	31.71	2	0	0	1	2
263	45	0	3	12.75	45.82	2	0	1	1	2
264	51	1	2	16.33	53.50	2	1	1	1	2

265	55	1	2	14.83	61.34	3	1	1	1	2
266	47	1	3	13.92	62.62	3	1	1	0	2
267	34	1	3	8.5	64.42	3	0	1	0	2
268	36	1	4	11.25	65.93	3	1	1	1	3
269	50	1	3	16.75	68.59	3	1	1	0	3
270	48	1	3	15.33	70.71	3	1	1	1	3
271	51	1	3	9.08	91.26	3	1	1	1	4
272	43	1	4	7.33	91.13	3	1	1	1	4
273	52	0	2	16.25	17.63	1	0	0	0	1
274	62	1	1	18.92	18.76	1	0	0	0	1
275	60	1	1	20.17	19.53	1	0	0	0	1
276	59	1	1	18.67	20.25	1	0	0	0	1
277	51	0	2	15.42	21.93	1	0	0	0	1
278	45	0	2	13.33	22.30	1	0	0	0	1
279	40	0	3	5.5	22.97	1	0	0	0	2
280	33	0	3	4.25	23.23	1	0	0	0	1
281	54	1	2	14.33	24.32	1	0	0	1	1
282	47	0	3	13.75	31.85	2	0	0	0	2
283	40	1	3	9.17	32.32	2	0	0	1	2
284	45	0	3	11.08	33.91	2	0	0	1	2
285	51	1	2	15.25	43.06	2	0	0	1	2
286	46	0	3	12.75	43.56	2	0	0	0	2
287	33	0	3	5.17	51.41	2	0	0	0	2
288	49	1	3	16.25	57.64	2	1	0	0	2
289	32	1	4	6.33	66.53	2	0	1	1	3

290	47	1	3	12.83	79.37	3	1	1	1	2
291	29	0	3	3.67	15.72	1	0	0	0	1
292	55	1	1	11.33	17.62	1	0	0	0	1
293	60	1	2	19.17	18.34	1	0	0	0	2
294	51	0	2	13.42	19.55	1	0	0	0	2
295	55	1	2	12.83	20.66	1	0	0	0	2
296	44	0	3	15.5	22.21	1	0	0	0	2
297	46	0	2	13.75	23.70	1	0	0	0	1
298	41	0	3	13.25	40.31	2	0	0	0	1
299	31	0	3	9.08	41.69	2	0	0	0	2
300	52	1	3	17.75	47.81	2	0	0	0	1
301	36	1	3	9.67	53.00	2	0	0	0	2
302	49	1	3	14.33	54.40	2	0	0	0	2
303	45	1	3	12.83	58.62	2	0	0	0	2
304	44	1	3	13.17	62.66	2	0	0	1	2
305	47	1	2	16.92	64.69	2	1	0	0	2
306	52	1	3	18.33	65.76	3	1	1	1	3
307	53	1	2	18.58	67.33	3	1	1	0	3
308	60	1	1	18.92	73.37	3	1	1	1	3
309	41	1	3	12.17	81.55	3	1	1	1	2
310	44	1	3	13.5	14.71	1	0	0	0	1
311	51	1	2	16.75	15.22	1	0	0	0	2
312	61	1	2	20.75	16.75	1	0	0	0	1
313	39	0	3	12.17	17.77	1	0	0	1	2
314	52	0	2	18.42	18.61	1	0	0	0	1

315	35	1	3	5.33	31.85	2	0	0	0	1
316	37	0	3	6.67	45.31	2	0	0	0	2
317	44	1	3	8.17	47.55	2	0	0	0	2
318	50	1	2	13.67	48.53	2	0	0	1	2
319	58	1	2	15.75	51.47	2	0	0	1	2
320	55	1	1	15.25	52.50	2	0	0	0	2
321	53	1	2	16.17	54.99	2	1	0	1	2
322	49	1	3	14.08	66.36	3	1	1	1	3
323	46	1	3	12.67	67.42	3	1	1	1	2
324	36	1	3	9.92	69.76	3	1	1	0	3
325	31	1	3	5.67	70.13	3	0	1	0	2
326	38	1	4	10.33	70.81	3	1	1	1	3
327	62	1	1	12.33	16.59	1	0	0	0	1
328	63	1	1	14.17	17.32	1	0	0	0	1
329	55	1	2	11.08	18.51	1	0	0	0	1
330	49	1	2	8.42	19.90	1	0	0	1	1
331	58	1	1	8.58	20.79	1	0	0	0	2
332	45	0	2	9.83	26.64	2	1	0	0	1
333	40	0	3	7.17	29.76	2	0	0	0	1
334	43	0	3	6.25	32.05	2	0	0	1	1
335	52	1	3	9.33	37.31	2	0	0	0	1
336	53	1	2	8.67	38.55	2	0	0	0	2
337	52	1	2	10.5	41.37	2	0	0	0	2
338	55	1	1	11.25	51.86	2	1	0	1	2
339	41	1	3	7.75	53.30	2	1	0	1	2

340	39	1	3	6.42	54.22	2	0	0	0	2
341	37	1	3	6.17	57.36	2	1	0	1	2
342	49	1	3	13.67	59.03	2	1	0	1	2
343	40	1	3	13.42	71.60	3	1	0	1	3
344	47	1	3	9.33	20.25	1	0	0	0	1
345	41	0	3	8.25	22.94	1	0	0	0	1
346	55	1	2	17.17	23.56	1	0	0	1	2
347	60	1	1	22.17	21.71	2	1	0	0	2
348	50	0	2	13.75	23.47	2	0	0	1	2
349	52	1	2	14.67	31.47	2	1	0	0	2
350	41	1	3	15.25	32.36	2	0	0	1	2
351	46	1	3	13.08	33.25	2	0	0	0	2
352	44	1	3	9.25	40.71	2	0	0	1	2
353	39	1	3	8.5	42.83	2	0	1	1	2
354	52	1	3	13.67	45.90	2	1	1	0	2
355	56	1	2	16.83	51.36	2	1	1	0	2
356	29	1	3	3.67	55.79	2	1	1	1	2
357	52	1	2	15.17	68.42	3	1	1	1	2
358	35	1	3	13.58	71.50	3	1	1	1	3
359	37	1	4	6.17	90.88	3	1	1	1	4
360	41	0	2	6.33	19.26	1	0	0	1	1
361	59	1	1	12.5	18.83	1	0	0	0	1
362	64	1	1	24.83	17.54	1	0	0	0	1
363	51	1	3	15.33	19.66	1	0	0	0	1
364	39	0	3	10.08	21.77	1	0	0	0	1

365	48	1	3	15.17	23.27	1	0	0	0	1
366	58	1	1	16.25	24.90	1	0	0	0	1
367	36	0	3	10.17	18.64	1	0	0	0	2
368	40	0	2	8.33	20.95	1	0	0	0	1
369	53	1	2	13.75	44.87	2	0	0	0	2
370	48	1	3	15.33	46.63	2	0	0	0	2
371	39	1	3	12.08	53.90	2	1	0	0	2
372	45	1	3	13.83	56.47	2	1	0	0	2
373	30	0	3	5.25	59.51	2	0	1	1	2
374	32	1	4	4.58	85.94	3	1	1	1	3
375	43	1	3	5.33	14.73	1	0	0	0	1
376	45	0	3	6.83	15.49	1	0	0	0	1
377	44	0	2	8.17	16.40	1	0	0	0	2
378	44	0	3	8.42	17.63	1	0	0	0	1
379	50	1	2	12.5	18.83	1	0	0	1	1
380	58	1	1	16.83	20.67	1	0	0	1	2
381	36	1	3	5.17	22.90	1	0	0	0	2
382	55	1	2	14.75	23.12	1	0	0	0	1
383	49	0	2	13.67	20.55	1	0	0	0	2
384	50	1	2	15.83	31.24	2	0	0	0	1
385	41	0	3	11.33	50.46	2	0	0	0	2
386	44	1	3	12.5	60.33	2	0	0	1	2
387	46	1	3	13.17	61.72	2	0	0	0	2
388	50	1	2	16.42	64.29	2	1	0	1	2
389	44	1	2	11.08	66.44	2	1	0	1	2

390	36	1	4	8.67	79.44	3	1	1	0	3
391	43	1	3	11.58	17.42	1	0	0	0	1
392	45	0	3	12.17	18.73	1	0	0	1	2
393	44	0	2	13.67	20.06	1	0	0	1	1
394	39	0	3	12.75	20.86	1	0	0	1	1
395	50	1	2	15.08	26.64	1	0	0	1	2
396	52	1	2	12.42	27.31	1	0	0	1	2
397	41	0	3	10.33	28.97	1	0	0	0	2
398	53	1	2	11.42	29.40	1	0	0	0	2
399	50	1	3	13.75	37.34	2	0	0	0	1
400	58	1	1	15.67	44.86	2	0	0	0	2
401	58	1	1	14.25	51.64	2	0	0	0	2
402	59	1	1	18.67	55.90	2	1	0	1	2
403	52	1	3	16.83	57.21	2	1	0	0	2
404	36	1	3	6.75	61.57	2	0	0	1	2
405	37	1	3	8.17	62.65	2	1	0	1	2
406	40	1	3	9.08	65.32	2	1	1	0	2
407	59	1	2	9.33	80.81	3	1	1	1	2
408	62	1	1	15.67	18.65	1	0	0	1	1
409	51	0	2	12.17	18.71	1	0	0	1	1
410	44	0	3	9.08	19.95	1	0	0	1	1
411	53	1	2	13.17	18.30	1	0	0	0	1
412	55	1	2	10.33	17.70	1	0	0	0	1
413	58	1	1	13.42	15.41	1	0	0	0	1
414	41	0	2	8.67	16.29	1	0	0	0	1

415	55	1	1	11.92	18.66	1	0	0	0	2
416	47	0	2	7.75	19.57	1	0	0	0	2
417	50	0	2	12.33	27.33	2	0	0	0	2
418	59	1	1	14.42	28.60	2	0	0	0	2
419	44	0	1	12.75	28.99	2	0	0	0	2
420	52	1	3	13.5	30.44	2	0	0	0	1
421	51	1	3	12	31.80	2	0	0	0	1
422	56	1	2	14.25	33.00	2	0	0	1	1
423	57	1	1	15.33	33.96	2	0	0	0	2
424	49	0	2	6.33	18.61	1	0	0	0	1
425	55	0	2	9.25	18.70	1	0	0	0	1
426	50	0	2	7.17	18.95	1	0	0	0	2
427	46	0	2	10.17	19.50	1	0	0	1	1
428	41	0	3	6.33	14.54	1	0	0	1	1
429	57	1	1	15.25	15.45	1	0	0	0	2
430	60	1	1	16.83	16.27	1	0	0	1	2
431	49	0	2	12.75	17.30	1	0	0	0	2
432	45	0	3	13.17	18.66	1	0	0	0	1
433	53	0	2	18.08	20.00	1	0	0	1	2
434	50	0	2	15.67	27.31	1	0	0	0	1
435	44	0	3	12.17	28.63	1	0	0	0	2
436	55	1	1	17.83	37.92	2	0	0	0	2
437	52	1	2	15.92	55.83	2	0	0	0	2
438	57	1	1	16.33	61.37	2	0	0	0	2
439	45	1	3	12.83	17.62	1	0	0	0	1

440	51	1	3	15.58	18.55	1	0	0	0	1
441	52	1	3	17.42	19.93	1	0	0	0	1
442	57	1	2	18.75	20.59	1	0	0	0	1
443	54	1	2	15.33	21.70	1	0	0	0	1
444	56	1	1	15.42	23.26	1	0	0	0	2
445	57	1	2	15.75	25.50	1	0	0	0	1
446	59	1	1	16.08	28.75	1	0	0	0	2
447	52	1	3	13.25	38.89	2	0	0	0	2
448	55	1	2	14.83	40.40	2	0	0	0	2
449	50	1	2	12.92	51.55	2	1	0	1	2
450	56	1	1	16.75	60.57	2	1	1	1	3
451	51	1	3	13.17	79.91	3	1	1	1	4
452	53	1	2	12.5	22.94	1	0	0	0	1
453	49	0	2	13.17	24.49	1	0	0	0	1
454	46	0	3	12.92	25.62	1	0	0	0	2
455	54	1	2	16.67	34.69	2	0	0	0	2
456	50	1	3	14.17	35.40	2	0	0	0	2
457	45	0	3	12.33	36.34	2	0	0	0	1
458	44	0	3	10.83	44.79	2	0	0	0	1
459	41	0	3	11.08	46.72	2	0	0	0	1
460	46	0	3	13.5	51.56	2	0	0	0	2
461	47	1	3	15.25	62.57	3	1	1	0	2
462	52	1	2	14.75	63.46	3	1	1	1	2
463	53	1	2	16.17	65.53	3	1	1	0	2
464	48	1	3	13.42	65.64	3	1	1	1	3

465	52	1	2	18.75	67.42	3	1	1	1	3
466	50	1	3	19.25	71.35	3	1	1	1	3
467	55	1	3	9.17	91.42	3	1	1	1	4
468	51	1	2	10.33	22.85	1	0	0	0	1
469	58	1	1	12.83	23.61	1	0	0	0	1
470	56	1	1	13.17	24.64	1	0	0	0	1
471	41	0	3	9.67	25.61	1	0	0	0	2
472	45	0	2	5.67	28.69	2	0	0	0	1
473	47	1	3	9.33	31.46	2	0	0	0	1
474	50	1	3	12.17	37.32	2	1	0	0	2
475	56	0	2	13.08	39.30	2	1	0	0	2
476	49	0	2	13.92	46.55	2	1	0	0	2
477	40	1	3	13.5	47.43	2	1	0	0	2
478	35	1	3	6.17	52.55	2	0	0	0	2
479	50	1	3	12.75	55.80	2	1	0	1	2
480	52	1	3	16.67	57.27	2	1	0	1	2
481	56	1	2	15.83	69.50	3	1	1	0	3
482	44	1	3	13.25	69.76	3	1	1	0	3
483	40	1	4	10.17	72.17	3	1	1	0	3
484	59	1	1	12.08	21.68	1	0	0	0	1
485	55	1	2	11.33	22.81	1	0	0	0	1
486	46	0	3	9.58	25.53	1	0	0	1	2
487	53	1	2	11.75	27.31	1	0	0	0	2
488	57	1	1	13.83	41.91	2	0	0	1	2
489	44	0	3	9.92	43.00	2	0	0	1	1

490	41	0	3	8.25	43.69	2	0	0	0	1
491	55	1	2	14.42	49.91	2	0	0	0	2
492	51	1	3	14.67	51.86	2	0	0	1	2
493	40	0	3	11.25	55.79	2	0	0	1	2
494	47	1	2	15.17	57.36	2	0	0	1	2
495	43	0	3	13.83	69.37	2	1	0	0	2
496	51	1	3	15.08	71.51	2	1	0	0	2
497	47	1	3	13.92	71.69	2	1	0	1	2
498	49	1	3	14	72.20	2	1	0	0	2
499	39	0	3	9.25	72.29	3	1	1	1	2
500	44	1	3	12.83	72.47	3	1	1	1	3
501	37	1	4	8.75	80.85	3	1	1	1	3
502	52	1	2	6.33	14.87	1	0	0	0	1
503	54	1	1	7.75	16.32	1	0	0	0	1
504	45	0	2	9.08	24.70	2	0	0	0	2
505	56	1	2	18.25	25.83	2	0	0	0	1
506	53	1	2	15.25	27.64	2	0	0	0	2
507	49	0	2	14.17	28.43	2	0	0	0	2
508	40	0	3	12.33	29.86	2	0	0	0	1
509	54	1	2	16.92	30.77	2	0	0	0	1
510	50	1	3	14.83	31.38	2	1	0	0	2
511	38	0	3	8.75	32.42	2	0	0	1	2
512	45	1	3	12.83	37.66	2	1	0	0	2
513	36	0	3	6.17	52.93	2	1	0	1	2
514	31	1	3	5.42	68.42	3	1	1	1	2

515	37	0	3	9.33	69.49	3	1	1	1	2
516	44	0	2	14.67	69.73	3	1	1	1	3
517	57	0	2	18.17	71.00	3	1	1	1	3
518	52	0	2	16.67	71.85	3	1	1	1	3
519	55	1	1	18.33	68.46	1	0	0	0	1
520	50	1	3	14.25	69.54	1	0	0	1	1
521	56	1	2	18.17	17.09	1	0	0	0	1
522	57	1	2	19.67	17.67	1	0	0	0	1
523	60	1	1	16.75	18.49	1	0	0	0	1
524	44	0	3	13.75	18.76	1	0	0	0	1
525	47	0	2	16.33	20.64	1	0	0	0	1
526	33	0	3	8.25	22.95	1	0	0	0	1
527	36	0	3	9.17	29.66	2	0	0	0	1
528	40	0	3	6.33	31.34	2	0	0	0	2
529	47	0	2	8.75	33.42	2	0	0	1	1
530	52	1	2	16.17	48.65	2	1	0	0	2
531	56	1	3	16.33	52.56	2	1	0	1	2
532	51	1	3	16.83	55.39	2	1	0	0	2
533	40	1	3	17.5	61.55	2	1	0	0	2
534	29	1	4	2.17	68.54	2	0	0	0	2
535	34	1	4	5.42	70.51	3	0	1	1	3
536	38	0	3	6.75	15.99	1	0	0	0	1
537	52	1	3	18.33	18.49	1	0	0	0	1
538	53	1	2	17.83	19.93	1	0	0	0	1
539	62	1	1	23.42	21.84	1	0	0	0	1

540	60	1	1	21.17	22.93	1	1	0	0	2
541	57	1	1	20.33	24.49	1	0	0	0	1
542	46	1	2	16.75	28.60	2	0	0	0	1
543	42	0	3	10.33	33.95	2	0	0	0	2
544	39	0	3	10.17	46.62	2	0	0	0	2
545	44	1	3	11.33	48.46	2	1	0	0	2
546	49	0	2	12.75	51.27	2	1	0	0	2
547	37	1	3	11.42	53.49	2	1	0	0	2
548	44	1	3	14.08	55.33	2	1	0	0	2
549	40	1	3	11.67	59.92	2	1	0	1	2
550	57	1	1	16.25	61.70	2	1	0	1	2
551	55	1	2	18.42	67.30	3	1	1	1	3
552	50	0	3	9.67	18.89	1	0	0	0	1
553	53	1	3	8.17	19.72	1	0	0	0	1
554	51	1	3	10.08	20.83	1	0	0	0	2
555	45	0	2	6.42	22.30	1	0	0	0	1
556	47	1	2	5.67	24.21	1	0	0	0	1
557	51	0	2	9.17	24.83	1	0	0	0	1
558	56	1	2	12.75	29.59	2	0	0	0	1
559	50	1	3	13.17	36.22	2	0	0	0	2
560	48	1	3	12.83	42.92	2	1	0	0	2
561	39	1	3	10.25	49.55	2	1	0	0	2
562	45	0	2	13.42	50.74	2	1	0	0	2
563	41	1	3	9.75	54.37	2	1	0	0	2
564	49	1	3	12.67	56.42	2	1	0	0	2

565	56	1	2	15.5	66.60	2	1	0	1	2
566	50	1	3	14.25	67.51	2	1	0	1	2
567	55	1	2	16.17	68.87	3	1	1	1	3
568	54	1	3	15.08	17.70	1	0	0	0	1
569	50	1	2	14.92	20.36	1	0	0	0	1
570	50	1	2	13.08	21.22	1	0	0	0	1
571	56	1	2	13.67	22.13	1	0	0	0	2
572	49	1	3	13.17	27.37	2	0	0	0	2
573	59	1	1	18.5	32.65	2	0	0	0	1
574	52	1	2	14.25	15.59	2	0	0	0	1
575	50	1	2	13.08	34.41	2	0	0	1	2
576	35	0	3	7.67	35.37	2	0	0	1	2
577	41	0	3	9.25	36.35	2	0	0	0	2
578	42	1	3	11.33	37.73	2	0	0	1	2
579	45	0	3	10.25	42.49	2	0	0	0	2
580	56	1	1	12.33	43.96	2	0	0	0	2
581	47	1	3	12.67	44.21	2	0	0	1	2
582	52	1	3	15.75	66.40	3	1	1	1	2
583	53	1	2	15.67	68.70	3	1	1	1	3
584	56	1	2	16.25	69.65	3	1	1	1	3
585	44	1	3	13.42	71.08	3	1	1	1	3
586	60	1	1	16.5	22.03	1	0	0	0	1
587	56	1	1	14.25	22.55	1	0	0	0	1
588	50	0	2	12.75	23.58	1	0	0	0	1
589	48	0	2	11.33	29.64	2	0	0	0	2

590	55	1	2	16.17	30.90	2	0	0	0	1
591	50	0	2	13.08	32.79	2	0	0	0	1
592	58	1	1	16.83	37.57	2	0	0	0	1
593	51	1	3	14.92	38.70	2	0	0	0	2
594	56	1	2	15.5	39.89	2	0	0	0	1
595	53	1	3	15.17	40.34	2	0	0	0	2
596	45	0	3	13.67	51.10	2	0	0	1	2
597	54	1	3	14.75	66.72	3	1	1	1	2
598	49	0	3	12.83	67.10	3	1	1	1	2
599	45	0	3	13.58	67.64	3	1	1	1	2
600	42	0	3	13.08	68.82	3	1	1	1	3
601	34	1	4	5.83	91.86	3	1	1	1	4
602	39	1	3	3.17	17.77	1	0	0	0	1
603	57	1	1	8.83	18.32	1	0	0	0	1
604	56	1	2	9.08	20.82	1	0	0	0	1
605	57	1	2	7.42	25.49	1	0	0	0	2
606	54	1	3	8.17	31.63	2	0	0	0	2
607	50	1	3	13.75	32.21	2	0	0	1	2
608	47	0	2	9.25	33.29	2	0	0	0	1
609	42	0	3	7.33	33.76	2	0	1	1	2
610	50	1	3	6.42	34.07	2	0	0	0	1
611	55	1	2	8.5	34.60	2	0	0	0	1
612	44	0	2	6.25	35.21	2	0	1	0	2
613	56	1	3	9.17	37.75	2	0	1	0	1
614	50	1	2	11.75	41.32	2	0	1	0	2

615	48	0	2	13.08	49.53	2	0	1	1	2
616	53	1	2	8.42	69.04	3	0	1	1	2
617	51	1	2	12.75	69.97	3	1	1	1	3
618	55	1	3	8.17	91.28	3	1	1	1	4
619	59	1	2	12.83	18.62	1	0	0	1	1
620	62	1	1	14.67	20.69	1	0	0	1	1
621	54	1	2	13.17	33.75	2	0	0	0	2
622	50	1	3	12.67	34.42	2	0	0	1	1
623	45	0	3	10.25	34.77	2	0	0	0	2
624	40	0	3	9.83	35.81	2	0	0	1	1
625	55	1	2	13.42	37.34	2	0	0	1	2
626	50	1	3	12.83	63.79	3	1	1	1	2
627	41	0	3	11.92	64.33	3	1	1	1	2
628	44	1	3	12.5	65.51	3	1	1	1	2
629	49	1	3	14.17	66.06	3	1	1	1	2
630	38	1	4	10.25	66.49	3	1	1	1	3
631	55	1	2	16.25	66.95	3	1	1	1	3
632	48	1	3	13.75	67.35	3	1	1	1	3
633	53	1	2	9.83	91.21	3	1	1	1	4
634	44	0	2	10.17	20.22	1	0	0	0	1
635	60	1	1	19.33	22.89	1	0	0	0	1
636	57	1	1	17.83	23.21	1	0	0	1	1
637	45	0	2	12.83	23.45	1	0	0	1	1
638	37	0	3	10.67	25.50	1	0	0	0	1
639	50	1	2	12.42	26.18	1	0	0	0	1

640	48	0	2	11.25	28.72	1	0	0	0	1
641	54	0	2	16.67	44.81	2	0	0	0	2
642	49	1	3	13.83	48.90	2	0	0	0	1
643	41	0	3	12.75	52.54	2	0	0	1	2
644	47	0	3	14.08	53.25	2	0	0	0	2
645	49	0	3	14.58	53.62	2	0	0	1	2
646	43	0	3	12.67	54.13	2	0	0	1	2
647	55	1	3	15.25	54.70	2	0	0	1	2
648	50	0	2	14.42	53.19	2	0	0	0	2
649	44	0	3	10.83	55.14	2	0	0	0	2
650	57	1	1	16.33	57.39	2	0	0	0	2
651	40	0	3	9.67	64.06	2	0	0	0	2
652	47	0	2	13.08	18.63	1	0	0	0	1
653	56	1	2	12.42	19.19	1	0	0	1	1
654	57	1	1	13.92	19.83	1	0	0	0	1
655	50	0	2	13.17	20.45	1	0	0	1	1
656	58	1	2	13.5	21.34	2	0	0	1	2
657	50	1	3	13.67	22.56	2	0	0	0	1
658	45	0	3	13.17	23.64	2	0	0	0	1
659	49	1	3	14.42	24.93	2	0	0	1	2
660	41	0	3	13.25	25.30	2	0	0	1	2
661	43	1	3	13.83	31.36	2	0	0	1	2
662	39	0	3	14.25	33.95	2	0	0	0	2
663	55	1	2	16.42	34.71	2	0	0	0	2
664	53	1	3	15.33	37.40	2	1	1	1	2

665	50	1	3	14.92	38.83	2	1	1	1	2
666	37	1	4	12	42.51	2	0	1	0	2
667	40	1	4	13.83	47.91	2	0	1	0	2
668	42	1	4	13.17	61.44	3	1	1	1	3
669	45	1	3	7.25	91.63	3	1	1	1	4
670	64	1	1	16.25	18.35	1	0	0	0	1
671	58	1	1	14.67	17.42	1	0	0	0	1
672	55	1	2	14.25	17.76	1	0	0	1	1
673	44	0	3	11.67	18.93	1	0	0	0	1
674	56	1	2	15.42	17.78	2	0	0	1	1
675	51	1	3	14.83	31.82	2	0	0	1	2
676	44	0	3	12.33	33.03	2	1	0	0	2
677	45	0	2	12.17	33.43	2	0	0	0	2
678	39	0	3	11.42	33.90	2	0	0	0	2
679	54	1	2	18.08	36.74	2	1	0	1	2
680	50	1	3	19.92	47.92	2	1	1	0	2
681	43	1	3	14.83	54.35	2	1	1	1	2
682	45	0	4	15.17	61.48	3	1	1	1	2
683	52	1	3	17.67	61.93	3	1	1	1	2
684	47	1	3	13.83	62.52	3	1	1	1	2
685	44	1	3	12.92	64.30	3	1	1	1	3
686	53	1	2	16.75	67.44	3	1	1	1	3
687	50	1	3	10.17	92.16	3	1	1	1	4
688	40	0	2	9.33	16.60	1	0	0	0	1
689	61	1	1	15.42	17.36	1	0	0	0	1

690	38	0	3	6.42	18.55	1	0	0	1	1
691	56	1	2	13.75	19.03	1	0	0	0	1
692	50	0	1	13.83	32.82	2	0	0	0	2
693	57	1	1	14.67	33.22	2	0	0	0	2
694	53	1	3	13.75	27.44	2	0	0	1	1
695	55	1	2	14.25	27.97	2	0	0	1	2
696	47	0	3	13.42	28.76	2	0	0	0	2
697	56	1	2	15.17	29.30	2	1	0	0	2
698	51	1	3	15.08	30.17	2	0	0	0	2
699	53	1	3	14.92	61.20	3	1	1	1	2
700	55	1	3	16.17	61.87	3	1	1	1	2
701	50	1	3	14.33	62.31	3	1	1	1	2
702	48	1	3	15.75	62.42	3	1	1	1	3
703	55	1	2	17.67	64.54	3	1	1	1	3
704	54	1	2	6.42	91.34	3	1	1	1	4
705	54	1	3	11.92	16.21	1	0	0	0	1
706	58	1	1	13.75	16.93	1	0	0	1	1
707	57	1	1	12.83	17.32	1	0	0	0	1
708	57	1	1	13.17	19.55	1	0	0	0	1
709	45	0	3	12.33	20.31	1	0	0	1	1
710	51	1	2	14.67	21.97	1	0	0	0	2
711	56	1	2	15.83	29.66	2	0	0	0	2
712	53	1	3	14.25	30.25	2	0	0	1	2
713	55	1	3	14.67	30.90	2	0	0	1	2
714	57	1	1	15.83	31.40	2	0	0	1	2

715	52	1	3	13.25	32.19	2	0	0	1	1
716	50	0	1	13.42	42.95	2	0	0	0	1
717	54	1	2	14.08	44.32	2	0	0	0	2
718	55	1	2	12.92	44.77	2	0	0	1	2
719	53	0	2	13.67	45.11	2	0	0	0	2
720	48	1	3	13.5	71.25	3	1	1	1	3
721	46	0	3	8.25	18.61	1	0	0	0	1
722	50	0	2	10.33	19.18	1	0	0	0	1
723	48	0	3	6.17	20.22	1	0	0	1	1
724	56	1	1	12.67	20.93	1	0	0	0	1
725	55	1	2	12.83	22.48	1	0	0	1	2
726	47	0	2	11.17	23.59	1	0	0	0	1
727	51	1	3	12.75	25.53	1	0	0	0	1
728	40	0	3	9.83	27.40	1	0	0	0	1
729	53	0	1	13.75	34.87	2	0	0	1	1
730	51	0	2	14.17	36.22	2	0	0	0	2
731	49	1	3	13.67	49.35	2	0	0	1	2
732	54	1	3	14.83	53.44	2	0	0	0	2
733	45	0	3	12.92	56.23	2	0	0	1	2
734	50	1	3	14.25	64.57	2	0	0	0	2
735	44	0	3	12.33	67.34	2	1	0	0	2
736	40	0	3	12.17	63.85	2	1	0	1	2
737	45	1	3	11.67	64.49	2	1	0	0	2
738	56	1	2	13.67	68.61	2	1	0	1	2
739	59	1	1	15.25	24.46	1	0	0	0	1

740	42	0	2	10.42	64.74	3	1	1	1	3
741	45	0	2	9.75	27.77	2	0	0	1	2
742	43	1	3	8.67	29.59	2	0	0	0	1
743	41	0	2	7.33	37.34	2	0	0	1	2
744	38	0	3	9.83	38.69	2	0	0	0	2
745	43	1	3	11.67	39.45	2	0	0	0	2
746	46	0	2	13.42	39.97	2	0	0	0	2
747	39	1	3	12.75	40.26	2	0	0	0	2
748	48	0	2	14.67	40.82	2	0	0	1	2
749	43	0	2	13.17	40.97	2	0	0	0	2
750	44	1	3	12.82	41.12	2	0	0	1	2
751	40	0	3	9.42	61.77	3	1	1	1	2
752	45	1	3	13.25	68.32	3	1	1	0	2
753	41	0	4	14.25	69.37	3	1	1	1	3
754	44	0	3	12.83	70.23	3	1	1	1	3
755	27	1	4	3.42	91.87	3	1	1	1	4
756	59	1	1	13.17	21.54	1	0	0	0	1
757	57	1	2	11.42	22.59	1	0	0	0	1
758	44	1	3	14.25	23.66	1	0	0	1	1
759	40	0	3	8.92	29.62	2	0	0	0	1
760	56	1	2	17.33	31.94	2	0	1	0	1
761	52	0	2	15.08	32.79	2	0	0	0	1
762	51	0	2	16.67	33.20	2	0	0	0	2
763	55	1	1	18.83	33.90	2	0	0	1	1
764	46	0	3	14.25	34.31	2	0	0	0	1

765	33	1	4	9.33	63.73	3	0	1	1	2
766	37	1	4	14.33	63.66	3	1	1	1	2
767	43	1	3	16.17	64.32	3	1	1	1	2
768	34	1	4	12.5	64.91	3	1	1	1	3
769	49	1	3	18.83	65.67	3	1	1	1	3
770	46	1	3	18.42	66.70	3	1	1	1	3
771	37	1	4	15.58	67.85	3	1	1	1	3
772	33	0	4	9.25	67.97	3	1	1	1	3
773	31	1	4	4.5	91.88	3	1	1	1	4
Mean	47.83	0.67	2.46	12.85	64.23	1.83	0.34	0.21	0.43	1.78
SD.	7.23	0.47	0.79	3.75	19.83	0.71	0.47	0.41	0.50	0.74
Max	64	1	4	24.83	92.16	3	1	1	1	4
Min	27	0	1	2.17	14.54	1	0	0	0	1
