# Supplementary material for: Empirical analysis of pig welfare levels and their impact on pig breeding efficiency—Based on 773 pig farmers’ survey data
Source: PLoS One. 2017 Dec 27;12(12):e0190108. doi: 10.1371/journal.pone.0190108 (PMC5744959; doi:10.1371/journal.pone.0190108)
Supplement: S5 File — (PDF) [file pone.0190108.s005.pdf]

S5: Production efficiency results of the 773 pig farmers

firm	crste	firm	crste	firm	crste	firm	crste	firm	crste	firm	crste
1	0.984	130	0.976	259	0.963	388	0.988	517	1	646	0.955
2	0.942	131	0.984	260	0.949	389	0.988	518	0.988	647	0.946
3	0.944	132	0.978	261	0.962	390	0.997	519	1	648	0.944
4	0.948	133	0.972	262	0.947	391	0.949	520	1	649	0.943
5	0.942	134	0.966	263	0.959	392	0.953	521	0.994	650	0.944
6	0.947	135	0.973	264	0.96	393	0.959	522	0.99	651	0.939
7	0.949	136	0.973	265	0.955	394	0.965	523	0.992	652	0.94
8	0.957	137	1	266	0.961	395	0.969	524	0.99	653	0.96
9	1	138	0.985	267	0.954	396	0.978	525	0.993	654	0.967
10	0.978	139	0.966	268	0.956	397	0.971	526	0.99	655	0.986
11	0.973	140	0.959	269	0.954	398	0.976	527	0.995	656	0.986
12	0.956	141	0.96	270	0.957	399	0.979	528	0.995	657	0.995
13	0.959	142	0.955	271	0.962	400	0.986	529	0.984	658	0.98
14	0.973	143	0.948	272	0.965	401	0.988	530	0.989	659	0.944
15	0.993	144	0.948	273	0.983	402	0.977	531	0.997	660	0.943
16	0.972	145	0.95	274	0.979	403	0.983	532	0.99	661	0.941
17	0.981	146	0.962	275	0.995	404	0.984	533	0.98	662	0.938
18	0.978	147	0.973	276	0.968	405	0.985	534	0.982	663	0.941
19	0.948	148	0.963	277	0.979	406	0.99	535	0.987	664	0.947
20	0.938	149	0.964	278	0.962	407	0.997	536	1	665	0.952

21	0.945	150	0.97	279	0.961	408	0.948	537	1	666	0.955
22	0.974	151	0.97	280	0.953	409	0.953	538	0.994	667	0.956
23	0.932	152	0.973	281	0.962	410	0.964	539	0.991	668	0.96
24	0.931	153	0.974	282	0.963	411	0.958	540	0.992	669	0.991
25	0.93	154	0.971	283	0.962	412	0.984	541	0.988	670	0.994
26	0.927	155	0.996	284	0.959	413	0.961	542	0.984	671	0.99
27	0.971	156	0.938	285	0.961	414	0.961	543	0.991	672	0.991
28	0.973	157	0.948	286	0.953	415	0.964	544	0.986	673	0.994
29	0.971	158	0.939	287	0.95	416	0.968	545	0.991	674	0.997
30	0.948	159	0.938	288	0.968	417	1	546	0.987	675	0.994
31	0.956	160	0.946	289	0.976	418	0.971	547	0.988	676	0.994
32	0.957	161	0.939	290	0.964	419	0.969	548	0.987	677	0.996
33	0.955	162	0.949	291	0.969	420	0.979	549	0.989	678	0.996
34	1	163	0.987	292	0.962	421	0.977	550	1	679	0.992
35	0.977	164	0.974	293	0.974	422	0.986	551	1	680	0.979
36	1	165	0.978	294	0.957	423	0.996	552	0.99	681	0.976
37	1	166	0.979	295	0.95	424	0.923	553	0.991	682	0.975
38	0.977	167	0.979	296	0.956	425	0.944	554	1	683	0.974
39	0.971	168	0.98	297	0.974	426	0.939	555	0.998	684	0.981
40	0.982	169	0.974	298	0.974	427	0.938	556	0.995	685	0.976
41	0.974	170	0.967	299	0.97	428	0.959	557	0.996	686	0.976
42	0.965	171	0.952	300	0.971	429	0.94	558	1	687	0.986
43	0.96	172	0.944	301	0.974	430	0.939	559	0.999	688	0.971
44	0.955	173	0.937	302	0.991	431	0.945	560	1	689	0.981
45	0.981	174	0.958	303	0.978	432	0.943	561	1	690	0.985

46	0.947	175	0.996	304	0.968	433	0.94	562	0.987	691	0.964
47	0.964	176	0.948	305	0.969	434	0.947	563	0.982	692	0.969
48	0.961	177	0.962	306	0.966	435	0.939	564	0.983	693	0.97
49	0.953	178	0.953	307	0.967	436	0.946	565	0.987	694	0.979
50	0.94	179	0.957	308	0.965	437	0.952	566	0.987	695	0.974
51	0.961	180	0.933	309	0.966	438	0.954	567	0.993	696	0.973
52	0.939	181	0.931	310	0.965	439	0.959	568	0.933	697	0.974
53	0.945	182	0.937	311	0.966	440	0.952	569	0.935	698	0.974
54	0.934	183	0.963	312	0.962	441	0.953	570	0.938	699	0.977
55	0.955	184	0.968	313	0.962	442	0.952	571	0.94	700	0.975
56	0.964	185	0.973	314	0.961	443	0.955	572	0.941	701	0.975
57	0.993	186	0.969	315	0.971	444	0.948	573	0.944	702	0.976
58	0.914	187	0.978	316	0.968	445	0.953	574	0.942	703	0.977
59	0.922	188	0.975	317	0.967	446	0.957	575	0.942	704	0.999
60	0.932	189	0.957	318	0.972	447	0.97	576	0.943	705	0.939
61	0.955	190	0.948	319	0.975	448	0.968	577	0.983	706	0.943
62	0.962	191	0.948	320	0.977	449	0.96	578	0.944	707	0.94
63	0.963	192	0.943	321	0.965	450	0.967	579	0.94	708	0.943
64	0.957	193	1	322	0.965	451	0.981	580	0.943	709	0.944
65	0.96	194	0.968	323	0.973	452	0.933	581	0.941	710	0.944
66	0.944	195	0.974	324	0.977	453	0.934	582	0.946	711	0.945
67	0.946	196	0.986	325	0.962	454	0.945	583	0.946	712	0.943
68	0.937	197	1	326	0.959	455	0.934	584	0.949	713	0.942
69	0.939	198	0.964	327	0.989	456	0.932	585	0.952	714	0.946
70	0.95	199	0.966	328	0.995	457	0.942	586	0.932	715	0.942

71	0.943	200	0.968	329	0.991	458	0.942	587	0.925	716	0.943
72	0.952	201	0.974	330	0.973	459	0.944	588	0.934	717	0.951
73	0.946	202	0.976	331	0.985	460	0.943	589	0.937	718	0.945
74	0.943	203	0.976	332	0.976	461	0.97	590	0.938	719	0.946
75	0.94	204	0.978	333	1	462	0.963	591	0.94	720	0.96
76	0.936	205	1	334	0.96	463	0.958	592	0.949	721	0.939
77	0.949	206	1	335	0.958	464	0.965	593	0.949	722	0.932
78	0.94	207	0.955	336	0.98	465	0.964	594	0.95	723	0.933
79	0.939	208	0.963	337	0.969	466	0.977	595	0.951	724	0.933
80	0.944	209	1	338	0.966	467	0.981	596	0.945	725	0.933
81	0.953	210	0.958	339	0.959	468	1	597	0.949	726	0.933
82	1	211	0.952	340	0.962	469	0.983	598	0.951	727	0.928
83	0.987	212	0.94	341	0.956	470	0.978	599	0.954	728	0.944
84	0.979	213	0.955	342	0.964	471	0.971	600	0.955	729	0.928
85	0.965	214	0.973	343	0.966	472	0.994	601	0.986	730	0.929
86	0.967	215	0.956	344	0.993	473	0.964	602	0.96	731	0.925
87	0.967	216	0.955	345	0.991	474	0.975	603	0.958	732	0.925
88	0.969	217	0.957	346	0.994	475	0.979	604	0.96	733	0.919
89	0.983	218	0.972	347	1	476	0.988	605	0.96	734	0.927
90	0.957	219	0.97	348	0.992	477	0.993	606	0.967	735	0.926
91	0.957	220	0.975	349	1	478	1	607	0.959	736	0.93
92	0.954	221	0.982	350	0.988	479	0.976	608	0.961	737	0.934
93	0.954	222	0.958	351	0.985	480	0.976	609	0.963	738	0.939
94	0.964	223	0.944	352	0.997	481	0.985	610	0.964	739	0.966
95	0.962	224	0.943	353	0.991	482	0.986	611	0.964	740	0.937

96	0.96	225	0.954	354	0.995	483	0.986	612	0.959	741	0.923
97	0.959	226	0.953	355	0.97	484	0.969	613	0.952	742	0.923
98	0.955	227	0.942	356	0.974	485	0.989	614	0.949	743	0.933
99	0.939	228	0.938	357	0.98	486	0.972	615	0.949	744	0.927
100	0.948	229	0.943	358	0.981	487	0.964	616	0.954	745	0.928
101	0.923	230	0.95	359	0.975	488	0.972	617	0.956	746	0.928
102	0.922	231	0.956	360	0.989	489	0.955	618	0.987	747	0.93
103	0.916	232	0.979	361	0.987	490	0.976	619	0.933	748	0.936
104	0.94	233	0.985	362	0.983	491	0.97	620	0.93	749	0.932
105	0.959	234	0.971	363	0.983	492	0.98	621	0.928	750	0.933
106	0.943	235	0.98	364	0.981	493	0.976	622	0.935	751	0.941
107	0.952	236	0.978	365	0.985	494	0.977	623	0.935	752	0.942
108	0.957	237	0.977	366	0.983	495	0.981	624	0.937	753	0.942
109	0.951	238	0.975	367	0.986	496	0.973	625	0.938	754	0.943
110	0.954	239	1	368	0.998	497	0.98	626	0.941	755	0.975
111	0.956	240	0.961	369	0.988	498	0.978	627	0.944	756	0.893
112	0.959	241	0.959	370	0.976	499	0.983	628	0.946	757	0.9
113	0.954	242	0.945	371	0.984	500	0.988	629	0.948	758	0.911
114	0.961	243	0.961	372	0.984	501	0.991	630	0.951	759	0.919
115	0.95	244	0.954	373	1	502	1	631	0.954	760	0.923
116	0.952	245	0.955	374	1	503	0.998	632	0.955	761	0.929
117	0.969	246	0.975	375	0.981	504	0.991	633	0.984	762	0.929
118	0.961	247	1	376	0.973	505	0.99	634	0.941	763	0.928
119	0.964	248	0.979	377	0.978	506	0.994	635	0.938	764	0.931
120	0.963	249	0.961	378	0.972	507	1	636	0.933	765	0.945

121	0.964	250	0.967	379	0.975	508	0.991	637	0.932	766	0.945
122	0.96	251	0.975	380	0.969	509	0.989	638	0.933	767	0.948
123	0.958	252	0.979	381	0.968	510	0.984	639	0.935	768	0.946
124	0.958	253	0.977	382	0.967	511	0.99	640	0.941	769	0.949
125	0.951	254	0.982	383	0.974	512	0.972	641	0.946	770	0.953
126	0.961	255	0.981	384	0.988	513	0.984	642	0.946	771	0.95
127	0.962	256	0.958	385	0.982	514	0.978	643	0.948	772	0.953
128	0.973	257	0.973	386	0.971	515	0.985	644	0.947	773	0.98
129	0.975	258	0.957	387	0.974	516	0.99	645	0.947		
